# Mutations in Membrin/*GOSR2* Reveal Stringent Secretory Pathway Demands of Dendritic Growth and Synaptic Integrity

**DOI:** 10.1016/j.celrep.2017.09.004

**Published:** 2017-10-03

**Authors:** Roman Praschberger, Simon A. Lowe, Nancy T. Malintan, Carlo N.G. Giachello, Nian Patel, Henry Houlden, Dimitri M. Kullmann, Richard A. Baines, Maria M. Usowicz, Shyam S. Krishnakumar, James J.L. Hodge, James E. Rothman, James E.C. Jepson

**Affiliations:** 1 Department of Clinical and Experimental Epilepsy, UCL Institute of Neurology, London, UK; 2 School of Physiology, Pharmacology, and Neuroscience, University of Bristol, Bristol, UK; 3 Faculty of Biology, Medicine, and Health, Division of Neuroscience & Experimental Psychology, Manchester Academic Health Science Centre, University of Manchester, Manchester, UK; 4 Department of Molecular Neuroscience, UCL Institute of Neurology, London, UK; 5 Department of Cell Biology, Yale School of Medicine, New Haven, CT, USA

**Keywords:** Membrin, *GOSR2*, GS27, progressive myoclonus epilepsy, dendrite growth, synaptic integrity

## Abstract

Mutations in the Golgi SNARE (SNAP [soluble NSF attachment protein] receptor) protein Membrin (encoded by the *GOSR2* gene) cause progressive myoclonus epilepsy (PME). Membrin is a ubiquitous and essential protein mediating ER-to-Golgi membrane fusion. Thus, it is unclear how mutations in Membrin result in a disorder restricted to the nervous system. Here, we use a multi-layered strategy to elucidate the consequences of Membrin mutations from protein to neuron. We show that the pathogenic mutations cause partial reductions in SNARE-mediated membrane fusion. Importantly, these alterations were sufficient to profoundly impair dendritic growth in *Drosophila* models of *GOSR2*-PME. Furthermore, we show that Membrin mutations cause fragmentation of the presynaptic cytoskeleton coupled with transsynaptic instability and hyperactive neurotransmission. Our study highlights how dendritic growth is vulnerable even to subtle secretory pathway deficits, uncovers a role for Membrin in synaptic function, and provides a comprehensive explanatory basis for genotype-phenotype relationships in *GOSR2*-PME.

## Highlights

• Epilepsy/ataxia-linked mutations in Membrin confer partial Golgi SNARE defects
• Partial reductions in membrane trafficking strongly impair dendritic growth
• Synaptic morphology and function tightly depend upon efficient secretory trafficking

## Graphical Abstract

**Figure:**
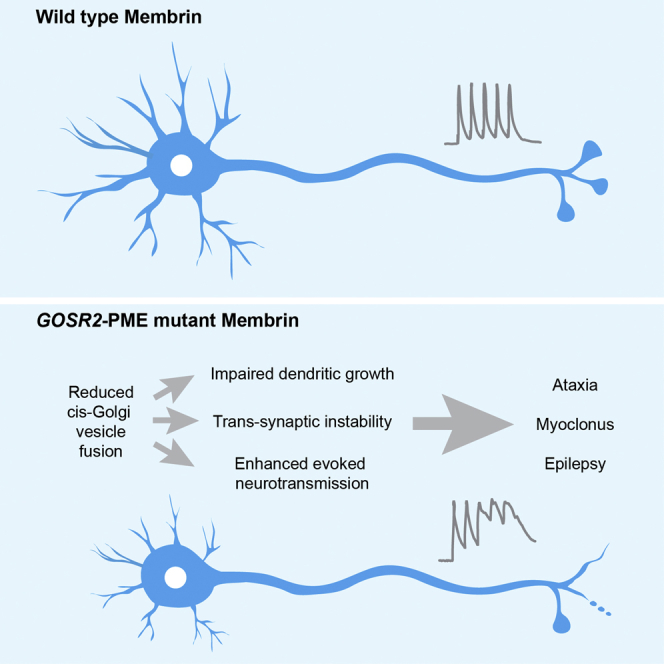

## Teaser

In this study, Praschberger et al. utilize in vitro assays, patient-derived cells, and *Drosophila* models to unravel how mutations in the essential Golgi SNARE protein Membrin cause progressive myoclonus epilepsy and to demonstrate a selective vulnerability of developing neurons to partial impairment of ER-to-Golgi trafficking.

## Introduction

Secreted, membrane, endosomal, and lysosomal proteins are deposited into the endoplasmic reticulum (ER) after ribosomal synthesis. Subsequently, these proteins exit the ER, transition through the Golgi apparatus, and reach their ultimate target sites via the trans-Golgi network, a biosynthetic route termed the secretory pathway (Palade, 1975). The transport of proteins along this path is facilitated by membrane-enclosed vesicles, and their fusion with the *cis*-Golgi is mediated by the target (t-) SNARE (SNAP [soluble NSF attachment protein] receptor) proteins Membrin (also known as GS27; encoded by the *GOSR2* gene), Sec22b, and Syntaxin-5, in concert with the vesicle (v-) SNARE Bet1 (Parlati et al., 2000, Xu et al., 2000). Similar to other intracellular fusion steps, these proteins are necessary for fusion of opposing lipid bilayers through the formation of a quaternary SNARE complex (Hay et al., 1997, Lowe et al., 1997, Parlati et al., 2000, Volchuk et al., 2004). Critical to this process is the N- to C-terminal zippering along 15 mostly hydrophobic “layer” amino acids (−7 to +8) within the SNARE domain of each protein (Gao et al., 2012, Sutton et al., 1998).

Homozygous missense (G144W: layer −3) or compound heterozygous missense and deletion mutations (G144W and K164del: between layer +2 and +3) in the Membrin SNARE motif have recently been shown to cause the severe neurological syndrome progressive myoclonus epilepsy (PME) (Corbett et al., 2011, Praschberger et al., 2015). Patients with this form of PME, termed *GOSR2*-PME, typically present with ataxia at ∼3 years of age, followed by cortical myoclonus and generalized tonic-clonic seizures. Despite rapid disease progression and frequent premature death, cognitive function usually remains remarkably preserved. Correspondingly, marked neurodegeneration as an underlying primary cause has not been reported (Boissé Lomax et al., 2013, Corbett et al., 2011, Praschberger et al., 2015, van Egmond et al., 2014, van Egmond et al., 2015). Given the critical role of Membrin in ER-to-Golgi trafficking and its fundamental importance in every cell of the human body, it is unclear why Membrin mutations specifically result in nervous system dysfunction and do not cause symptoms in other organs. No paralog is present in the human genome that could functionally replace Membrin in non-neuronal cells and therefore explain the primarily neuronal phenotype.

In the present study, we set out to unravel the neuronal bottleneck of *GOSR2*-PME. To do so, we investigated the disease mechanism of *GOSR2*-PME from molecule to neuron utilizing reconstituted liposome fusion assays, patient-derived fibroblasts, and *Drosophila* models. We found that the pathogenic Membrin SNARE motif mutations result in a partial loss of function that is nonetheless sufficient to robustly reduce dendritic growth in vivo. Membrin mutations also resulted in presynaptic retraction and physiological abnormalities at motor synapses. Together, our results suggest a mechanistic basis for the multifaceted neurological features of *GOSR2*-PME patients, highlight tight trafficking demands of growing dendrites, and illustrate a close-knit dependence of synaptic integrity and neurotransmitter release on cargo trafficking through the Golgi apparatus.

## Results

### *GOSR2*-PME Mutations Result in Partial SNARE Dysfunction

**Figure 1:**
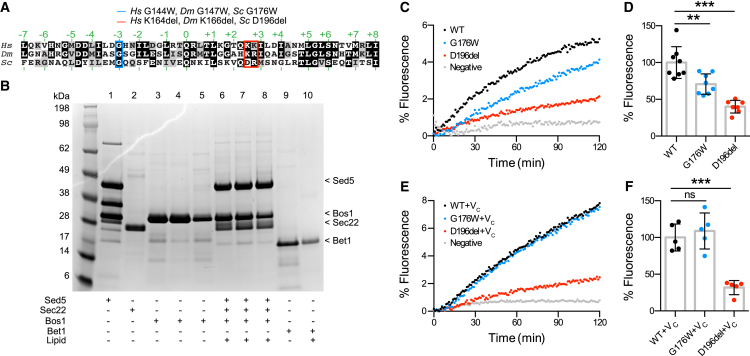
Reduced Liposome Fusion Rates due to Orthologous *GOSR2*-PME Mutations (A) SNARE domain alignment of *Homo sapiens* (*Hs*), *Drosophila melanogaster* (*Dm*), and *Saccharomyces cerevisiae* (*Sc*) Membrin (UniProt: O14653-1), Membrin (UniProt: Q9VRL2), and Bos1 (UniProt: P25385), respectively. Layer amino acids critical for forming the tetrameric *cis*-Golgi SNARE complex are indicated in green. The disease-causing G144W and K164del (one of two consecutive lysines is deleted) and the *Drosophila* and yeast orthologous residues are highlighted in blue and red. (B) Yeast Golgi SNARE proteins Sed5 (lane 1), Sec22 (lane 2), WT Bos1 (lane 3) and G176W/D196del Bos1 mutants (lane 4/5) were purified and reconstituted into acceptor liposomes as t-SNARE complexes comprised of Sed5/Sec22/Bos1 (lanes 6/7/8, respectively). Overall stoichiometry of Sed5/Sec22/Bos1 was ∼1.0×/1.0×/1.2× (Figure S1). Yeast Golgi SNARE protein Bet1 was purified (lane 9) and reconstituted into donor liposome (lane 10) containing 7-nitro-2-1,3-benzoxadiazol-4-yl (NBD)-phosphoethanolamine (PE) and rhodamine-PE fluorescent lipids. (C) Example traces showing increase in NBD fluorescence due to fusion between WT or G176W/D196del Bos1-containing t-SNARE complex acceptor liposomes and Bet1 donor liposomes. Data are expressed as a fraction of maximal NBD fluorescence after addition of detergent. (D) Endpoint (120 min) quantification of experiment as described in (C), normalized to WT. n = 8, 8, 7 for WT, G176W and D196del. (E) Example traces of experiment as in (C) with the modification that 50 μM of a peptide comprising the C-terminal half of the Bet1 SNARE domain (V_C_) was added. (F) Endpoint (120 min) quantification of experiment as described in (E), normalized to WT (n = 5). Replicate values, mean, and SD are shown. ^∗∗^p < 0.01; ^∗∗∗^p < 0.001; ns, not significant (p > 0.05); one-way ANOVA with Dunnett’s multiple comparison test.

The locations of the PME-causing G144W and K164del mutations in the Membrin SNARE domain suggest defective assembly of the quaternary *cis*-Golgi SNARE complex and thus reduced fusion of vesicular cargo carriers with this compartment. Given the technical difficulties associated with producing mammalian Golgi SNAREs, and since mammalian and yeast Golgi SNAREs are functionally conserved (McNew et al., 1997, Fischer von Mollard and Stevens, 1998, Varlamov et al., 2004), we tested for SNARE defects using a well-established yeast SNARE protein liposome fusion assay (McNew et al., 2000, Parlati et al., 2000) (see Supplemental Experimental Procedures for details). We introduced the corresponding PME-linked G176W/D196del mutations into the Membrin yeast ortholog Bos1 (Figure 1A). Purified t-SNAREs containing Bos1, Sec22, and Sed5 (orthologous to mammalian Syntaxin-5) were subsequently preassembled and reconstituted into acceptor liposomes, while the v-SNARE Bet1 was incorporated into the fluorescent donor liposomes (Figures 1B and S1A).

Both PME mutations resulted in a reduced rate and extent of fusion compared to wild-type (WT), but fusion rates were significantly higher relative to a negative control where Bet1 was omitted (Figures 1C and 1D). The relative magnitude of the effects of the D196del and G176W mutations (∼60% and 30% reductions in fusion, respectively) is consistent with their positions within the SNARE motif. The D196 deletion likely results in misalignment of the subsequent hydrophobic layers in the C-terminal half of the SNARE domain, a region that provides the critical force to drive membrane fusion (Gao et al., 2012). In contrast, the more subtle effect of the G176W mutation is consistent with an alteration in the N-terminal SNARE region that mediates the initial engagement of SNARE domains bridging two opposing lipid bilayers. Indeed, in accordance with a selective N-terminal assembly defect, the effect of G176W but not D196del was rescued by addition of a peptide comprising of the C-terminal half of the Bet1 SNARE domain, which acts to pre-structure the N terminus (Figures 1E and 1F) (Melia et al., 2002). Increasing the pool of preassembled trans-SNARE complexes by overnight pre-incubation at 4°C also restored the fusion capacity of G176W-Bos1-containing, but not D196del-Bos1-containing, liposomes (Figures S1B and S1C). Taken together, these results suggest that the orthologous G144W and K164del mutations in Membrin partially impair distinct steps of the *cis*-Golgi SNARE complex formation, which is necessary for fusion of vesicular cargo carriers with the *cis*-Golgi (Hay et al., 1997, Hay et al., 1998).

### Mutant Membrin Retains the Capability to Localize to the *cis*-Golgi

**Figure 2:**
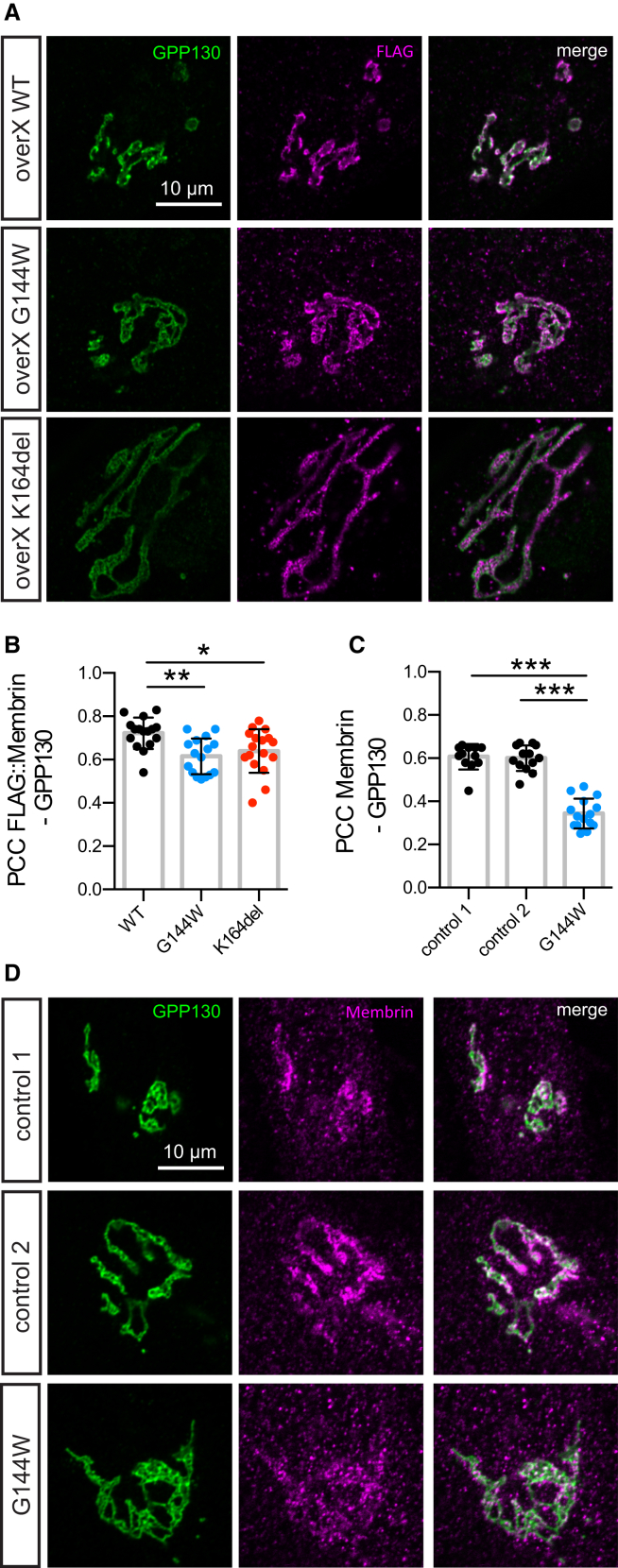
Mutant Membrin Retains the Capability to Localize to the *cis*-Golgi (A) FLAG-tagged WT and G144W/K164del mutant Membrin were overexpressed in control fibroblasts and co-stained for the FLAG tag and the *cis*-Golgi resident protein GPP130. Example confocal slices are shown for each overexpressed construct. (B) Pearson’s correlation coefficients between FLAG and GP130 signals of the experiment described in (A) are shown. n = 16, 16, and 17 for WT, G144W, and K164del. (C) Pearson’s correlation coefficients between endogenous Membrin and GPP130 signals of the experiment described in (D) are shown. n = 12, 13, and 15 for control 1, control 2, and G144W. (D) Example confocal slices of control and Membrin G144W mutant fibroblasts co-stained for endogenous Membrin and GPP130. Replicate values, mean and SD are shown. ^∗^p < 0.05; ^∗∗^p < 0.01; ^∗∗∗^p < 0.001; one-way ANOVA with Dunnett’s multiple comparison test.

Only Membrin localized to the *cis*-Golgi will be capable of facilitating deposition of ER-derived cargo. Thus, we assessed the subcellular localization of overexpressed WT and G144W/K164del mutant FLAG::Membrin in primary skin fibroblasts from a healthy human control. Similar to WT, both mutants exited the ER and co-localized with the *cis*-Golgi matrix protein GPP130 (Figures S2A, S2B, 2A, and 2B). Previously, it was reported that G144W mutant Membrin failed to localize to the *cis*-Golgi in a patient derived fibroblast line (Corbett et al., 2011). We therefore re-examined these cells with an experimentally validated anti-Membrin antibody (Figures S2C and S2D). Membrin could clearly be detected at the *cis*-Golgi of G144W mutant fibroblasts and did not appear to accumulate in the ER, confirming the above overexpression results in patient cells (Figures 2C, 2D, and S2E). We note that both Golgi-localized and overall Membrin levels were reduced in the single *GOSR2*-PME patient cell line (Figures S2F–S2H). However, there was also substantial variability in Membrin levels between healthy control lines (Figures S2F–S2H). Thus, from the above data, we conclude that both the G144W and K164del mutant forms of Membrin retain the capability to localize to the *cis*-Golgi target compartment. This suggests that the partial SNARE domain deficiencies found in liposome fusion assays are relevant to lipid bilayer fusion rates at the *cis*-Golgi.

### Early Lethality and Locomotor Defects in *Drosophila* Models of *GOSR2*-PME

**Figure 3:**
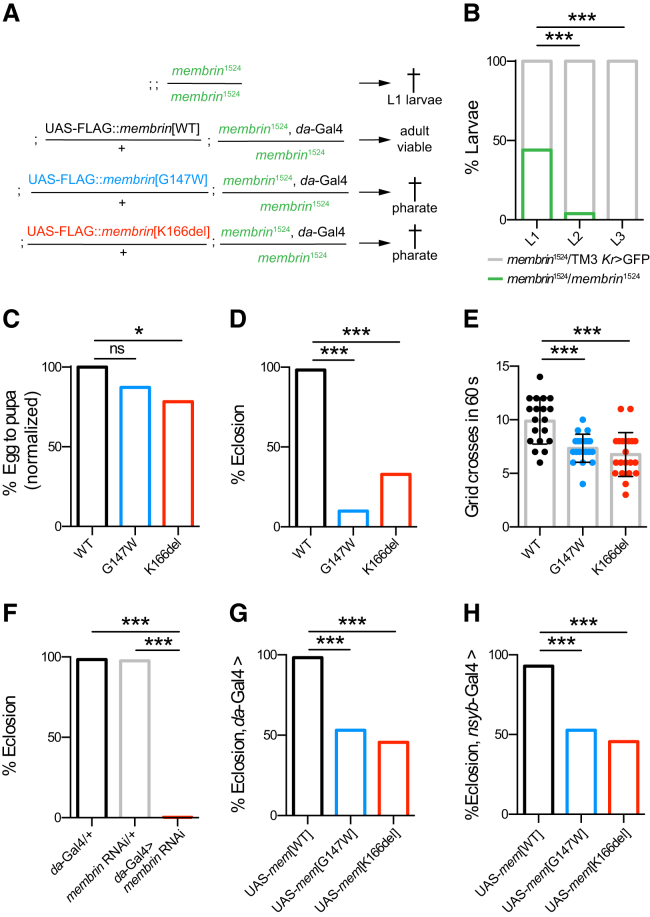
Membrin Mutations Cause Early Lethality and Locomotor Defects in *Drosophila* (A) Genotypes of the *GOSR2*-PME *Drosophila* model used in this study. FLAG-tagged WT or mutant *membrin* (harboring the orthologous G147W/K166del mutations) is globally expressed via the *daughterless*-Gal4 driver in a *membrin*-null (*membrin*^1524^) background. The shorthand Mem-WT, Mem-G147W, and Mem-K166del is used throughout the paper. (B) The *membrin*^1524^ allele was balanced over the fluorescently labeled TM3 Kr > GFP chromosome to discern heterozygote animals. Homozygosity for *membrin*^1524^ caused largely L1 lethality, as at the L2 stage, hardly any non-GFP-positive larvae were detected. n = 50, 53, and 56 for L1, L2, and L3 larvae. (C) Global expression of WT, G147W, and K166del mutant Membrin rescued *membrin*-null *Drosophila* to the pupal stage. Data are expressed relative to Mem-WT. n = 1,222, 1,308, and 1,260 eggs/embryos for Mem-WT/-G147W/-K166del. (D) Mem-G147W and Mem-K166del *Drosophila* exhibited a drastic decrease in eclosion rates compared to Mem-WT. n = 120, 112, and 97 for Mem-WT/-G147W/-K166del non-*tubby* pupae. (E) Freely moving Mem-G147W and Mem-K166del L3 larvae crossed fewer 4-mm grids in 60 s than Mem-WT larvae. n = 19, 20, and 21 for Mem-WT/-G147W/-K166del. Replicate values, mean, and SD are shown. (F) Global *membrin* RNAi-mediated knockdown caused pharate adult stage lethality. n = 378, 162, and 313 for *da*-Gal4 driver and *membrin* RNAi transgene only controls and experimental knockdown. (G) Global overexpression of mutant UAS-*membrin* in WT Membrin animals with *da*-Gal4 resulted in reduced eclosion. n = 284, 514, and 403 for UAS-*membrin*[WT]/[G147W]/[K166del]. (H) Neuronal overexpression of mutant UAS-*membrin* in WT Membrin animals with *nsyb*-Gal4 resulted in reduced eclosion. n = 616, 491, and 618 for UAS-*membrin*[WT]/[G147W]/[K166del]. ^∗^p < 0.05; ^∗∗^p < 0.01; ^∗∗∗^p < 0.001; ns, not significant (p > 0.05); Fisher’s exact test with Bonferroni correction (B–D and F–H) or one-way ANOVA with Dunnett’s multiple comparison test (E).

We next sought to study the effects of Membrin mutations in vivo using *Drosophila melanogaster*. Golgi SNARE proteins are highly conserved throughout evolution (Kloepper et al., 2007), and the *Drosophila* genome contains a single ortholog of the Membrin-encoding *GOSR2* gene (*membrin*, encoding the protein Membrin). Consistent with an essential role for Membrin orthologs in eukaryotes (Shim et al., 1991), homozygosity for the *membrin*-null allele *membrin*^1524^ resulted in lethality largely prior to the L2 larval stage (Figures 3A and 3B) (Ghabrial et al., 2011).

To assess the effects of *GOSR2*-PME mutations in *Drosophila*, we generated transgenic fly lines harboring FLAG-tagged WT or mutant (G144W or K164del) upstream activating sequence (UAS)-*GOSR2* transgenes. Each transgene sequence was integrated at the same genomic locus using site-specific ΦC31-mediated recombination to control for position effects on expression levels (Figure S3A) (Bischof et al., 2007). Expression of WT human *GOSR2* in a *membrin*-null background using the global *daughterless*-Gal4 driver fully rescued the lethality of *membrin*-null larvae and yielded adults that appeared morphologically normal (Figures S3B and S3C). While these adult animals exhibited severe motor impairments and usually died after 3 days, this result nonetheless demonstrates functional conservation between human and *Drosophila* Membrin, supporting the use of *Drosophila* to model *GOSR2*-PME.

Because neither mutant *GOSR2* transgene rescued *membrin*^1524^ animals to the L3 larval stage (data not shown), we next generated *GOSR2*-PME models that were closer to the normal physiology of *Drosophila*. Using an identical strategy, we created WT and mutant (G147W and K166del) *Drosophila* UAS-*membrin* transgenes and expressed them in a *membrin*-null genetic background. For simplicity, we term these mutant fly lines and their associated control Mem-G147W, Mem-K166del, and Mem-WT (Figure 3A).

In contrast to *membrin*-null flies, Mem-WT flies were viable to the adult stage (Figures 3C and 3D). Mem-G147W and Mem-K166del flies were viable to the pupal stage, surviving significantly longer than *membrin-*null animals (Figure 3C). However, Mem-G147W and Mem-K166del flies frequently died within the pupal cases as fully developed pharate adults (Figure 3D). When manually released from the pupal case, Mem-G147W and Mem-K166del adults appeared weak and uncoordinated. Mutant animals that successfully freed themselves from their pupal cases usually became quickly stuck in fly food and died within a few days. Mem-G147W and Mem-K166del L3 larvae also displayed significantly reduced rates of locomotion compared to Mem-WT larvae (Figure 3E). Locomotor deficits and lack of coordination may thus explain the frequent inability of adult Mem-G147W and Mem-K166del flies to escape from the pupal case, leading to early lethality.

Consistent with a partial loss-of-function disease mechanism conferred by the pathogenic *GOSR2*-PME mutations, reducing Membrin levels via transgenic RNAi also dramatically decreased eclosion rates (Figure 3F). Co-overexpression of *membrin* RNAi with WT *Drosophila* UAS-*membrin* or WT human UAS-*GOSR2* transgenes resulted in a full rescue of eclosion deficits relative to a control transgene (UAS-*GCaMP6m*) (Figure S3D), confirming that the observed phenotype is specific to Membrin and further reinforcing the high degree of functional conservation between *Drosophila* and human Membrin.

Interestingly, global overexpression of UAS-*membrin*[G147W] and UAS-*membrin*[K166del] in WT flies similarly caused pharate adult lethality, albeit to a slightly weaker degree to that observed in the *GOSR2*-PME *Drosophila* model, where no endogenous Membrin is present (Figure 3G). This phenomenon is likely due to outcompetition of endogenous WT Membrin by the overexpressed mutant isoforms, and it suggested to us that overexpression of mutant Membrin could serve as a tool to test whether nervous system dysfunction is at the core of the observed *Drosophila* phenotypes. Indeed, overexpression of UAS-*membrin*[G147W] and UAS-*membrin*[K166del] selectively in neurons using two independent driver lines (*nsyb*-Gal4 and *elav*-Gal4) phenocopied the eclosion defects arising from global overexpression in WT flies (Figures 3H and S3E). Taken together, the incomplete rescue of *membrin*-null flies by G147W and K166del mutant Membrin provides in vivo evidence that these mutations cause partial loss of function, a postulate consistent with the above liposome fusion assays and supported by similar eclosion deficits due to Membrin knockdown. Furthermore, our overexpression data suggest that neuronal dysfunction is at the center of the observed organismal *Drosophila* phenotypes, in agreement with the almost exclusively neuronal phenotype of *GOSR2*-PME patients.

### Profound Dendritic Growth Deficits in *GOSR2*-PME Model Neurons

The early onset of symptoms in *GOSR2*-PME suggests that Membrin mutations might alter aspects of neuronal development. Important clues toward the underlying mechanism stem from studies investigating neuronal consequences of mutations in other ER-to-Golgi trafficking proteins. Overexpression of GTP-locked Q71L-Arf1 in cultured hippocampal neurons leads to severely impaired dendritic growth (Horton et al., 2005). Similarly, Ye et al. (2007) found in a *Drosophila* forward genetic screen that mutations in Sec23, Sar1, and Rab1 cause dendritic growth deficiencies, likely by preventing ER-derived lipids and proteins from reaching the plasmalemma of growing neurons.

**Figure 4:**
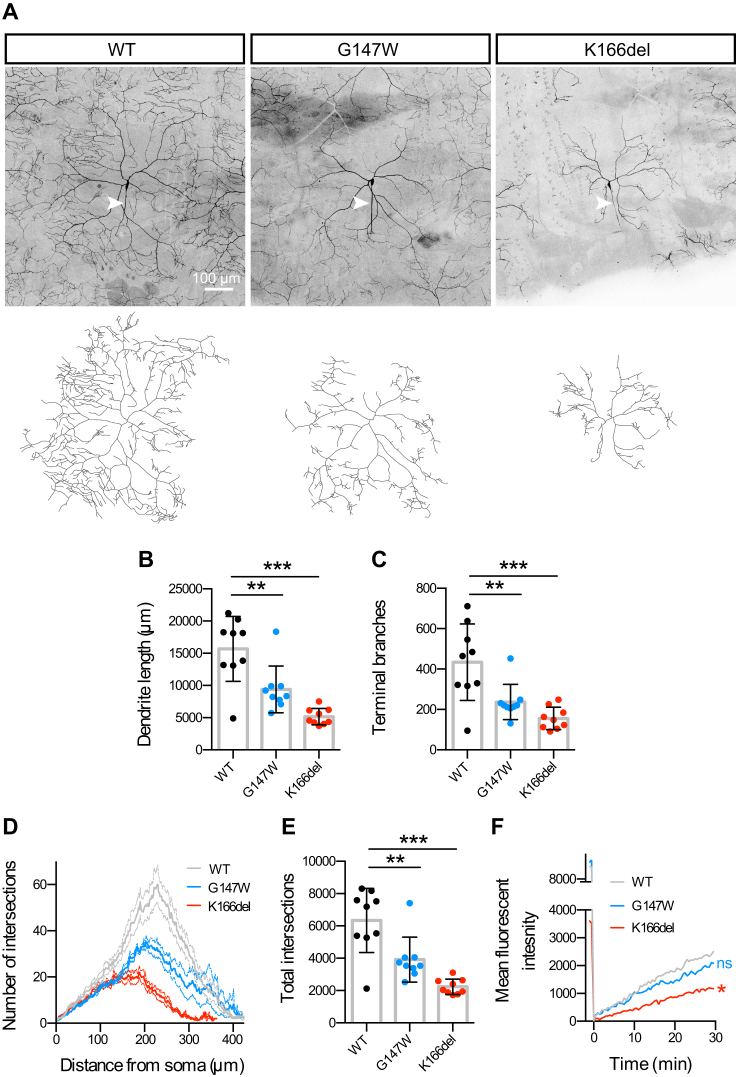
Membrin Mutations Cause Dendritic Growth Deficits (A) Maximum intensity projections of ddaC abdominal segment 5 neurons genetically labeled with *ppk* > CD4::tdGFP in Mem-WT/-G147W/-K166del. Respective tracings of the dendritic arbors are shown below. Arrowheads indicate axons. (B) Total dendritic length extracted from tracings as shown in (A). 9 A5 ddaC neurons per genotype were traced and analyzed in (B)–(E). (C) Number of terminal branches of ddaC A5 neurons. (D) Number of intersections of dendritic tracings with concentric circles with 2 pixel/circle increasing radii. Mean ± SEM are shown. (E) Total intersection of Sholl analysis as shown in (D). (F) CD4::tdGFP in large segments of primary ddaC A5 dendrites adjacent to the soma were photobleached with a 50 μm^2^ region of interest and fluorescence recovery quantified 25 μm from the soma proximal bleach margin. Means of n = 9, 8, and 9 ddaC neurons for Mem-WT/-G147W/-K166del are shown. Asterisks and ns indicate endpoint comparison after 29.5-min recovery. Replicate values, mean, and SD are shown unless otherwise stated. ^∗^p < 0.05; ^∗∗^p < 0.01; ^∗∗∗^p < 0.001; ns, not significant (p > 0.05); one-way ANOVA with Dunnett’s multiple comparison test.

However, whereas Q71L-Arf1 and truncated Sar1 result in a substantial or complete block of anterograde trafficking (Dascher and Balch, 1994, Ye et al., 2007), the above liposome fusion assays and *Drosophila GOSR2*-PME models indicate that PME-causing mutations in Membrin involve a partial loss of function (Figures 1 and 3). Thus, we sought to test whether a partial decrease in ER-to-Golgi trafficking could impair dendritic growth. To do so, we genetically labeled ddaC sensory neurons within the larval body-wall with a membrane-tagged (and thus secretory-pathway-dependent) fluorophore (CD4::tdGFP) under control of the *ppk* promoter (Han et al., 2011). These neurons have highly complex, tiled dendritic arbors that branch in 2D and are unambiguously polarized into a single axon and multiple dendrites (Figure 4A, arrowhead indicates the axon). We imaged the same identifiable ddaC neuron in abdominal segment 5 of Mem-WT, Mem-G147W, and Mem-K166del L3 larvae (Figure 4A). Strikingly, both Mem-G147W and Mem-K166del larvae exhibited profound reductions in dendritic length and the number of terminal dendritic branches relative to Mem-WT (Figures 4B and 4C). Sholl analysis further revealed reduced elaboration of dendritic arbors and a significant reduction in dendritic intersections in Mem-G147W and Mem-K166del (Figures 4D and 4E). Using fluorescence recovery after photo-bleaching (FRAP), we tested whether trafficking of the CD4::tdGFP cargo was altered in Mem-G147W and Mem-K166del dendrites. Mem-K166del larvae exhibited a clear reduction in CD4::tdGFP FRAP and a similar albeit non-significant trend was seen for Mem-G147W (Figure 4F). Thus, dendritic growth and cargo trafficking within dendrites are impacted to a greater degree in Mem-K166del than Mem-G147W, consistent with the more severe SNARE defect of the orthologous D196del Bos1 mutation in liposome fusion assays (Figure 1).

### Reduced Cargo Trafficking in Membrin Mutant Axons

**Figure 5:**
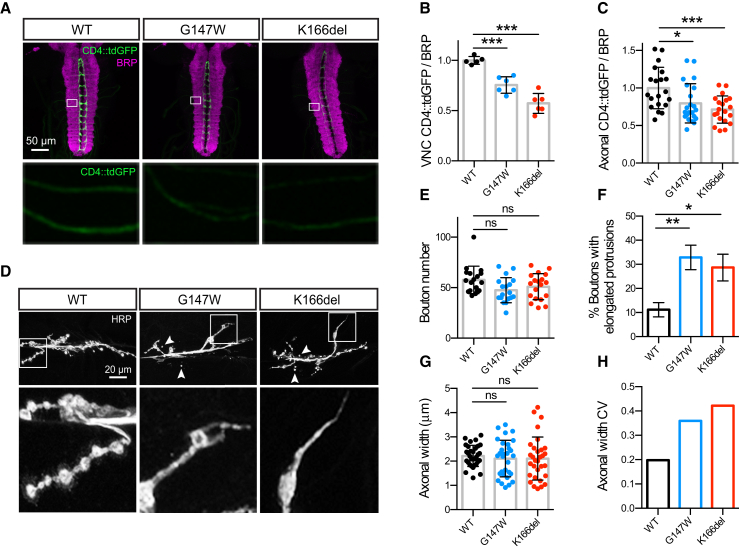
Membrin Mutations Alter Presynaptic Morphology and Axonal Stereotypy (A) Top: confocal z-stacks showing projections from *ppk*-positive sensory neurons labeled with membrane-tagged CD4::tdGFP innervating the ventral nerve cord (VNC) of L3 larvae. Synaptic neuropil of the VNC is labeled with anti-BRP. Below: magnified regions of segmental nerves. (B and C) Quantification of CD4::tdGFP fluorescence in the VNC neuropil (B) or in segmental nerves (C) normalized to BRP and expressed relative to Mem-WT. n = 5, 5, and 6 (B) and n = 20, 22, and 20 (C) for Mem-WT/-G147W/-K166del. (D) Top: example confocal z-stacks of HRP-labeled motor neurons innervating muscle 6/7, segment 3 of L3 larvae. Arrowheads point to small, isolated boutons that appear unattached to the axon. Below: magnified terminal boutons. In contrast to the rounded morphology in Mem-WT larvae, terminal boutons in Mem-G147W and Mem-K166del larvae often exhibit elongated protrusions. Further examples are depicted in Figure S6C. (E) Average number of boutons (type 1b and 1s) at muscle 6/7, segment 3. n = 18, 18, and 19 for Mem-WT/-G147W/-K166del. (F) Percentage of terminal boutons with elongated protrusions. Mean and SEM are shown. n = 32, 31, and 31 for Mem-WT/-G147W/-K166del. (G) Maximal axonal width of motor neurons innervating muscle 6/7, segment 3. n = 32, 30, and 31 for Mem-WT/-G147W/-K166del. (H) Coefficient of variation (calculated as SD/mean) in axonal width for each genotype. Replicate values, mean and SD are shown unless otherwise stated. ^∗^p < 0.05; ^∗∗^p < 0.01; ^∗∗∗^p < 0.001; ns, not significant (p > 0.05); one-way ANOVA with Dunnett’s multiple comparison test (B and C) or Kruskal-Wallis test with Dunn’s post hoc test (E–G).

While both growing dendrites and axons require abundant membrane addition (Aridor and Fish, 2009), Ye et al. (2007) suggested that axonal growth can be privileged in the face of ER-to-Golgi trafficking deficits. We thus examined axonal growth in *GOSR2*-PME *Drosophila* models. We detected axonal CD4::tdGFP signal in each ventral nerve cord (VNC) segmental nerve (Figure 5A; for saturated images, see Figure S4A), suggesting that at least one axon derived from the three *ppk*-positive neurons per hemisegment (ddaC, v’ada, and vdaB) reached its comparably distant target (Grueber et al., 2002). Compared with the reduced elaboration of ddaC dendrites (Figure 4), this is consistent with dendritic growth being more severely impaired than axonal growth by *GOSR2*-PME mutations. Nevertheless, steady-state CD4::tdGFP levels were significantly reduced in both the VNC and individual segmental nerves of Mem-G147W and Mem-K166del (Figures 5A–5C). Thus, a secretory pathway deficit is clearly present in distal axons and/or synapses of *membrin* mutant *Drosophila*. Using the large and experimentally accessible neuromuscular junction (NMJ) of L3 larvae, we next tested whether Membrin mutations also altered trafficking of endogenous synaptic cargos. We found robust reductions in the steady state levels of the synaptic vesicle protein cysteine string protein (CSP) in Mem-G147W and Mem-K166del synapses, while several other synaptic cargos were unaltered compared to Mem-WT (Figures S4B–S4H). These results provide proof of principle that Membrin mutations can affect the abundance of specific synaptic proteins. The different effects upon individual synaptic components at steady state may reflect varying trafficking demands of synaptic proteins due to differences in synaptic turnover.

### Unimpaired Secretory Trafficking in G144W Membrin Mutant Fibroblasts

*GOSR2*-PME patients exhibit a restrictive neurological phenotype despite the ubiquitous expression of Membrin. We wondered whether the neuronal secretory pathway deficit observed in *GOSR2*-PME model axons might be the consequence of the unique geometry of neurons and thus not be applicable to a non-neuronal cell. To test this, we performed Golgi trafficking assays in a non-neuronal cell-type (primary skin fibroblasts) derived from a G144W *GOSR2*-PME patient and healthy controls. In this cell-type, we overexpressed human growth hormone (hGH) fused to four FM domains and a Halo tag. The FM domains self-aggregate in the absence of a solubilizing drug and thus do not allow ER exit of hGH (Figure S5A, first column) (Rivera et al., 2000). Addition of D/D solubilizer disaggregates the FM domains and allows the cargo to enter the secretory pathway. By 10 min after solubilization, we detected significant colocalization of tagged hGH with the *cis*-Golgi marker GM130, which decreased by 20 and 30 min as the cargo exited this compartment (Figures S5A and S5B). Remarkably, trafficking kinetics in G144W mutant Membrin fibroblasts were almost indistinguishable from controls (Figures S5A and S5B). This finding supports the concept that cellular effects of the partial loss-of-function G144W Membrin mutation are unmasked only under large secretory pathway requirements, suggesting why only the nervous system with its high trafficking demands is symptomatically affected in *GOSR2*-PME.

### Presynaptic Morphological Defects in *GOSR2*-PME Model Neurons

Because we could detect secretory pathway defects in distal axons and synapses of *GOSR2*-PME *Drosophila*, we next investigated whether synaptic integrity might be altered due to the *GOSR2*-PME mutations. We thus examined presynaptic morphology at the L3 larval NMJ by labeling neuronal membranes with an anti-horseradish peroxidase (HRP) antibody. Motor neurons of Mem-WT, Mem-G147W, and Mem-K166del flies successfully formed synapses, and Mem-G147W and Mem-K166del synapses did not exhibit significant reductions in bouton number or size relative to Mem-WT (Figures 5D, 5E, and S6A–S6C). However, we observed two clear morphological abnormalities in Mem-G147W and Mem-K166del synapses. First, terminal synaptic boutons often exhibited axonal protrusions lacking normal rounded boutons, which were less common and shorter in Mem-WT synapses (Figures 5D, 5F, S6C, and S6D). Second, we observed small boutons that were disconnected from the main axonal branch (Figure 5D, arrowheads). In addition, analysis of axonal diameter revealed a significant increase in the variability of the maximal axonal diameter in Mem-G147W and Mem-K166del NMJs (Figure 5G), as measured by the coefficient of variation (Figure 5H) and F-test (Mem-G147W versus Mem-WT, p = 0.0035; Mem-K166del versus Mem-WT, p = 0.0002). Thus, partial reductions in secretory pathway trafficking result in multifaceted abnormalities in motor neuron synapse development and impact the stereotypy of terminal axon morphology.

### Membrin Mutations Result in Synaptic Retraction and Cytoskeletal Fragmentation

**Figure 6:**
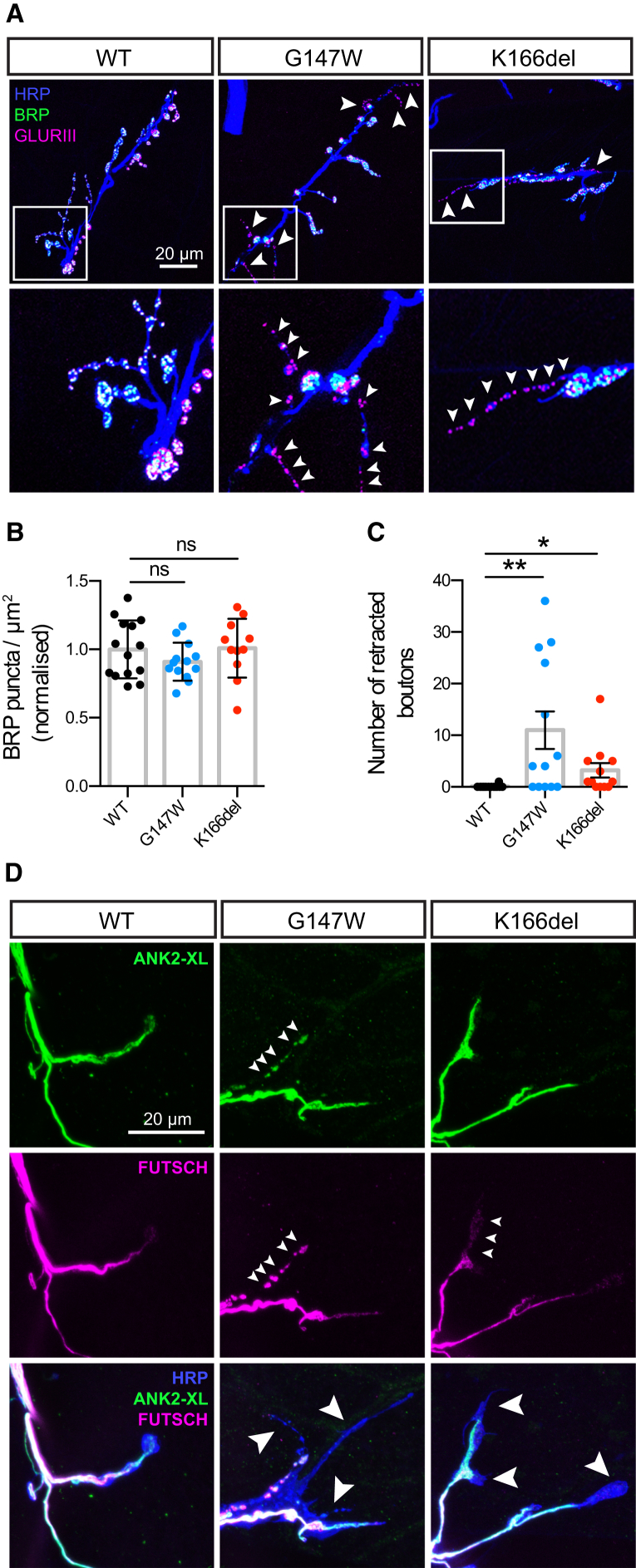
Synaptic Retraction and Presynaptic Cytoskeletal Fragmentation in *membrin* Mutants (A) Top: Maximum intensity z projection of confocal stacks showing pre- and postsynaptic apposition between BRP-labeled active zones and postsynaptic GLURIII glutamate receptors. Arrowheads denote regions where glutamate receptors lack their presynaptic active zone counterparts. Below: magnified view of regions exhibiting loss of BRP-labeled active zones in Mem-G147W and Mem-K166del synapses. (B) Normalized density of BRP puncta per NMJ area. n = 14, 13, and 11 for Mem-WT/-G147W/-K166del. Replicate values, mean, and SD are shown. (C) Average number of synaptic boutons where BRP fails to oppose GLURIII. n = 14, 13, and 12 for Mem-WT/-G147W/-K166del. Replicate values, mean, and SEM are shown. (D) Confocal z-stack maximum intensity projections illustrating localization of Ankyrin-2-XL (ANK2-XL) and Futsch. Small arrowheads point to synaptic domains containing either fragmented Futsch and ANK2-XL or reduced amounts of Futsch. Large arrowheads point to synaptic boutons and elongated protrusion apparently lacking both Futsch and ANK2-XL. ^∗^p < 0.05; ^∗∗^p < 0.01; ns, not significant (p > 0.05); Kruskal-Wallis test with Dunn’s post hoc test.

To examine the effects of Membrin mutations on synaptic structure in more detail, we co-stained Mem-WT, Mem-G147W, and Mem-K166del synapses with antibodies against the presynaptic active zone marker Bruchpilot (BRP) and postsynaptic GLURIII glutamate receptors (Figure 6A). BRP localized to Mem-G147W and Mem-K166del NMJs in amounts comparable to Mem-WT (Figure 6B). However, dual pre- and postsynaptic labeling revealed pronounced strings of small presynaptic boutons in Mem-G147W and Mem-K166del synapses in which GLURIII was no longer opposed by BRP (Figures 6A and 6C). This disruption of transsynaptic organization is indicative of presynaptic retraction, where synaptic connections initially form but fail to be maintained throughout development (Eaton et al., 2002, Pielage et al., 2008).

Synaptic retraction can be induced by mutations in cytoskeletal proteins (Koch et al., 2008, Pielage et al., 2008, Pielage et al., 2011, Pielage et al., 2005). Long protrusions lacking specialized boutons and alterations in axonal diameter further suggested cytoskeletal defects in Mem-G147W and Mem-K166del synapses (Pielage et al., 2011, Stephan et al., 2015). Hence, we examined the localization of two presynaptic cytoskeletal proteins: Futsch (a microtubule-binding protein) and Ankyrin-2-XL (ANK2-XL) (Koch et al., 2008, Roos et al., 2000). In Mem-WT synapses, both Futsch and ANK2-XL were co-localized in central and distal axons and invaded terminal boutons (Figure 6D) (Stephan et al., 2015). Strikingly, in both terminal boutons and elongated protrusions of Mem-G147W and Mem-K166del synapses, we observed either fragmentation of the normally continuous Futsch- and ANK2-XL-labeled cytoskeleton or an absence of one or both proteins (Figure 6D). Thus, secretory defects due to Membrin mutations reduce the local integrity of the presynaptic cytoskeleton.

### Physiological Abnormalities at Membrin Mutant Synapses

**Figure 7:**
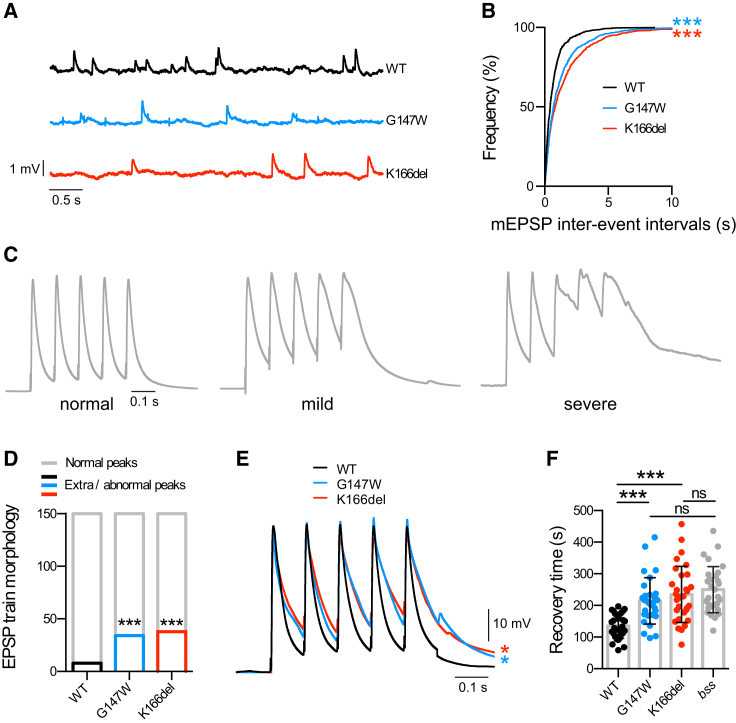
Physiological Abnormalities at *membrin* Mutant NMJs (A) Representative traces of miniature excitatory postsynaptic potentials (mEPSPs) recorded from Mem-WT/-G147W/-K166del L3 larval muscle 6 abdominal segments 2–4. (B) Cumulative frequency plot of mEPSP intervals. 800 events per genotype from 8 animals each are shown. (C) Illustrative traces depicting mild to severe EPSP waveform distortion following 5 stimuli at 10 Hz. Traces are normalized to the peak amplitude. (D) Analysis of total number of abnormal events as a result of 10 Hz stimulation. 15 events were analyzed from each recording from 10 animals per genotype. (E) Overlay of averaged 10 Hz EPSP trains illustrating a significantly larger mean area under the curve in Mem-G147W and Mem-K166del compared to Mem-WT larvae (n = 10). (F) Mem-G147W and Mem-K166del larvae displayed longer recovery times after CNS electroshock compared to Mem-WT, indicative of increased seizure severity of the *GOSR2*-PME models. Mem-G147W and Mem-K166del recovery times were not significantly different compared to the *Drosophila* seizure model *bang-senseless* (*bss*). Replicate values, mean and SD are shown. n = 30 for Mem-WT/-G147W/-K166del/*bss*. ^∗^p < 0.05; ^∗∗∗^p < 0.001; Kolmogorov-Smirnov test and Bonferroni correction (B), Fisher’s exact test and Bonferroni correction (D), one-way ANOVA with Dunnett’s multiple comparison test (E), and Kruskal-Wallis test with Dunn’s post hoc test (F).

We next asked whether Membrin mutations altered spontaneous or evoked neurotransmitter release at the L3 larval NMJ. We detected a clear reduction in the frequency of spontaneous miniature excitatory postsynaptic potentials (mEPSPs) in Mem-G147W and Mem-K166del flies (Figures 7A and 7B), while the amplitude and time course of mEPSPs were comparable between WT and Membrin mutants (Figures S7A and S7B). No effect of Mem-G147W and Mem-K166del mutations on the amplitude of single postsynaptic evoked EPSPs was observed (Figure S7C). However, we often observed grossly deformed trains of EPSPs in both Mem-G147W and Mem-K166del following 5 consecutive stimuli at 10 Hz, where between one and all five EPSPs exhibited broader waveforms with multiple peaks and occasional merging of EPSPs (Figure 7C). Significantly more EPSP trains were abnormal in both mutants compared to Mem-WT: 5% of EPSP trains in Mem-WT were scored abnormal by a blinded observer, compared to ∼25% in Mem-G147W and 22% in Mem-K166del (Figure 7D). In addition, the area under the EPSP train was robustly increased in Mem-G147W and Mem-K166del compared to Mem-WT (Mem-WT: 9013 mVs ± 850.5 (mean ± SEM); Mem-G147W: 15,226 ± 1,706; Mem-K166del: 15,992 ± 2,298) (Figure 7E).

Finally, given that *GOSR2*-PME is an epilepsy syndrome, we tested whether such neuronal hyperactivity and the dendritic/synaptic morphological abnormalities resulted in seizure-like neuronal activity. To do so, we electrically induced seizures in Mem-WT and *GOSR2*-PME model L3 larvae and measured the recovery time required to resume normal locomotor behavior (Giachello and Baines, 2015). Remarkably, Mem-G147W and Mem-K166del displayed a significant increase in duration of seizure-like activity when compared to Mem-WT (Figure 7F). The effect sizes of our *GOSR2*-PME models were comparable to the widely studied *bang-senseless Drosophila* seizure model (Figure 7F) (Parker et al., 2011). Thus, our findings indicate that pathogenic *membrin* mutations cause not only dendritic and synaptic morphological abnormalities but also altered synaptic function, which collectively impact the nervous system in a way to give rise to locomotor defects and hyperexcitability.

## Discussion

To date, how mutations in Membrin, a ubiquitous and essential Golgi SNARE protein, manifest as a disorder restricted to the nervous system has been unclear. Here, we demonstrate that PME-causing Membrin mutations partially reduce SNARE activity yet still result in profound dendritic growth deficits in *Drosophila* models. Furthermore, Membrin mutations cause synaptic disassembly and altered neurotransmission, establishing a close dependence of synaptic stability and physiology upon precisely tuned secretory trafficking.

Our findings reinforce a previous *Drosophila* screen identifying the ER-to-Golgi trafficking proteins Sar1, Sec23, and Rab1 to be required for dendrite growth (Ye et al., 2007) and provide an instance that highlights the potential clinical relevance of this pathway (Jan and Jan, 2010). Interestingly, mutations in Sec23A, Sec23B, Sec24D, and Sar1b present in humans with largely non-neuronal clinical phenotypes of cranio-lenticulo-sutural dysplasia, congenital dyserythropoietic anemia, a syndromic form of osteogenesis imperfecta and lipid absorption disorders (Boyadjiev et al., 2006, Garbes et al., 2015, Jones et al., 2003, Schwarz et al., 2009). This appears to be a consequence of tissue-specific differential utilization of the two available isoforms of Sec23 and Sar1 and special demands upon the COPII coat due to the large size of procollagen and chylomicrons (Garbes et al., 2015, Fromme et al., 2007, Jones et al., 2003).

Our results suggest that subtle defects in the secretory pathway can be highly relevant for neuronal growth while not robustly affecting a non-neuronal cell type such as a fibroblast, supporting the postulate that due to the unique plasma membrane demands of a growing neuron, minor disruption of the secretory pathway can disrupt the nervous system while falling below a critical threshold in other organs (Pfenninger, 2009). While we cannot fully rule out a contribution to *GOSR2*-PME symptoms from non-neuronal cells, we hypothesize that this is the likeliest explanation for why *GOSR2*-PME symptoms selectively involve nervous system dysfunction. Furthermore, the observed dendritic growth deficits may explain one hallmark of this disorder—lack of coordination—since reduced membrane transport may most severely impact neurons with highly elaborate dendritic arbors such as cerebellar Purkinje cells (Ramón y Cajal, 1906). Such impairment would likely give rise to ataxia, as Purkinje cells are critically important for motor coordination (Kasumu and Bezprozvanny, 2012). Interestingly, cerebellar defects have been suggested to be involved in the pathogenesis of cortical myoclonus (Ganos et al., 2014).

Our study extends previous findings by showing that early secretory pathway changes can also impact presynaptic morphology and physiology as well as dendritogenesis (Ye et al., 2007). We found that larval NMJs of Mem-G147W and Mem-K166del exhibit synaptic retraction, abnormal elongated protrusions lacking synaptic specializations, reduced spontaneous neurotransmitter release, and malformed EPSPs.

Membrin acts as a gatekeeper at the *cis*-Golgi and likely determines the trafficking rates of a plethora of synaptic and axonal proteins. Thus, we speculate that the observed synaptic changes arise from a complex interaction of trafficking delays or steady-state reductions of many cargos. Nevertheless, we identify several molecular correlates of the above structural changes. These include the loss or fragmentation of the cytoskeletal proteins ANK2-XL (an Ankyrin-2 isoform) and Futsch, particularly in presynaptic boutons with elongated protrusions. The axonal and synaptic cytoskeleton contains several interlinked constituents, including a microtubule core, actin filaments, and a submembranous mesh of Ankyrin and Spectrin (Goellner and Aberle, 2012). These components regulate an array of neurodevelopmental and physiological parameters, including synaptic growth, morphology, and stability; axonal caliber; and ion channel localization (Goellner and Aberle, 2012, Stephan et al., 2015). Futsch is the *Drosophila* homolog of the mammalian microtubule-binding protein MAP1B (Roos et al., 2000), and the absence or fragmentation of Futsch in Mem-G147W and Mem-K166del synapses implies similar alterations in microtubule stability. Given the synaptic retraction observed in Mem-G147W and Mem-K166del NMJs, it is interesting to note that destabilization of microtubules is an early event during naturally occurring synapse loss at the mammalian NMJ (Bishop et al., 2004, Brill et al., 2016). Ankyrin-2 isoforms delineate *Drosophila* synaptic termini into rounded boutons separated by thin inter-bouton domains (Koch et al., 2008, Pielage et al., 2008). Since Mem-G147W and Mem-K166del boutons often exhibit elongated protrusions reminiscent of extended inter-bouton domains, we speculate direct links among local destabilization of the synaptic cytoskeleton, synaptic retraction, and the presence of elongated protrusions in Mem-G147W and Mem-K166del synapses. Interestingly, EMG evidence of motoneuron denervation in *GOSR2*-PME patients has been reported, and the typical absence of deep-tendon reflexes in this disorder might be a consequence of analogous changes (van Egmond et al., 2014).

Membrin mutations not only affect synaptic structure but also disrupt evoked and spontaneous neurotransmitter release. Again, the underlying mechanisms are likely to represent a complex, cumulative process caused by insufficient trafficking of diverse ion channels and vesicle release proteins. Increased variability of axonal diameters in Mem-G147W and Mem-K166del may further alter action potential propagation velocities across different axons innervating the same muscle, leading to desynchronized depolarizing currents.

Our work raises the possibility that other PME subtypes might share cellular pathways and/or neural circuits with *GOSR2*-PME. For instance, mutations in *PRICKLE1* cause PME in humans and seizures in flies (Bassuk et al., 2008, Tao et al., 2011), and *PRICKLE1* has been linked to neurite growth and axonal trafficking (Ehaideb et al., 2014, Liu et al., 2013). PME-linked mutations in the potassium channel gene *KCNC1* are thought to mainly impair fast-spiking neurons (Muona et al., 2015). Such a preferential defect in high-frequency firing neurons is also conceivable in *GOSR2*-PME, where EPSP abnormalities are more pronounced under repetitive stimulation.

In summary, by elucidating the pathophysiology of *GOSR2*-PME, we identify a critical role for Membrin in promoting synaptic integrity, highlight stringent requirements of dendritic growth on the secretory pathway, and define how mutations in an essential gene can selectively disrupt nervous system function.

## Experimental Procedures

### Molecular Biology and Bioinformatics

Human *GOSR2* CDS as well as *Drosophila melanogaster membrin* CDS with and without the G144W/G147W and K164del/K166del mutations preceded by 5′ FLAG-tag coding sequence were custom synthesized by GeneArt (Thermo Fisher Scientific) and subsequently cloned via NotI and KpnI (NEB) into pUASTattB, giving rise to pUASTattB_FLAG::*GOSR2*[WT]/pUASTattB_FLAG::*membrin*[WT], pUASTattB_FLAG::*GOSR2*[G144W]/pUASTattB_FLAG::*membrin*[G147W], and pUASTattB_FLAG::*GOSR2*[K164del]/pUASTattB_FLAG::*membrin*[K166del]. See Supplemental Experimental Procedures for further details.

### Liposome Fusion Assays

The recombinant yeast Golgi SNARE proteins were expressed and purified in *E. coli* BL21 (DE3) cells as described previously (Parlati et al., 2000). Purified SNARE proteins were reconstituted into lipid vesicles using the detergent (1% n-octyl-β-D-glucopyranoside) dilution and dialysis method (Weber et al., 1998). See Supplemental Experimental Procedures for further details.

### Cell Culture and Transfections

Primary skin derived fibroblasts from the first described *GOSR2*-PME patient were kindly shared by Mark Corbett (Corbett et al., 2011). As controls, we used fibroblasts from healthy individuals of either the same sex and similar age or the opposite sex and divergent age (control 1, 23-year-old female; control 2, 60-year-old male [at time of biopsy]). See Supplemental Experimental Procedures for further details.

### Fibroblast Imaging

For immunofluorescence studies, cells were seeded on #1.5 glass coverslips, fixed with 4% paraformaldehyde (PFA), and permeabilized in PBS containing Triton X-100 and NP40. After primary and secondary antibody incubation steps, coverslips were mounted in SlowFade Gold Antifade (Thermo Fisher Scientific). See Supplemental Experimental Procedures for further details.

### Western Blot

Cells were lysed in 20 mM HEPES (pH 7.5), 100 mM KCl, 5% glycerol, 10 mM EDTA, and 1% Triton X-100 supplemented with phosphatase and proteinase inhibitors (PhosSTOP/cOmplete, Roche). Total protein content was quantified with the Pierce 660 nm assay (Thermo Fisher Scientific) and equal amounts loaded into each lane. Proteins were separated on a 4%–10% Bis-Tris polyacrylamide gel (Thermo Fisher Scientific) and transferred onto polyvinylidene fluoride (PVDF) membranes (EMD Millipore). See Supplemental Experimental Procedures for further details.

### *Drosophila* Genetics and Phenotyping

*membrin*^1,524^ flies were previously generated in an ethyl methanesulfonate (EMS) screen and kindly shared by Mark Krasnow (Ghabrial et al., 2011). This strain harbors a premature stop codon upstream of the *membrin* SNARE domain encoding sequence and therefore represents a null allele. To control for potential genetic background effects, we outcrossed *membrin*^1,524^ for five generations into an isogenic iso31 background by following an AccI (NEB) restriction site that is introduced by the nonsense mutation. *daughterless*-Gal4 (#55850), UAS-*GCaMP6m* (#42748), and *nsyb*-Gal4 (#51635) flies were obtained from the Bloomington Stock Center, and the *membrin* RNAi transgene was obtained from the Vienna *Drosophila* Resource Center (VDRC) (GD 44535) (Dietzl et al., 2007). These transgenes and alleles were backcrossed for 5 generations into an isogenic iso31 background. Backcrossed *elav*-Gal4 flies were a kind gift from Kyunghee Koh. Flies were reared on a standard cornmeal-molasses-yeast medium at 25°C in a 12-hr light/dark cycle. See Supplemental Experimental Procedures for further details.

### Dendritic Analysis

The highly elaborate ddaC neurons in abdominal segment 5 of L3 larvae were used throughout (Grueber et al., 2002). For morphological analysis, larvae were heat-killed and mounted under a #1.5 glass coverslip. z stacks of ddaC neurons were obtained with Zeiss confocal LSM710 microscopes with a N-Achroplan 10× 0.25 numerical aperture (NA) objective to capture the entire arbor. See Supplemental Experimental Proceduresfor further details.

### Immunohistochemistry of Larval Neuromuscular Junctions and Brains

When examining synaptic development at the larval NMJ, synapses innervating muscle 6/7 of segment 3 were imaged on a Zeiss confocal LSM710 with either a Plan-Apochromat 20× 0.8 NA or a Plan-Apochromat 63× 1.4 NA oil-immersion objective. See Supplemental Experimental Procedures for further details.

### NMJ Electrophysiology and Larval Seizure Assay

Wandering L3 larvae were dissected in ice-cold, Ca^2+^-free HL3-like solution (70 mM NaCl, 5 mM KCl, 10 mM NaHCO_3_, 115 mM sucrose, 5 mM trehalose, 5 mM HEPES, and 10 mM MgCl_2_). Motor nerves were severed just below the VNC, and the brain was removed. CaCl_2_ (1 mM) was added to the bath solution for intracellular recording from muscle 6 of abdominal segments 2–4. Sharp microelectrodes (thick-walled borosilicate glass capillaries, pulled on a Sutter Flaming/Brown P-97 micropipette puller) were filled with 3 M KCl and had resistances of 20–30 MΩ. For recording of stimulus evoked excitatory postsynaptic potentials (EPSPs), severed nerves were drawn into a thin-walled glass-stimulating pipette and stimulated with square-wave voltage pulses (0.1 ms, 10 V, A-M Systems Model 2100 Isolated Pulse Simulator). EPSPs and spontaneously occurring mEPSPs were recorded at a controlled room temperature of 22°C –25°C with a Geneclamp 500 amplifier (Axon Instruments) and were further amplified with an LHBF-48x amplifier (NPI Electronic). Wandering third-instar larvae were electroshocked as previously described (Giachello and Baines, 2015). See Supplemental Experimental Procedures for further details.

### Statistical Comparisons

Statistical analyses were performed using GraphPad Prism. Tests used to compare control and experimental populations are detailed in the figure legends.

## Author Contributions

Conceptualization, R.P., S.S.K., J.E.R., and J.E.C.J.; Methodology, R.P., N.T.M., S.S.K., and J.E.C.J.; Formal Analysis, R.P., S.A.L., N.T.M., C.N.G.G., J.E.C.J.; Investigation, R.P., S.A.L., N.T.M., C.N.G.G., N.P., J.E.C.J.; Resources, H.H., D.M.K., R.A.B., M.M.U., J.J.L.H., J.E.R., and J.E.C.J.; Writing – Original Draft, R.P. and J.E.C.J.; Writing – Review & Editing, R.P., S.A.L., N.T.M., C.N.G.G., D.M.K., R.A.B., M.M.U., S.S.K., J.J.L.H., J.E.C.J.; Visualization, R.P. and J.E.C.J.; Supervision, H.H., D.M.K., R.A.B., M.M.U., S.S.K., J.J.L.H., J.E.R., and J.E.C.J.; Project Administration, R.P. and J.E.C.J.; Funding Acquisition, R.P., H.H., D.M.K., R.A.B., M.M.U., J.J.L.H., J.E.R., and J.E.C.J.

## Supplemental Information

Document S1. Supplemental Experimental Procedures and Figures S1–S7

Document S2. Article plus Supplemental Information
