# Supplementary material for: Mutations in Membrin/GOSR2 Reveal Stringent Secretory Pathway Demands of Dendritic Growth and Synaptic Integrity
Source: Cell Rep. 2017 Oct 3;21(1):97–109. doi: 10.1016/j.celrep.2017.09.004 (PMC5640804; doi:10.1016/j.celrep.2017.09.004)
Supplement: Document S1. Supplemental Experimental Procedures and Figures S1–S7 [file mmc1.pdf]

Cell Reports, Volume *21*

Supplemental Information

Mutations in Membrin/*GOSR2* Reveal Stringent Secretory Pathway Demands of Dendritic Growth and Synaptic Integrity

Roman Praschberger, Simon A. Lowe, Nancy T. Malintan, Carlo N.G. Giachello, Nian Patel, Henry Houlden, Dimitri M. Kullmann, Richard A. Baines, Maria M. Usowicz, Shyam S. Krishnakumar, James J.L. Hodge, James E. Rothman, and James E.C. Jepson

A

t-SNARE	Sed5	Sec22	Bos1
WT	1.0x	1.0±0.52x	1.2±0.39x
G176W	1.0x	1.0±0.42x	1.3±0.36x
D196del	1.0x	0.9±0.42x	1.0±0.51x

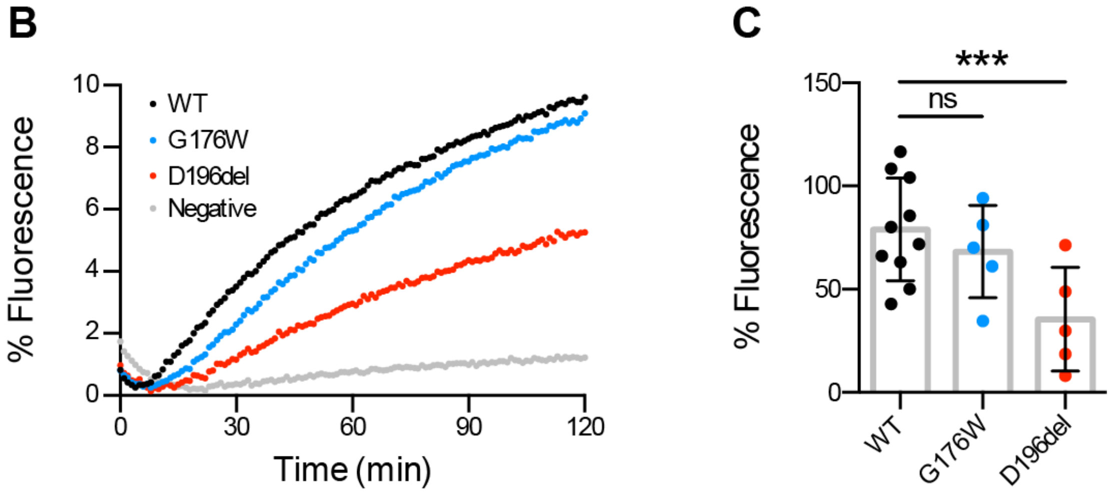

Figure S1. Liposome t-SNARE stoichiometry and liposome fusion assay after 4°C preincubation, Related to Figure 1

(A) Stoichiometry of the pre-assembled t-SNARE complexes containing either wild type (WT) or G176W/D196del mutant Bos1 determined by densitometry.

(B) Example traces of experiment as in Figure 1C with the modification that prior to measuring the fusion kinetics at 37°C the reactions were pre-incubated overnight at 4°C.

(C) Endpoint (120 min) quantification of experiment as described in (B), normalized to WT. n = 10, 5, 5 for WT, G176W, D196del. Replicate values, mean and SD are shown. *** represents $p < 0.001$, ns = not significant ($p > 0.05$); ANOVA with Dunnett's multiple comparison test.

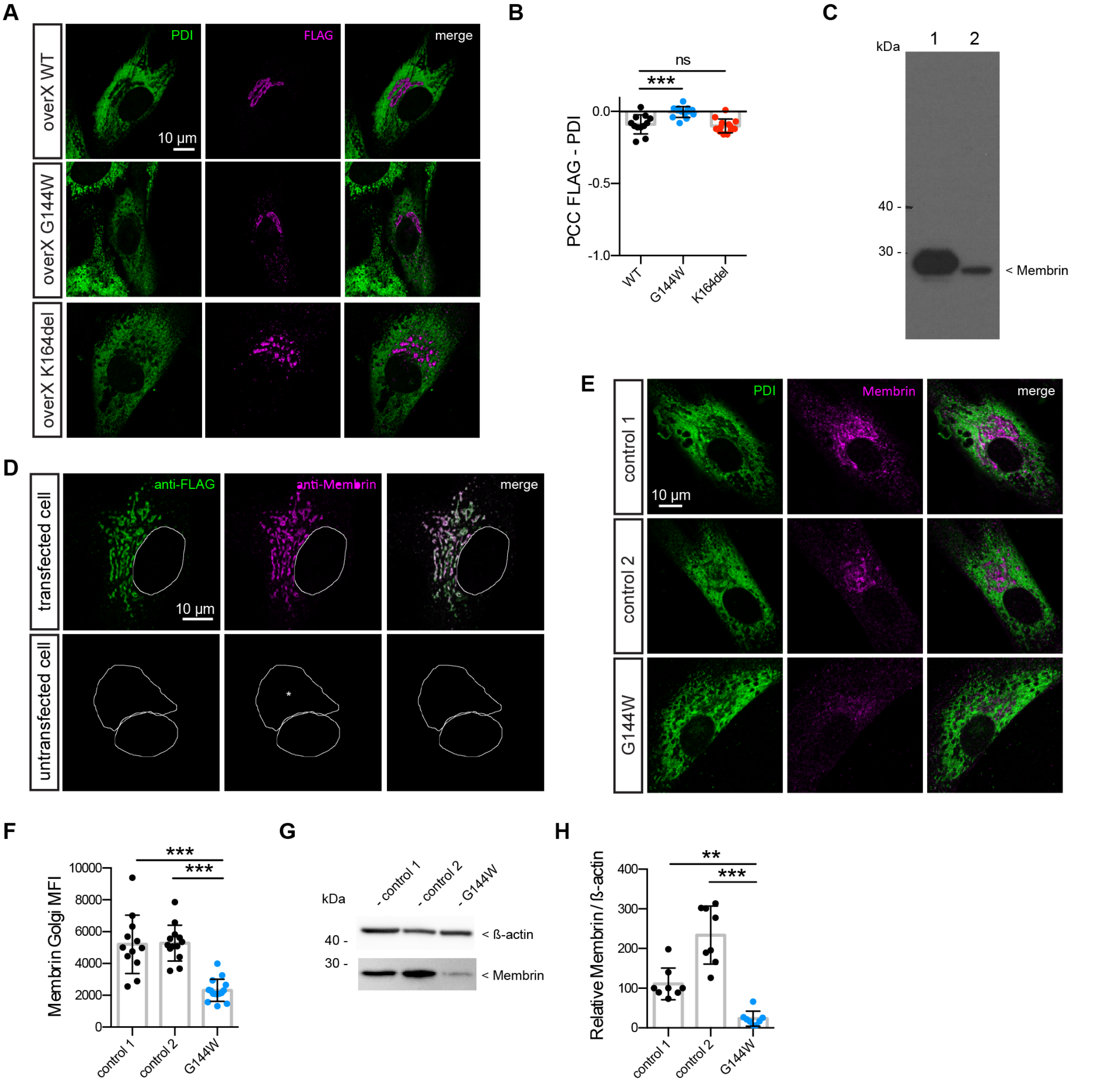

Figure S2. Membrin subcellular localization, antibody validation and quantification, Related to Figure 2
(A) FLAG-tagged WT and G144W/K164del mutant Membrin were overexpressed in control fibroblasts and co-stained for FLAG and the ER resident protein PDI. Example confocal slices are shown for each overexpressed construct.
(B) Pearson's correlation coefficients (PCC) between FLAG and PDI signal of the experiment described in (A) are shown. n = 12, 13, 13 for WT, G144W, K164del.
(C) The Membrin antibody used in Figure 2 exhibits high specificity using western blotting. Only one band at the expected molecular weight of approximately 27 kDa is apparent. Equal total protein amounts of HEK293T cell lysate (lane 2) or HEK293T cells overexpressing WT FLAG::Membrin (lane 1) were probed.
(D) The Membrin antibody used in Figure 2 with the same immuno-fluorescence protocol recognizes WT FLAG::Membrin overexpressed in control fibroblasts. Top row shows an overexpressing cell, where Membrin and FLAG signal co-localize. At the identical settings endogenous Membrin cannot be observed in an untransfected cell. Boundaries of nuclei and Golgi region (*), as seen by DIC imaging and Membrin signal at higher contrast settings respectively, are outlined.
(E) Example confocal slices of control and Membrin G144W mutant fibroblasts co-stained for endogenous Membrin and PDI.
(F) Membrin mean fluorescent intensity (MFI) in the Golgi region as demarcated by GPP130. Quantification of experiment as shown in Figure 2D. n = 12, 13, 15 for control 1, control 2, G144W.
(G) Western blot of lysates from control and G144W Membrin mutant fibroblasts.
(H) Quantification of Membrin protein from western blots as described in (G), normalized to control 1. n = 8.

Replicate values, mean and SD are shown. **, *** represent $p < 0.01$, 0.001, ns = not significant ($p > 0.05$); ANOVA with Dunnett's multiple comparison test.

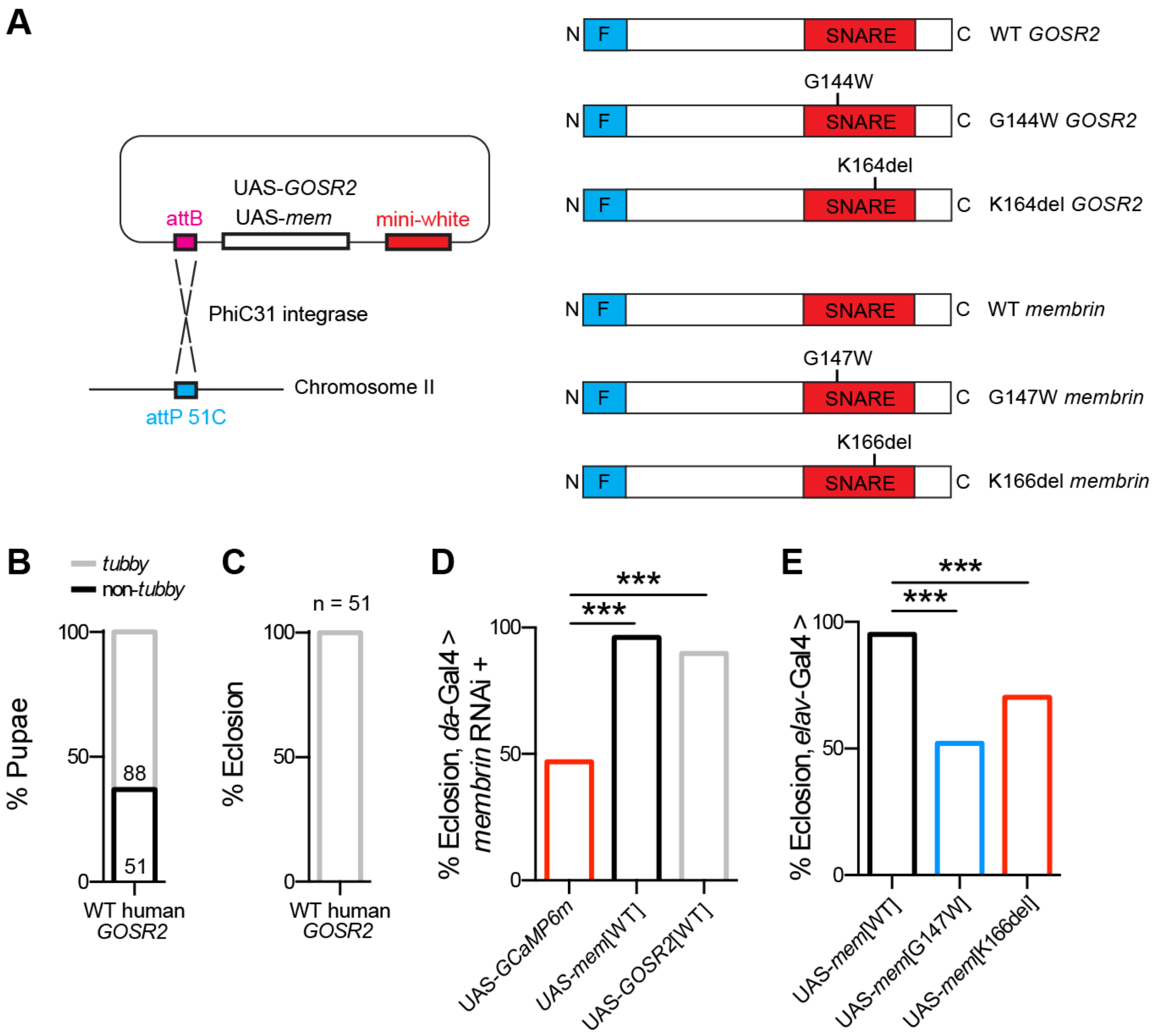

Figure S3. Human Membrin can functionally replace *Drosophila* Membrin, Related to Figure 3
(A) Schematic diagram illustrating human *GOSR2* and *Drosophila membrin* N-terminally FLAG (F) tagged transgenes and their site-specific genomic integration.
(B) FLAG-tagged human Membrin was globally expressed with the *daughterless*-Gal4 driver in a *membrin* null background according to the same strategy as in Figure 3. Rescue to the pupal stage is shown. Non-*tubby* pupae indicate the desired genotypes resulting from the genetic cross described in the Methods section.
(C) All scored non-*tubby* animals managed to eclose from their pupal cases.
(D) Membrin RNAi induced eclosion deficits can be rescued by co-overexpression of WT *Drosophila* UAS-*membrin* and WT human UAS-*GOSR2*. These effects are compared to *membrin* RNAi co-overexpression with an unrelated UAS-*GCaMP6m*, which serves to control for the presence of an additional UAS-element in these experiments. n = 164, 301, 215 for *GCaMP6m*, WT *membrin* and *GOSR2* co-overexpression.
(E) Neuronal overexpression of mutant UAS-*membrin* in wild type Membrin animals with *elav*-Gal4 resulted in reduced eclosion. n = 183, 75, 94 for UAS-*membrin*[WT]/[G147W]/[K166del].
Fisher's exact test with Bonferroni correction (D, E).

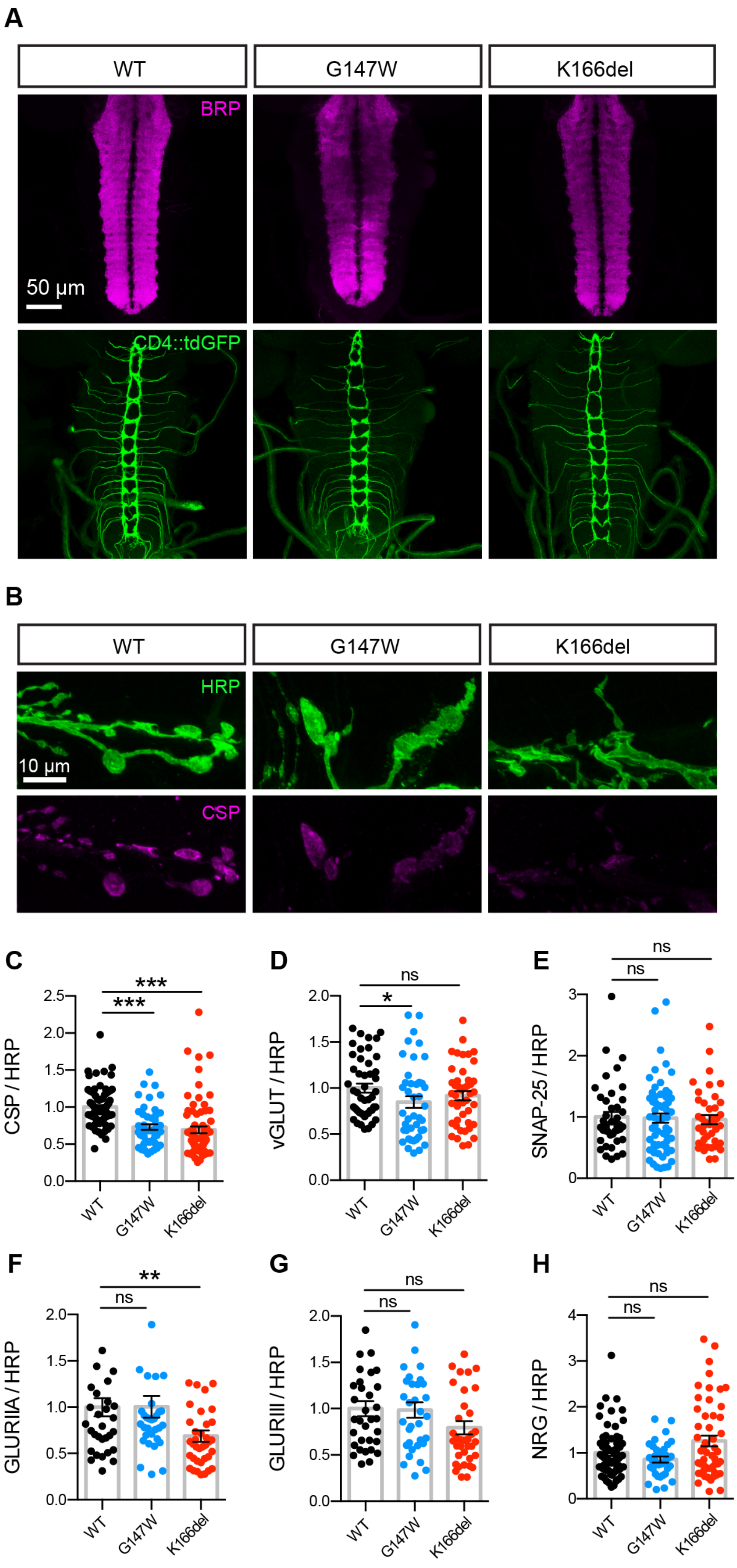
A
WT
G147W
K166del
BRP
50 µm
CD4::tdGFP
B
WT
G147W
K166del
HRP
10 µm
CSP
C
CSP / HRP
***
***
D
vGLUT / HRP
ns
*
E
SNAP-25 / HRP
ns
ns
F
GLURIIA / HRP
**
ns
G
GLURIII / HRP
ns
ns
H
NRG / HRP
ns
ns
WT
G147W
K166del

Figure S4. Sensory axonal projections and endogenous synaptic cargos, Related to Figure 4
(A) Saturated immuno-fluorescent confocal z-stacks showing projection of sensory *ppk*-neuron axons into the L3 larval ventral nerve cord (VNC). Neuropil regions in the VNC are labeled with Bruchpilot (BRP). *ppk*-neuron axons express membrane-tagged CD4::tdGFP. In Mem-WT, Mem-G147W and Mem-K166del backgrounds, no apparent defect in the growth and targeting of *ppk*-neuron axons was observed.
(B) Example confocal z-stack maximum projections of synaptic boutons immuno-labeled with antibodies against HRP and the synaptic vesicle protein Cysteine String Protein (CSP).
(C-H) Fluorescent intensity of immuno-labeled presynaptic CSP (C), vGLUT (D) and SNAP-25 (E); post-synaptic GLURIIA (F) and GLURIII (G); and trans-synaptic NRG (H) in Mem-WT, Mem-G147W and Mem-K166del synapses. Data points represent mean fluorescent intensity in a single synaptic bouton as a fraction of the corresponding HRP signal and normalized to the mean of Mem-WT. Data were acquired from ≥ 7 larvae. n = 77, 47, 72 (C); 47, 42, 45 (D); 42, 60, 41 (E); 33, 33, 37 (F); 33, 33, 37 (G); 69, 34, 52 (H) for Mem-WT/-G147W/-K166del.
Mean and SEM are shown. *, **, *** represent $p < 0.05, 0.01, 0.001$, ns = not significant ($p > 0.05$); Kruskal-Wallis test with Dunn's post-hoc test.

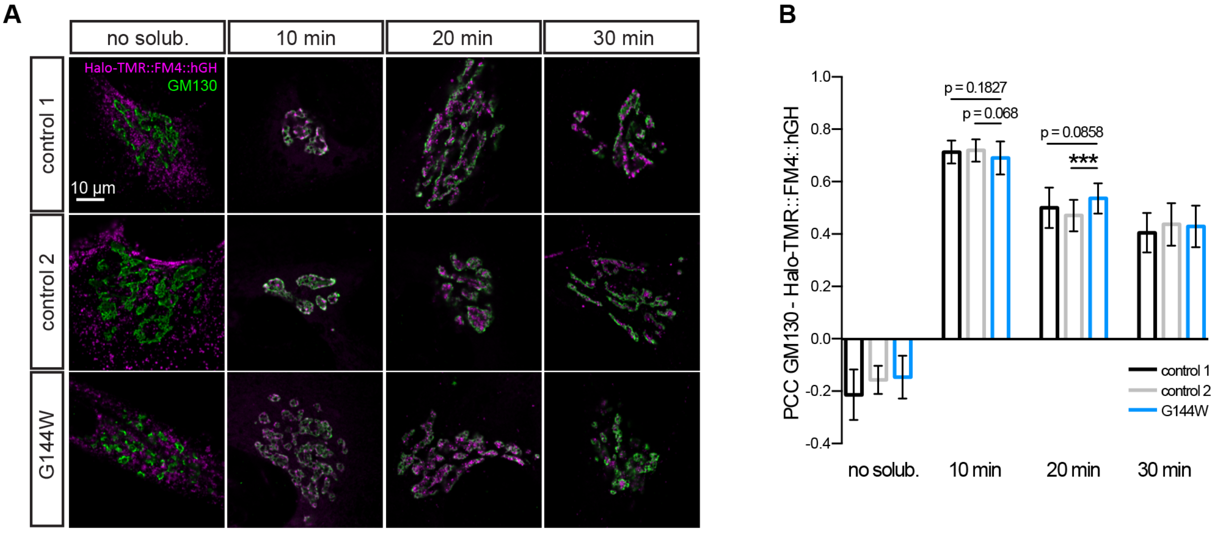

Figure S5. Golgi trafficking in patient fibroblasts, Related to Figure 5

(A) Confocal slices of control 1 & 2 and G144W mutant Membrin fibroblasts overexpressing the artificial Halo::FM4::hGH cargo loaded with TMR. When no D/D solubilizer was present the cargo remained in the ER due to aggregation of the FM4 domains. 10 min after solubilization significant colocalization with the cis-Golgi marker GM130 was apparent, which was decreased after 20 and 30 min.

(B) Quantification of experiment as described in (A). Pearson's correlation coefficients of Halo-TMR::FM4::hGH and GM130 were calculated for each time point. Number of cells quantified for control 1 & 2 and G144W are as follows: no solubilizer – 23, 21, 23; 10 min – 27, 28, 27; 20 min – 26, 29, 28; 30 min – 27, 26, 28. Mean and SD are shown.

*** represents $p < 0.001$; ns = not significant ($p > 0.05$); one-way ANOVA with Dunnett's multiple comparison test.

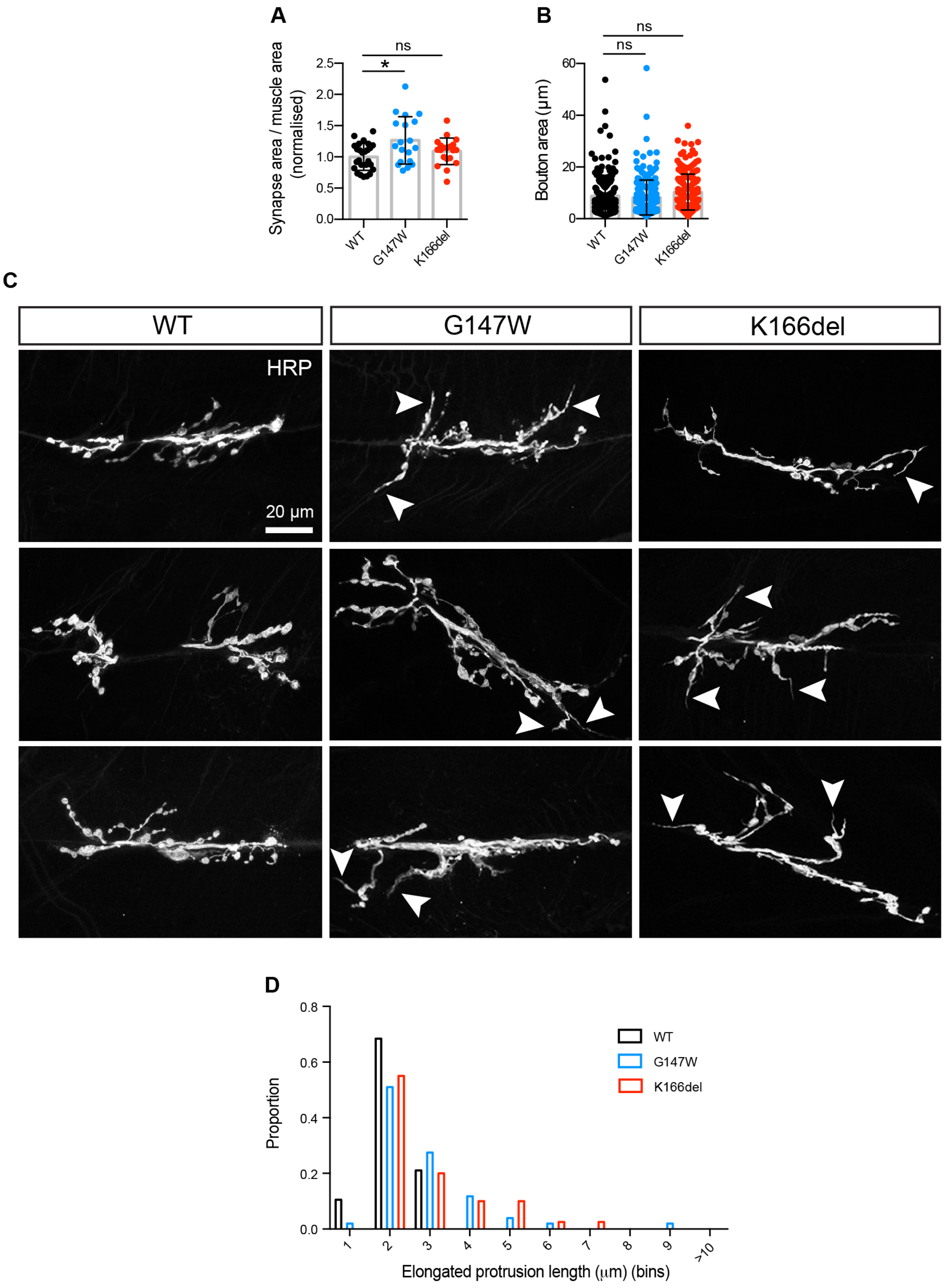

Figure S6. NMJ size and elongated protrusions, Related to Figure 5
(A) Synapse area normalized to muscle area at muscle 6/7, segment 3 of Mem-WT, Mem-G147W and Mem-K166del L3 larvae. Data are expressed relative to Mem-WT. n = 26, 19, 20 for Mem-WT/G147W/K166del.
(B) Areas of synaptic boutons of motor neurons innervating muscle 6/7, segment 3. n = 247, 237, 182 for Mem-WT/G147W/K166del.

(C) Further example confocal z-stacks of HRP-labeled motor neurons innervating muscle 6/7, segment 3 of L3 larvae, as shown in Figure 5D. Arrowheads indicate elongated protrusions frequently seen in Mem-G147W and Mem-K166del.

(D) Frequency distribution of elongated protrusion lengths at terminal boutons. Elongated protrusions in Mem-G147W and Mem-K166del exhibit a shift towards longer lengths relative to Mem-WT. n = 19, 51, 40 for Mem-WT/G147W/K166del.

Replicate values, mean and SD are shown unless otherwise stated. * represents $p < 0.05$, ns = not significant ($p > 0.05$); Kruskal-Wallis test with Dunn's post-hoc test.

Figure S7

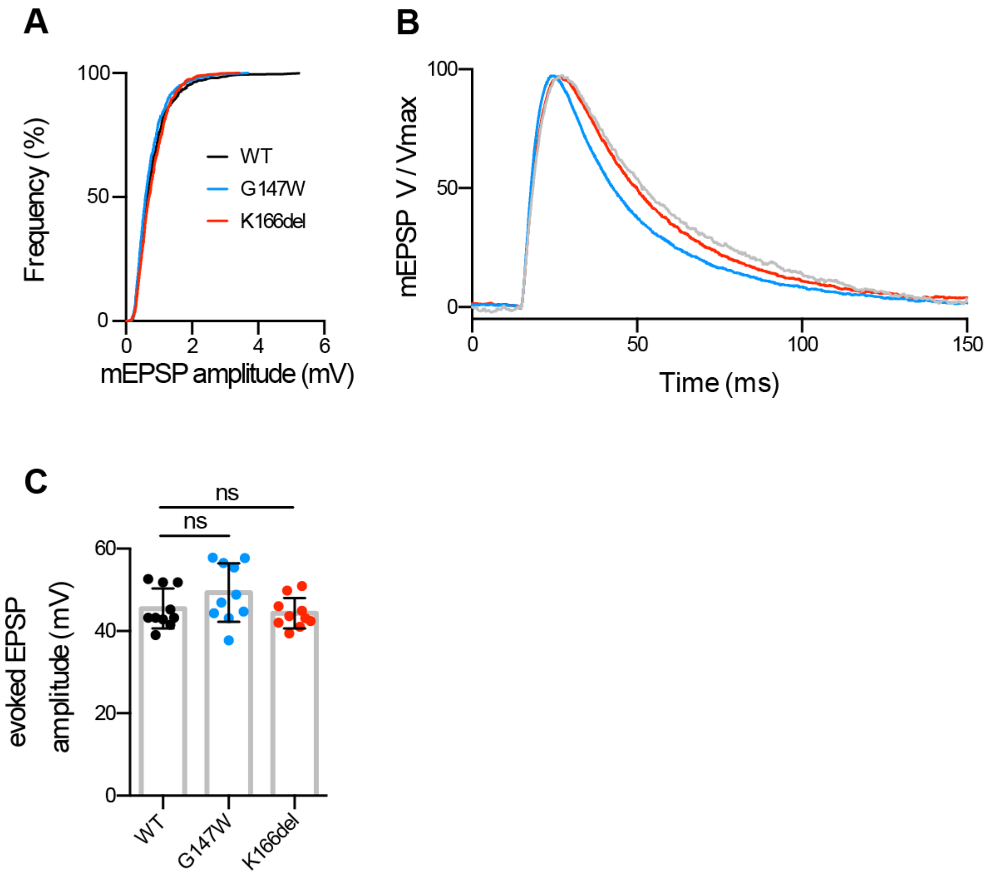

Figure S7. mEPSP amplitude, time course and evoked EPSP amplitude, Related to Figure 7
(A) Cumulative frequency plot of mEPSP amplitudes of recordings as described in Figure 7. 800 events per genotype, from 8 animals each, are shown.
(B) Average mEPSP time courses normalized to the peak are shown. There was no significant difference in the τ of a single exponential fit to the decay between Mem-G147W/Mem-K166del and Mem-WT. Each average is derived from the mean mEPSP calculated from recordings in 8 animals.
(C) Evoked EPSP amplitudes are not significantly different between Mem-WT, Mem-G147W and Mem-K166del. n = 10.
Replicate values, mean and SD are shown. ns = not significant ($p > 0.05$); one-way ANOVA with Dunnett's multiple comparison test (B, C).

SUPPLEMENTAL EXPERIMENTAL PROCEDURES

Molecular biology and Bioinformatics

GOSR2 constructs were also cloned into pcDNA3.1(-) by replacing the CDS of pcDNA3.1(-) mouse C/EBP beta (LAP) (Addgene plasmid #12557) for these inserts via NotI and KpnI (NEB), giving rise to pcDNA3.1(-)_*GOSR2*[WT], pcDNA3.1(-)_*GOSR2*[G144W] and pcDNA3.1(-)_*GOSR2*[K164del]. pGEX-2T-GST::*Sed5*, pET28a-His::*Sec22*, pET28a-His::*Bet1* and pET28a-His::*Bos1*[WT] were previously described (Parlati et al., 2000; 2002). To generate pET28a-His::*Bos1*[G176W] and pET28a-His::*Bos1*[D196del], the respective mutations were introduced with the QuickChange Site-Directed Mutagenesis Kit (Agilent Technologies). The Halo::FM4::hGH containing plasmid was previously cloned in the Rothman lab (Lavieu et al., 2013). SNARE motifs in human and *Drosophila* Membrin, as well as yeast Bos1, were identified as described previously (Kloepper et al., 2007) and aligned with Clustal Omega (McWilliam et al., 2013). Conserved residues were highlighted using BoxShade.

Liposome fusion assays

In this assay, v-SNARE-containing liposomes are loaded with the fluorophores Nitro-2-1,3 benzoxadiazol-4yl-phosphatidylethanolamine (NBD-PE) and rhodamine-PE. Both fluorophores are lipid-bound and exhibit robust Förster resonance energy transfer. Thus, NBD fluorescence is quenched in the presence of rhodamine. When v-SNARE liposomes fuse with unlabeled t-SNARE-containing liposomes, NBD and rhodamine molecules become spatially separated, NBD is de-quenched and NBD fluorescence increases, providing a measure of liposome fusion (Struck et al., 1981). Wild-type (WT) and mutant Bos1 along with the SNARE partners Bet1, Sec22 and Sed5 (orthologous to mammalian Syntaxin-5) were purified in *Escherichia coli*. GST-Sed5 was purified using glutathione agarose affinity beads (Thermo Fisher Scientific) and the GST-tag was removed using 100U of human thrombin (Sigma) with overnight incubation at 4°C in Buffer A (25 mM HEPES pH 7.4, 400 mM KCl, 10% glycerol, 1 mM DTT, 1% n-Octyl-β-D-glucopyranoside) containing 2 mM $CaCl_2$. The other proteins – Sec22, Bet1, WT and G176W/D196del mutant Bos1 – were purified with a His^6-tag using HisPur Ni-NTA affinity beads (Thermo Fisher Scientific) in Buffer A containing 300 mM imidazole, pH 7.5. For the t-SNARE acceptor liposomes, 27 µM of Sed5, Sec22 and WT or G176W/D196del mutant Bos1 in 500 µl were incubated overnight at 4°C and then incorporated into palmitoyl-2-oleoyl phosphatidylcholine (POPC): 1,2 dioleoyl phosphatidylserine (DOPS) at 85:15 mol% liposomes. The donor liposomes containing Bet1 (1:100 protein:lipid ratio) were prepared similarly with the lipid mix of POPC, DOPS and the fluorescent probes NBD-PE and rhodamine-PE (1.5 mol% each). All lipids were purchased from Avanti Polar lipids (Alabaster, AL, USA). Liposome fusion assay was performed by mixing 5 µl of the donor liposome (Bet1) with 45 µl of the acceptor liposome (t-SNAREs). Fusion of liposomes was monitored by the change in NBD fluorescence at 538 nm using a Flexstation 3 microplate reader (Molecular Devices). After 120 min, 10 µl of 5% w/v *n*-dodecyl-β-maltoside (Thermo Fisher Scientific) was added to lyse all vesicles to estimate the maximum NBD fluorescence (Weber et al., 1998). For experiments with the Bet1 peptide, the t-SNARE liposomes were pre-incubated at 37°C for 45 min with 50 µM peptide corresponding to the C-terminal half of Bet1 (RGSNQTIDQLGDTFHNTSVKLKRTFGNMMEMA, Vc peptide) prior to addition of Bet1 liposomes to initiate fusion.

Cell culture and transfections

Fibroblast and HEK293T cells were grown in DMEM + 10% FBS at 37°C and 5% CO_2. Fibroblast transfections were carried out with lipofectamine 2000 (Thermo Fisher Scientific), HEK293T transfections with Effectene (Qiagen).

Fibroblast imaging

The following antibodies were used for confocal imaging of fibroblasts: mouse anti-Membrin (clone 25, BD Biosciences; This antibody was raised against Membrin residues 5-124 and therefore should not be affected by the G144W mutation.), mouse anti-FLAG (M2 clone, Sigma), rat anti-FLAG (Agilent Technologies), rabbit anti-GPP130 (Cambridge Bioscience), mouse anti-GM130 (clone 35, BD Biosciences) rabbit anti-PDI (Sigma), and goat anti-mouse/rabbit/rat Alexa Fluor 488/555/647 conjugated secondaries (Thermo Fisher Scientific). For Golgi trafficking studies cells were loaded with HaloTag TMR Ligand (Promega) 24 h post transfection with Halo::FM4::hGH. Subsequently ER retained cargo was released by addition of D/D solubilizer 1.5 µM (Clontech). Fixed samples were imaged with a Plan-Apochromat 63x 1.4 NA oil immersion objective on Zeiss confocal LSM710 or LSM880 microscopes.

Western blot

The following antibodies were used for western blotting: mouse anti-Membrin (clone 25, BD Biosciences), mouse anti-β-actin (clone AC-74, Sigma) and HRP-conjugated anti-mouse (Jackson Immuno). For semi-

quantitative western blots, detection was carried out with SuperSignal West Pico Chemiluminescent Substrate (Thermo Fisher Scientific) and a ChemiDocTM Imaging system (Bio-Rad). Band intensities were extracted with Image Studio Lite (Li-cor).

Drosophila genetics and phenotyping

To evaluate the lethal phase of *membrin*^{1524} at the L1 and L2 larval stages, we placed this allele over the fluorescent TM3 *Kr* > GFP balancer chromosome (Casso et al., 2000), which was also backcrossed to iso31 for five generations. *GOSR2* and *membrin* transgenic flies were generated by microinjection of pUASTattB_FLAG::*GOSR2*[WT/G144W/K164del]/pUASTattB_FLAG::*membrin*[WT/G147W/K166del] into *y*[1] M{vas-int.Dm}ZH-2A w*; M{3xP3-RFP.attP'}ZH-51C embryos (Cambridge fly facility). This approach enabled us to retrieve WT and mutant UAS-*GOSR2*/UAS-*membrin* transgenic fly lines with insertions into precisely the same genomic locus (ZH-51C), which is important to provide comparable transgene expression levels (Bischof et al., 2007). In order to express WT or mutant *GOSR2* or *membrin* in a *membrin* null background the following stocks were created and crossed to each other: I. *w*[1118]; UAS-FLAG::*GOSR2*[WT/G144W/K164del]; *membrin*^{1524}/TM6B, *tb* or *w*[1118]; UAS-FLAG::*membrin*[WT/G147W/K166del]; *membrin*^{1524}/TM6B, *tb* and II. *w*[1118]; +; *membrin*^{1524}, *daughterless*-GAL4/TM6B, *tb*. Each component of these flies was outcrossed for five generations into iso31 prior to assembly by standard mating schemes. For dendritic analysis, *ppk*-CD4::tdGFP (Han et al., 2011) was incorporated into stock II. (*w*[1118]; *ppk*-CD4::tdGFP; *membrin*^{1524}, *daughterless*-Gal4/TM6B, *tb*) and crossed to stock I. To assess viability of *membrin* mutant animals, the above I x II crosses were allowed to egg-lay onto apple-juice agar plates overnight. Subsequently, eggs/embryos were counted and transferred to standard food tubes. After completion of pupation, non-*tubby* pupae were counted. Given that theoretically only one quarter of the collected egg/embryos are of the correct genotype, we used the following formula to calculate egg/embryo to pupa viability: non-*tubby* pupae/(total eggs/4). The resulting fraction was normalized to WT because we found a considerable reduction with consecutive egg-lays, presumably reflecting decreasing fertilization. Eclosion rates were determined 11 and 12 days after onset of egg laying, a time point where under our conditions non-eclosion was equal to death or imminent death in the pupal case. To assess locomotion, L3 larvae were placed in the center of a 90 mm sucrose-agar plate positioned on top of a 4 mm grid, allowed to settle for 30 s, and filmed for the next 60 s. Grid-breaks in this time period were then assessed offline.

Dendritic analysis

To extract total dendrite length and to serve as a template for the ImageJ Sholl Analysis plugin, dendrites were semi-manually traced with the ImageJ NeuronJ plugin (Ferreira et al., 2014; Meijering et al., 2004). Terminal branches were manually counted on dendrite tracings with the ImageJ multi-point tool. For FRAP experiments, L3 larvae were fillet-prepped in HL3 saline without Ca^{2+} (70 mM NaCl, 5 mM KCl, 20 mM $MgCl_2$, 10 mM $NaHCO_3$, 5 mM trehalose, 115 mM sucrose, 5 mM HEPES, pH 7.2). Fillets were transferred to a #1.5 glass bottom dished and submerged in fresh HL3 saline with a custom-made platinum wire anchor before being imaged on an inverted Zeiss confocal LSM510 with a Plan-Apochromat 20x 0.8 NA objective. Bleaching of a 50 μm^2 area encompassing major primary dendrites directly adjacent to the soma was carried out by scanning for 200 iterations with 100% 488 nm transmission. Mean fluorescence intensity of a small dendritic region contained in a 5 µm diameter circle 25 µm from the bleach border adjacent to the soma served as a read-out. This is the most distant dendritic region from either bleach margin and thus the contribution to fluorescence recovery from lateral diffusion of dendrite surface localizing CD4::tdGFP is minimized. Bleach depth in this region was consistently greater than 87%.

Immuno-histochemistry of larval neuromuscular junctions and brains

Late-stage L3 larvae were dissected in low Ca^{2+} (0.2 mM) HL3 (see above). Dissected NMJ preparations were fixed in either 4% PFA or Bouin's solution for 10-20 min at room temperature. Larval brains were dissected and immunostained as described previously (Wu and Luo, 2006). The following antibodies were used: mouse anti-BRP (clone nc82, DSHB), anti-CSP (clone 6D6, DSHB), anti-GLURIIA (clone 8B4D2, DSHB), and anti-Futsch (clone 22C10, DSHB); rabbit anti-ANK2-XL (kind gift from Herman Aberle), rabbit anti-SNAP-25 (kind gift from David Deitcher), rabbit anti-GLURIII and rabbit anti-vGLUT (kind gifts from Aaron DiAntonio), mouse anti-GFP (clone 3E6, Thermo Fisher Scientific), goat anti-HRP Alexa Fluor 488 conjugated (Jackson ImmunoResearch) and secondaries as above.

For quantification of synaptic development, all experiments were performed and analysed blind to experimental genotype. Boutons were identified using anti-HRP and anti-CSP (Zinsmaier et al., 1990). Both type 1b and type 1s boutons were included in the bouton count. The number of BRP puncta and CSP-positive boutons was quantified using the ImageJ 3D Object Counter Plugin (Bolte and Cordelières, 2006). Since CSP levels were reduced in *GOSR2*-PME model backgrounds, CSP fluorescent signals were enhanced to saturating levels in all genotypes prior to bouton counting.

NMJ electrophysiology and larval seizure assay

The membrane potential was set to -70 mV with current injection at the start of each recording. Voltage signals were low-pass filtered at 1.67 kHz (10 kHz 4 pole Bessel on Geneclamp 500, 1.7 kHz 8-pole Bessel on LHBF-48x) and digitised at 25 kHz by a CED-1401 plus A/D interface (Cambridge Electronic Design, UK) using Spike2 software (v. 5.13) (CED, Cambridge, UK). Synaptic potentials were analysed offline using Strathclyde Electrophysiology Software WinEDR (v3.5.2) and GraphPad Prism (v.6). All synaptic events were verified manually. Recordings were discarded if the initial resting membrane potential was more positive than -60 mV or varied by more than 10% throughout the recording. mEPSPs were recorded for a minimum of 5 min. Single EPSPs were evoked 10 times at a standard frequency of 0.033 Hz or trains of 5 EPSPs at 10 Hz were evoked 3 times at 0.033 Hz. Intervals and amplitudes of mEPSPs were compared by creating a cumulative distribution for each genotype of 800 measurements across 8 animals, with each animal contributing 100 values. To analyse the mEPSP waveform, a mean mEPSP was constructed for each recording from events that showed only a single clear peak and a smooth decay so as to prevent distortion of the waveform by closely-occurring mEPSPs. To induce larval seizures, a 30 V DC pulse was applied towards the larval CNS for 3 s by using a Grass S88 stimulator (Grass instruments, RI, USA). This induced seizures in wild-type and mutant animals and the time until normal crawling behavior was resumed (recovery time) served as a read-out for seizure severity.

SUPPLEMENTAL REFERENCES

Bolte, S., and Cordelières, F.P. (2006). A guided tour into subcellular colocalization analysis in light microscopy. J Microsc *224*, 213–232.

Casso, D., Ramírez-Weber, F., and Kornberg, T.B. (2000). GFP-tagged balancer chromosomes for *Drosophila melanogaster*. Mech Dev. 91, 451–454.

Ferreira T, Blackman A, Oyrer J, Jayabal A, Chung A, Watt A, Sjöström J, and van Meyel D. (2014), Neuronal morphometry directly from bitmap images. Nat Methods *11*, 982–984.

McWilliam, H., Li, W., Uludag, M., Squizzato, S., Park, Y.M., Buso, N., Cowley, A.P., and Lopez, R. (2013). Analysis Tool Web Services from the EMBL-EBI. Nucleic Acids Res. *41*, W597–W600.

Meijering, E., Jacob, M., Sarria, J.-C.F., Steiner, P., Hirling, H., and Unser, M. (2004). Design and validation of a tool for neurite tracing and analysis in fluorescence microscopy images. Cytom Part A *58*, 167–176.

Struck, D.K., Hoekstra, D., and Pagano, R.E. (1981). Use of resonance energy transfer to monitor membrane fusion. Biochemistry *20*, 4093–4099.

Wu, J.S., and Luo, L. (2006). A protocol for dissecting *Drosophila melanogaster* brains for live imaging or immunostaining. Nat Protoc *1*, 2110–2115.

Zinsmaier, K.E., Hofbauer, A., Heimbeck, G., Pflugfelder, G.O., Buchner, S., and Buchner, E. (1990). A cysteine-string protein is expressed in retina and brain of *Drosophila*. J Neurogenet *7*, 15–29.
