# Supplementary material for: Mutations in Membrin/GOSR2 Reveal Stringent Secretory Pathway Demands of Dendritic Growth and Synaptic Integrity
Source: Cell Rep. 2017 Oct 3;21(1):97–109. doi: 10.1016/j.celrep.2017.09.004 (PMC5640804; doi:10.1016/j.celrep.2017.09.004)
Supplement: Document S2. Article plus Supplemental Information [file mmc2.pdf]

Cell Reports

Article

Mutations in Membrin/*GOSR2* Reveal Stringent Secretory Pathway Demands of Dendritic Growth and Synaptic Integrity

Graphical Abstract

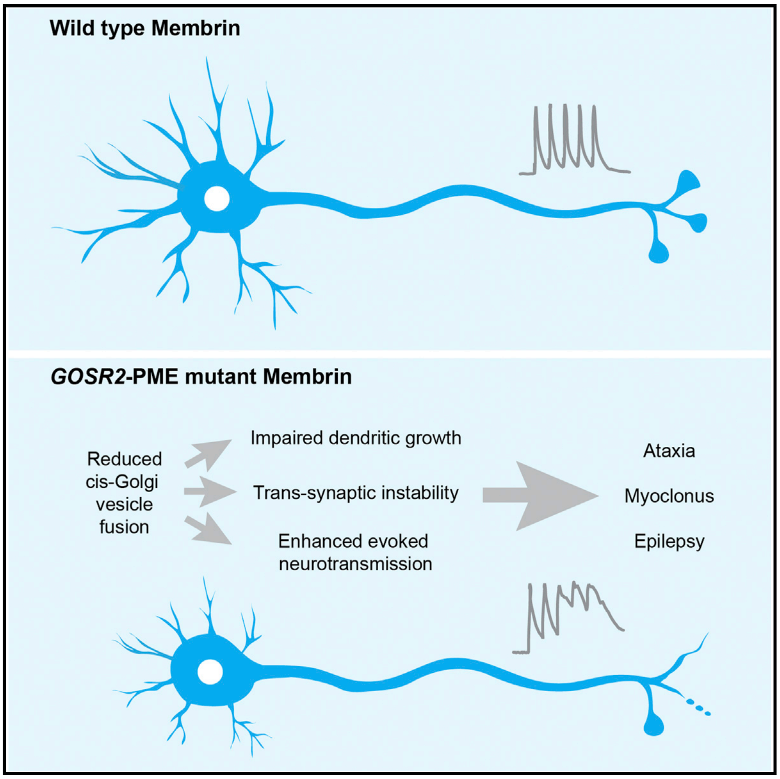

Highlights

- Epilepsy/ataxia-linked mutations in Membrin confer partial Golgi SNARE defects
- Partial reductions in membrane trafficking strongly impair dendritic growth
- Synaptic morphology and function tightly depend upon efficient secretory trafficking

Authors

Roman Praschberger, Simon A. Lowe, Nancy T. Malintan, ..., James J.L. Hodge, James E. Rothman, James E.C. Jepson

Correspondence

j.jepson@ucl.ac.uk

In Brief

In this study, Praschberger et al. utilize in vitro assays, patient-derived cells, and *Drosophila* models to unravel how mutations in the essential Golgi SNARE protein Membrin cause progressive myoclonus epilepsy and to demonstrate a selective vulnerability of developing neurons to partial impairment of ER-to-Golgi trafficking.

Praschberger et al., 2017, Cell Reports *21*, 97–109
October 3, 2017 © 2017 The Authors.
https://doi.org/10.1016/j.celrep.2017.09.004

OPEN ACCESS
CellPress

Mutations in Membrin/*GOSR2* Reveal Stringent Secretory Pathway Demands of Dendritic Growth and Synaptic Integrity

Roman Praschberger,[1] Simon A. Lowe,[2,6] Nancy T. Malintan,[1,6] Carlo N.G. Giachello,[3] Nian Patel,[1] Henry Houlden,[4] Dimitri M. Kullmann,[1] Richard A. Baines,[3] Maria M. Usowicz,[2] Shyam S. Krishnakumar,[1,5] James J.L. Hodge,[2] James E. Rothman,[1,5] and James E.C. Jepson[1,7,*]

[1]Department of Clinical and Experimental Epilepsy, UCL Institute of Neurology, London, UK
[2]School of Physiology, Pharmacology, and Neuroscience, University of Bristol, Bristol, UK
[3]Faculty of Biology, Medicine, and Health, Division of Neuroscience & Experimental Psychology, Manchester Academic Health Science Centre, University of Manchester, Manchester, UK
[4]Department of Molecular Neuroscience, UCL Institute of Neurology, London, UK
[5]Department of Cell Biology, Yale School of Medicine, New Haven, CT, USA
[6]These authors contributed equally
[7]Lead Contact
*Correspondence: j.jepson@ucl.ac.uk
https://doi.org/10.1016/j.celrep.2017.09.004

SUMMARY

Mutations in the Golgi SNARE (SNAP [soluble NSF attachment protein] receptor) protein Membrin (encoded by the *GOSR2* gene) cause progressive myoclonus epilepsy (PME). Membrin is a ubiquitous and essential protein mediating ER-to-Golgi membrane fusion. Thus, it is unclear how mutations in Membrin result in a disorder restricted to the nervous system. Here, we use a multi-layered strategy to elucidate the consequences of Membrin mutations from protein to neuron. We show that the pathogenic mutations cause partial reductions in SNARE-mediated membrane fusion. Importantly, these alterations were sufficient to profoundly impair dendritic growth in *Drosophila* models of *GOSR2*-PME. Furthermore, we show that Membrin mutations cause fragmentation of the presynaptic cytoskeleton coupled with transsynaptic instability and hyperactive neurotransmission. Our study highlights how dendritic growth is vulnerable even to subtle secretory pathway deficits, uncovers a role for Membrin in synaptic function, and provides a comprehensive explanatory basis for genotype-phenotype relationships in *GOSR2*-PME.

INTRODUCTION

Secreted, membrane, endosomal, and lysosomal proteins are deposited into the endoplasmic reticulum (ER) after ribosomal synthesis. Subsequently, these proteins exit the ER, transition through the Golgi apparatus, and reach their ultimate target sites via the trans-Golgi network, a biosynthetic route termed the secretory pathway (Palade, 1975). The transport of proteins along this path is facilitated by membrane-enclosed vesicles, and their fusion with the *cis*-Golgi is mediated by the target (t-) SNARE (SNAP [soluble NSF attachment protein] receptor) proteins Membrin (also known as GS27; encoded by the *GOSR2* gene), Sec22b, and Syntaxin-5, in concert with the vesicle (v-) SNARE Bet1 (Parlati et al., 2000; Xu et al., 2000). Similar to other intracellular fusion steps, these proteins are necessary for fusion of opposing lipid bilayers through the formation of a quaternary SNARE complex (Hay et al., 1997; Lowe et al., 1997; Parlati et al., 2000; Volchuk et al., 2004). Critical to this process is the N- to C-terminal zippering along 15 mostly hydrophobic "layer" amino acids (−7 to +8) within the SNARE domain of each protein (Gao et al., 2012; Sutton et al., 1998).

Homozygous missense (G144W: layer −3) or compound heterozygous missense and deletion mutations (G144W and K164del: between layer +2 and +3) in the Membrin SNARE motif have recently been shown to cause the severe neurological syndrome progressive myoclonus epilepsy (PME) (Corbett et al., 2011; Praschberger et al., 2015). Patients with this form of PME, termed *GOSR2*-PME, typically present with ataxia at ~3 years of age, followed by cortical myoclonus and generalized tonic-clonic seizures. Despite rapid disease progression and frequent premature death, cognitive function usually remains remarkably preserved. Correspondingly, marked neurodegeneration as an underlying primary cause has not been reported (Boissé Lomax et al., 2013; Corbett et al., 2011; Praschberger et al., 2015; van Egmond et al., 2014, 2015). Given the critical role of Membrin in ER-to-Golgi trafficking and its fundamental importance in every cell of the human body, it is unclear why Membrin mutations specifically result in nervous system dysfunction and do not cause symptoms in other organs. No paralog is present in the human genome that could functionally replace Membrin in non-neuronal cells and therefore explain the primarily neuronal phenotype.

In the present study, we set out to unravel the neuronal bottleneck of *GOSR2*-PME. To do so, we investigated the disease mechanism of *GOSR2*-PME from molecule to neuron utilizing reconstituted liposome fusion assays, patient-derived fibroblasts, and *Drosophila* models. We found that the pathogenic Membrin SNARE motif mutations result in a partial loss of function that is nonetheless sufficient to robustly reduce dendritic growth in vivo.

This is an open access article under the CC BY license (http://creativecommons.org/licenses/by/4.0/).

OPEN
ACCESS
CellPress

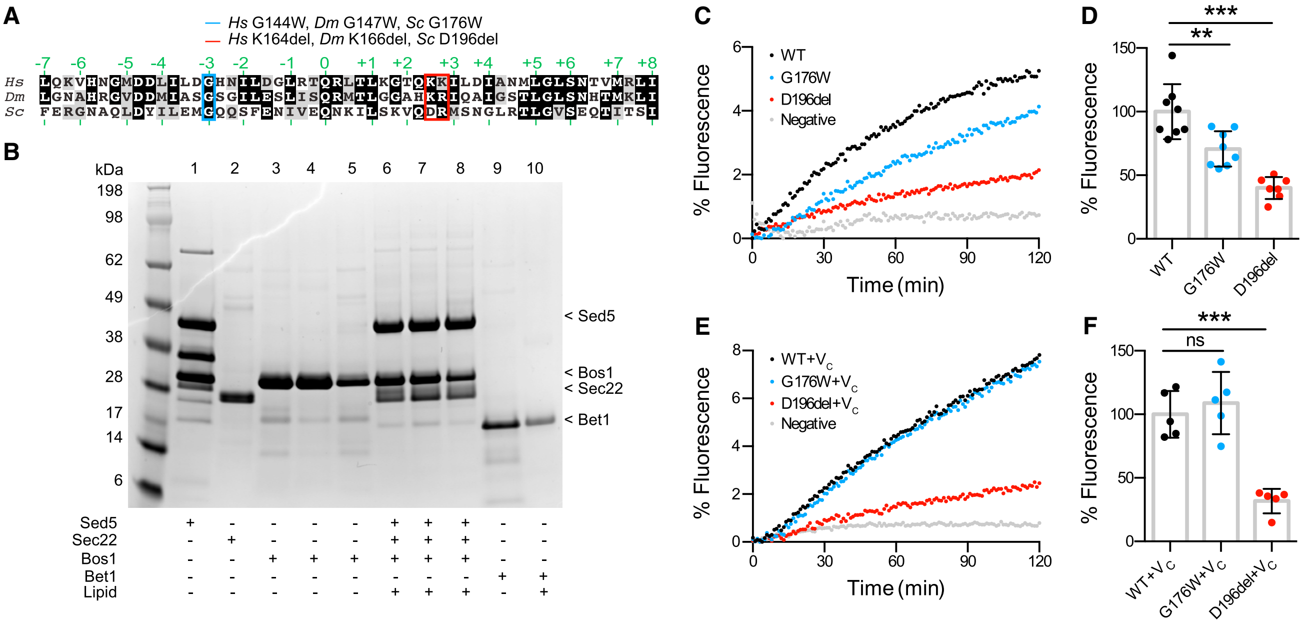

Figure 1. Reduced Liposome Fusion Rates due to Orthologous *GOSR2*-PME Mutations

(A) SNARE domain alignment of *Homo sapiens* (*Hs*), *Drosophila melanogaster* (*Dm*), and *Saccharomyces cerevisiae* (*Sc*) Membrin (UniProt: O14653-1), Membrin (UniProt: Q9VRL2), and Bos1 (UniProt: P25385), respectively. Layer amino acids critical for forming the tetrameric *cis*-Golgi SNARE complex are indicated in green. The disease-causing G144W and K164del (one of two consecutive lysines is deleted) and the *Drosophila* and yeast orthologous residues are highlighted in blue and red.

(B) Yeast Golgi SNARE proteins Sed5 (lane 1), Sec22 (lane 2), WT Bos1 (lane 3) and G176W/D196del Bos1 mutants (lane 4/5) were purified and reconstituted into acceptor liposomes as t-SNARE complexes comprised of Sed5/Sec22/Bos1 (lanes 6/7/8, respectively). Overall stoichiometry of Sed5/Sec22/Bos1 was ~1.0×/1.0×/1.2× (Figure S1). Yeast Golgi SNARE protein Bet1 was purified (lane 9) and reconstituted into donor liposome (lane 10) containing 7-nitro-2-1,3-benzoxadiazol-4-yl (NBD)-phosphoethanolamine (PE) and rhodamine-PE fluorescent lipids.

(C) Example traces showing increase in NBD fluorescence due to fusion between WT or G176W/D196del Bos1-containing t-SNARE complex acceptor liposomes and Bet1 donor liposomes. Data are expressed as a fraction of maximal NBD fluorescence after addition of detergent.

(D) Endpoint (120 min) quantification of experiment as described in (C), normalized to WT. n = 8, 8, 7 for WT, G176W and D196del.

(E) Example traces of experiment as in (C) with the modification that 50 μM of a peptide comprising the C-terminal half of the Bet1 SNARE domain (V_C) was added.

(F) Endpoint (120 min) quantification of experiment as described in (E), normalized to WT (n = 5).

Replicate values, mean, and SD are shown. **$p < 0.01$; ***$p < 0.001$; ns, not significant ($p > 0.05$); one-way ANOVA with Dunnett's multiple comparison test.

Membrin mutations also resulted in presynaptic retraction and physiological abnormalities at motor synapses. Together, our results suggest a mechanistic basis for the multifaceted neurological features of *GOSR2*-PME patients, highlight tight trafficking demands of growing dendrites, and illustrate a close-knit dependence of synaptic integrity and neurotransmitter release on cargo trafficking through the Golgi apparatus.

RESULTS

GOSR2-PME Mutations Result in Partial SNARE Dysfunction

The locations of the PME-causing G144W and K164del mutations in the Membrin SNARE domain suggest defective assembly of the quaternary *cis*-Golgi SNARE complex and thus reduced fusion of vesicular cargo carriers with this compartment. Given the technical difficulties associated with producing mammalian Golgi SNAREs, and since mammalian and yeast Golgi SNAREs are functionally conserved (McNew et al., 1997; Fischer von Mollard and Stevens, 1998; Varlamov et al., 2004), we tested for SNARE defects using a well-established yeast SNARE protein liposome fusion assay (McNew et al., 2000; Parlati et al., 2000) (see Supplemental Experimental Procedures for details). We introduced the corresponding PME-linked G176W/D196del mutations into the Membrin yeast ortholog Bos1 (Figure 1A). Purified t-SNAREs containing Bos1, Sec22, and Sed5 (orthologous to mammalian Syntaxin-5) were subsequently preassembled and reconstituted into acceptor liposomes, while the v-SNARE Bet1 was incorporated into the fluorescent donor liposomes (Figures 1B and S1A).

Both PME mutations resulted in a reduced rate and extent of fusion compared to wild-type (WT), but fusion rates were significantly higher relative to a negative control where Bet1 was omitted (Figures 1C and 1D). The relative magnitude of the effects of the D196del and G176W mutations (~60% and 30% reductions in fusion, respectively) is consistent with their positions within the SNARE motif. The D196 deletion likely results in misalignment of the subsequent hydrophobic layers in the C-terminal half of the SNARE domain, a region that provides the critical force to drive membrane fusion (Gao et al., 2012). In contrast, the more subtle effect of the G176W mutation is consistent with an alteration in the N-terminal SNARE region

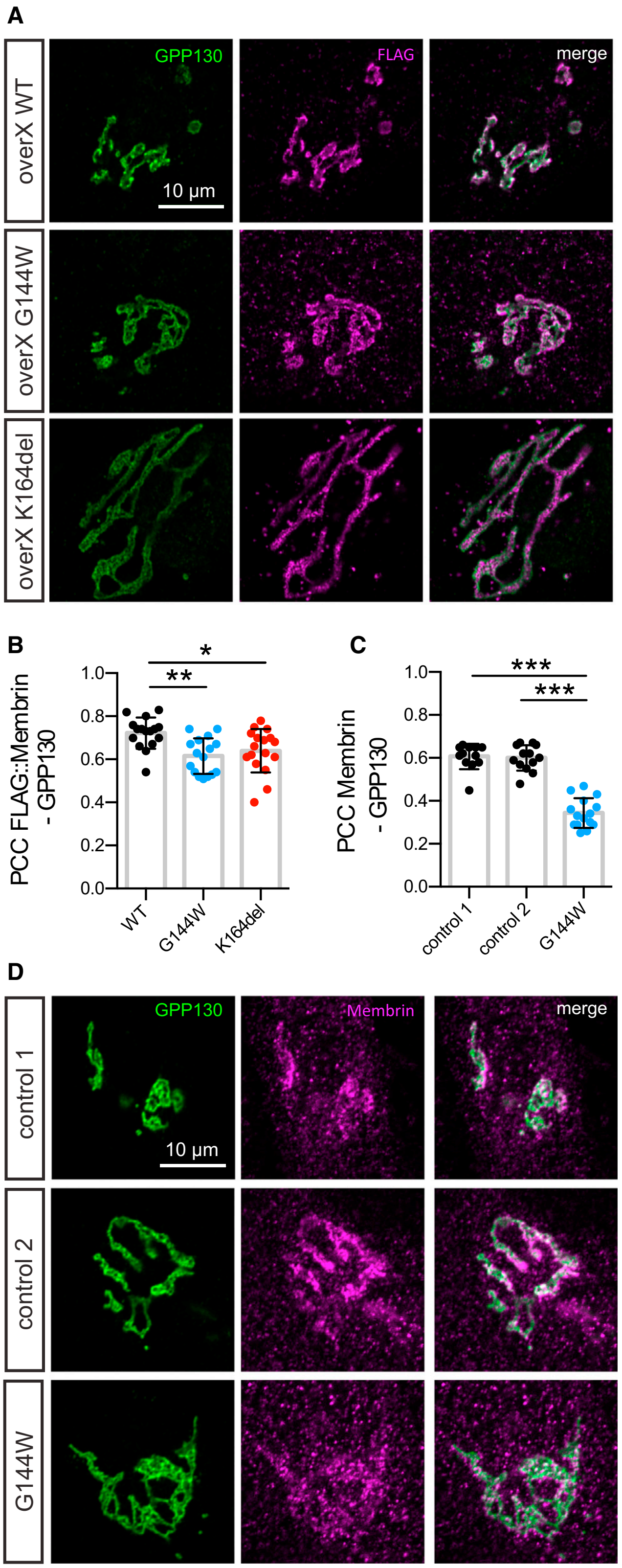

Figure 2. Mutant Membrin Retains the Capability to Localize to the *cis*-Golgi

(A) FLAG-tagged WT and G144W/K164del mutant Membrin were overexpressed in control fibroblasts and co-stained for the FLAG tag and the *cis*-Golgi resident protein GPP130. Example confocal slices are shown for each overexpressed construct.

(B) Pearson's correlation coefficients between FLAG and GP130 signals of the experiment described in (A) are shown. n = 16, 16, and 17 for WT, G144W, and K164del.

(C) Pearson's correlation coefficients between endogenous Membrin and GPP130 signals of the experiment described in (D) are shown. n = 12, 13, and 15 for control 1, control 2, and G144W.

(D) Example confocal slices of control and Membrin G144W mutant fibroblasts co-stained for endogenous Membrin and GPP130.

Replicate values, mean and SD are shown. $^{*}p < 0.05$; $^{**}p < 0.01$; $^{***}p < 0.001$; one-way ANOVA with Dunnett's multiple comparison test.

that mediates the initial engagement of SNARE domains bridging two opposing lipid bilayers. Indeed, in accordance with a selective N-terminal assembly defect, the effect of G176W but not D196del was rescued by addition of a peptide comprising of the C-terminal half of the Bet1 SNARE domain, which acts to pre-structure the N terminus (Figures 1E and 1F) (Melia et al., 2002). Increasing the pool of preassembled trans-SNARE complexes by overnight pre-incubation at 4°C also restored the fusion capacity of G176W-Bos1-containing, but not D196del-Bos1-containing, liposomes (Figures S1B and S1C). Taken together, these results suggest that the orthologous G144W and K164del mutations in Membrin partially impair distinct steps of the *cis*-Golgi SNARE complex formation, which is necessary for fusion of vesicular cargo carriers with the *cis*-Golgi (Hay et al., 1997, 1998).

Mutant Membrin Retains the Capability to Localize to the *cis*-Golgi

Only Membrin localized to the *cis*-Golgi will be capable of facilitating deposition of ER-derived cargo. Thus, we assessed the subcellular localization of overexpressed WT and G144W/K164del mutant FLAG::Membrin in primary skin fibroblasts from a healthy human control. Similar to WT, both mutants exited the ER and co-localized with the *cis*-Golgi matrix protein GPP130 (Figures S2A, S2B, 2A, and 2B). Previously, it was reported that G144W mutant Membrin failed to localize to the *cis*-Golgi in a patient derived fibroblast line (Corbett et al., 2011). We therefore re-examined these cells with an experimentally validated anti-Membrin antibody (Figures S2C and S2D). Membrin could clearly be detected at the *cis*-Golgi of G144W mutant fibroblasts and did not appear to accumulate in the ER, confirming the above overexpression results in patient cells (Figures 2C, 2D, and S2E). We note that both Golgi-localized and overall Membrin levels were reduced in the single *GOSR2*-PME patient cell line (Figures S2F–S2H). However, there was also substantial variability in Membrin levels between healthy control lines (Figures S2F–S2H). Thus, from the above data, we conclude that both the G144W and K164del mutant forms of Membrin retain the capability to localize to the *cis*-Golgi target compartment. This suggests that the partial SNARE domain deficiencies found in liposome fusion assays are relevant to lipid bilayer fusion rates at the *cis*-Golgi.

OPEN
ACCESS
CellPress

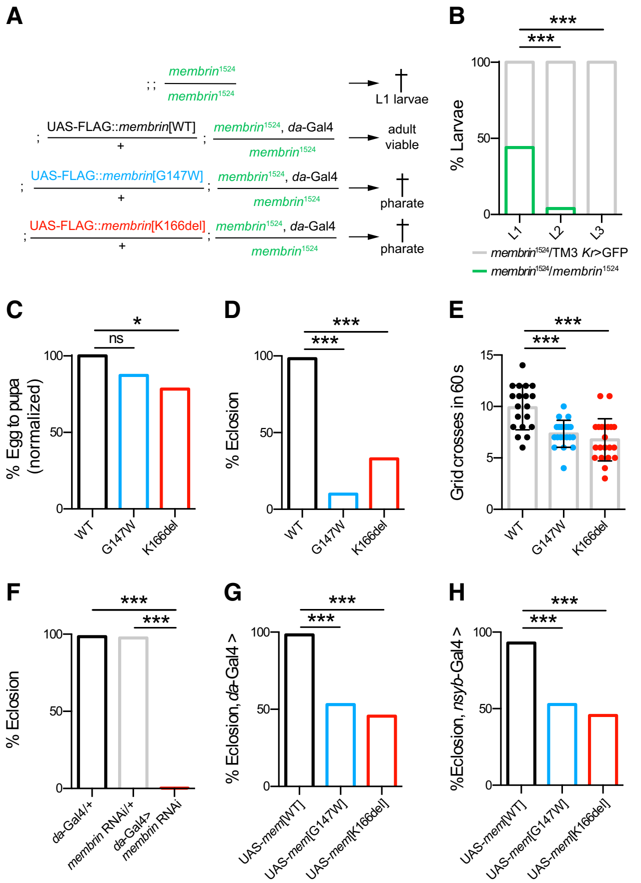

Figure 3. Membrin Mutations Cause Early Lethality and Locomotor Defects in *Drosophila*

(A) Genotypes of the *GOSR2*-PME *Drosophila* model used in this study. FLAG-tagged WT or mutant *membrin* (harboring the orthologous G147W/K166del mutations) is globally expressed via the *daughterless*-Gal4 driver in a *membrin*-null (*membrin*^{1524}) background. The shorthand Mem-WT, Mem-G147W, and Mem-K166del is used throughout the paper.

(B) The *membrin*^{1524} allele was balanced over the fluorescently labeled TM3 Kr > GFP chromosome to discern heterozygote animals. Homozygosity for *membrin*^{1524} caused largely L1 lethality, as at the L2 stage, hardly any non-GFP-positive larvae were detected. n = 50, 53, and 56 for L1, L2, and L3 larvae.

(C) Global expression of WT, G147W, and K166del mutant Membrin rescued *membrin*-null *Drosophila* to the pupal stage. Data are expressed relative to Mem-WT. n = 1,222, 1,308, and 1,260 eggs/embryos for Mem-WT/-G147W/-K166del.

(D) Mem-G147W and Mem-K166del *Drosophila* exhibited a drastic decrease in eclosion rates compared to Mem-WT. n = 120, 112, and 97 for Mem-WT/-G147W/-K166del non-*tubby* pupae.

(E) Freely moving Mem-G147W and Mem-K166del L3 larvae crossed fewer 4-mm grids in 60 s than Mem-WT larvae. n = 19, 20, and 21 for Mem-WT/-G147W/-K166del. Replicate values, mean, and SD are shown.

(F) Global *membrin* RNAi-mediated knockdown caused pharate adult stage lethality. n = 378, 162, and 313 for *da*-Gal4 driver and *membrin* RNAi transgene only controls and experimental knockdown.

(G) Global overexpression of mutant UAS-*membrin* in WT Membrin animals with *da*-Gal4 resulted in reduced eclosion. n = 284, 514, and 403 for UAS-*membrin*[WT]/[G147W]/[K166del].

(H) Neuronal overexpression of mutant UAS-*membrin* in WT Membrin animals with *nsyb*-Gal4 resulted in reduced eclosion. n = 616, 491, and 618 for UAS-*membrin*[WT]/[G147W]/[K166del].

*p < 0.05; **p < 0.01; ***p < 0.001; ns, not significant (p > 0.05); Fisher's exact test with Bonferroni correction (B–D and F–H) or one-way ANOVA with Dunnett's multiple comparison test (E).

Early Lethality and Locomotor Defects in *Drosophila* Models of *GOSR2*-PME

We next sought to study the effects of Membrin mutations in vivo using *Drosophila melanogaster*. Golgi SNARE proteins are highly conserved throughout evolution (Kloepper et al., 2007), and the *Drosophila* genome contains a single ortholog of the Membrin-encoding *GOSR2* gene (*membrin*, encoding the protein Membrin). Consistent with an essential role for Membrin orthologs in eukaryotes (Shim et al., 1991), homozygosity for the *membrin*-null allele *membrin*^{1524} resulted in lethality largely prior to the L2 larval stage (Figures 3A and 3B) (Ghabrial et al., 2011).

To assess the effects of *GOSR2*-PME mutations in *Drosophila*, we generated transgenic fly lines harboring FLAG-tagged WT or mutant (G144W or K164del) upstream activating sequence (UAS)-*GOSR2* transgenes. Each transgene sequence was integrated at the same genomic locus using site-specific ΦC31-mediated recombination to control for position effects on expression levels (Figure S3A) (Bischof et al., 2007). Expression of WT human *GOSR2* in a *membrin*-null background using the global *daughterless*-Gal4 driver fully rescued the lethality of *membrin*-null larvae and yielded adults that appeared morphologically normal (Figures S3B and S3C). While these adult animals exhibited severe motor impairments and usually died after 3 days, this result nonetheless demonstrates functional conservation between human and *Drosophila* Membrin, supporting the use of *Drosophila* to model *GOSR2*-PME.

Because neither mutant *GOSR2* transgene rescued *membrin*^{1524} animals to the L3 larval stage (data not shown), we next generated *GOSR2*-PME models that were closer to the normal physiology of *Drosophila*. Using an identical strategy, we created WT and mutant (G147W and K166del) *Drosophila* UAS-*membrin* transgenes and expressed them in a *membrin*-null genetic background. For simplicity, we term these mutant fly lines and their associated control Mem-G147W, Mem-K166del, and Mem-WT (Figure 3A).

In contrast to *membrin*-null flies, Mem-WT flies were viable to the adult stage (Figures 3C and 3D). Mem-G147W and Mem-K166del flies were viable to the pupal stage, surviving significantly longer than *membrin*-null animals (Figure 3C). However, Mem-G147W and Mem-K166del flies frequently died within the pupal cases as fully developed pharate adults (Figure 3D). When manually released from the pupal case, Mem-G147W and Mem-K166del adults appeared weak and uncoordinated. Mutant animals that successfully freed themselves from their pupal cases usually became quickly stuck in fly food and died within a few days. Mem-G147W and Mem-K166del L3 larvae also displayed significantly reduced rates of locomotion compared to Mem-WT larvae (Figure 3E). Locomotor deficits and lack of coordination may thus explain the frequent inability of adult Mem-G147W and Mem-K166del flies to escape from the pupal case, leading to early lethality.

Consistent with a partial loss-of-function disease mechanism conferred by the pathogenic *GOSR2*-PME mutations, reducing Membrin levels via transgenic RNAi also dramatically decreased

eclosion rates (Figure 3F). Co-overexpression of *membrin* RNAi with WT *Drosophila* UAS-*membrin* or WT human UAS-*GOSR2* transgenes resulted in a full rescue of eclosion deficits relative to a control transgene (UAS-*GCaMP6m*) (Figure S3D), confirming that the observed phenotype is specific to Membrin and further reinforcing the high degree of functional conservation between *Drosophila* and human Membrin.

Interestingly, global overexpression of UAS-*membrin*[G147W] and UAS-*membrin*[K166del] in WT flies similarly caused pharate adult lethality, albeit to a slightly weaker degree to that observed in the *GOSR2*-PME *Drosophila* model, where no endogenous Membrin is present (Figure 3G). This phenomenon is likely due to outcompetition of endogenous WT Membrin by the overexpressed mutant isoforms, and it suggested to us that overexpression of mutant Membrin could serve as a tool to test whether nervous system dysfunction is at the core of the observed *Drosophila* phenotypes. Indeed, overexpression of UAS-*membrin*[G147W] and UAS-*membrin*[K166del] selectively in neurons using two independent driver lines (*nsyb*-Gal4 and *elav*-Gal4) phenocopied the eclosion defects arising from global overexpression in WT flies (Figures 3H and S3E). Taken together, the incomplete rescue of *membrin*-null flies by G147W and K166del mutant Membrin provides in vivo evidence that these mutations cause partial loss of function, a postulate consistent with the above liposome fusion assays and supported by similar eclosion deficits due to Membrin knockdown. Furthermore, our overexpression data suggest that neuronal dysfunction is at the center of the observed organismal *Drosophila* phenotypes, in agreement with the almost exclusively neuronal phenotype of *GOSR2*-PME patients.

Profound Dendritic Growth Deficits in *GOSR2*-PME Model Neurons

The early onset of symptoms in *GOSR2*-PME suggests that Membrin mutations might alter aspects of neuronal development. Important clues toward the underlying mechanism stem from studies investigating neuronal consequences of mutations in other ER-to-Golgi trafficking proteins. Overexpression of GTP-locked Q71L-Arf1 in cultured hippocampal neurons leads to severely impaired dendritic growth (Horton et al., 2005). Similarly, Ye et al. (2007) found in a *Drosophila* forward genetic screen that mutations in Sec23, Sar1, and Rab1 cause dendritic growth deficiencies, likely by preventing ER-derived lipids and proteins from reaching the plasmalemma of growing neurons.

However, whereas Q71L-Arf1 and truncated Sar1 result in a substantial or complete block of anterograde trafficking (Dascher and Balch, 1994; Ye et al., 2007), the above liposome fusion assays and *Drosophila GOSR2*-PME models indicate that PME-causing mutations in Membrin involve a partial loss of function (Figures 1 and 3). Thus, we sought to test whether a partial decrease in ER-to-Golgi trafficking could impair dendritic growth. To do so, we genetically labeled ddaC sensory neurons within the larval body-wall with a membrane-tagged (and thus secretory-pathway-dependent) fluorophore (CD4::tdGFP) under control of the *ppk* promoter (Han et al., 2011). These neurons have highly complex, tiled dendritic arbors that branch in 2D and are unambiguously polarized into a single axon and multiple dendrites (Figure 4A, arrowhead indicates the axon). We imaged the same identifiable ddaC neuron in abdominal segment 5 of Mem-WT, Mem-G147W, and Mem-K166del L3 larvae (Figure 4A). Strikingly, both Mem-G147W and Mem-K166del larvae exhibited profound reductions in dendritic length and the number of terminal dendritic branches relative to Mem-WT (Figures 4B and 4C). Sholl analysis further revealed reduced elaboration of dendritic arbors and a significant reduction in dendritic intersections in Mem-G147W and Mem-K166del (Figures 4D and 4E). Using fluorescence recovery after photo-bleaching (FRAP), we tested whether trafficking of the CD4::tdGFP cargo was altered in Mem-G147W and Mem-K166del dendrites. Mem-K166del larvae exhibited a clear reduction in CD4::tdGFP FRAP and a similar albeit non-significant trend was seen for Mem-G147W (Figure 4F). Thus, dendritic growth and cargo trafficking within dendrites are impacted to a greater degree in Mem-K166del than Mem-G147W, consistent with the more severe SNARE defect of the orthologous D196del Bos1 mutation in liposome fusion assays (Figure 1).

Reduced Cargo Trafficking in Membrin Mutant Axons

While both growing dendrites and axons require abundant membrane addition (Aridor and Fish, 2009), Ye et al. (2007) suggested that axonal growth can be privileged in the face of ER-to-Golgi trafficking deficits. We thus examined axonal growth in *GOSR2*-PME *Drosophila* models. We detected axonal CD4::tdGFP signal in each ventral nerve cord (VNC) segmental nerve (Figure 5A; for saturated images, see Figure S4A), suggesting that at least one axon derived from the three *ppk*-positive neurons per hemisegment (ddaC, v'ada, and vdaB) reached its comparably distant target (Grueber et al., 2002). Compared with the reduced elaboration of ddaC dendrites (Figure 4), this is consistent with dendritic growth being more severely impaired than axonal growth by *GOSR2*-PME mutations. Nevertheless, steady-state CD4::tdGFP levels were significantly reduced in both the VNC and individual segmental nerves of Mem-G147W and Mem-K166del (Figures 5A–5C). Thus, a secretory pathway deficit is clearly present in distal axons and/or synapses of *membrin* mutant *Drosophila*. Using the large and experimentally accessible neuromuscular junction (NMJ) of L3 larvae, we next tested whether Membrin mutations also altered trafficking of endogenous synaptic cargos. We found robust reductions in the steady state levels of the synaptic vesicle protein cysteine string protein (CSP) in Mem-G147W and Mem-K166del synapses, while several other synaptic cargos were unaltered compared to Mem-WT (Figures S4B–S4H). These results provide proof of principle that Membrin mutations can affect the abundance of specific synaptic proteins. The different effects upon individual synaptic components at steady state may reflect varying trafficking demands of synaptic proteins due to differences in synaptic turnover.

Unimpaired Secretory Trafficking in G144W Membrin Mutant Fibroblasts

GOSR2-PME patients exhibit a restrictive neurological phenotype despite the ubiquitous expression of Membrin. We wondered whether the neuronal secretory pathway deficit observed in *GOSR2*-PME model axons might be the consequence of the unique geometry of neurons and thus not be applicable to a non-neuronal cell. To test this, we performed Golgi trafficking assays in a non-neuronal cell-type (primary

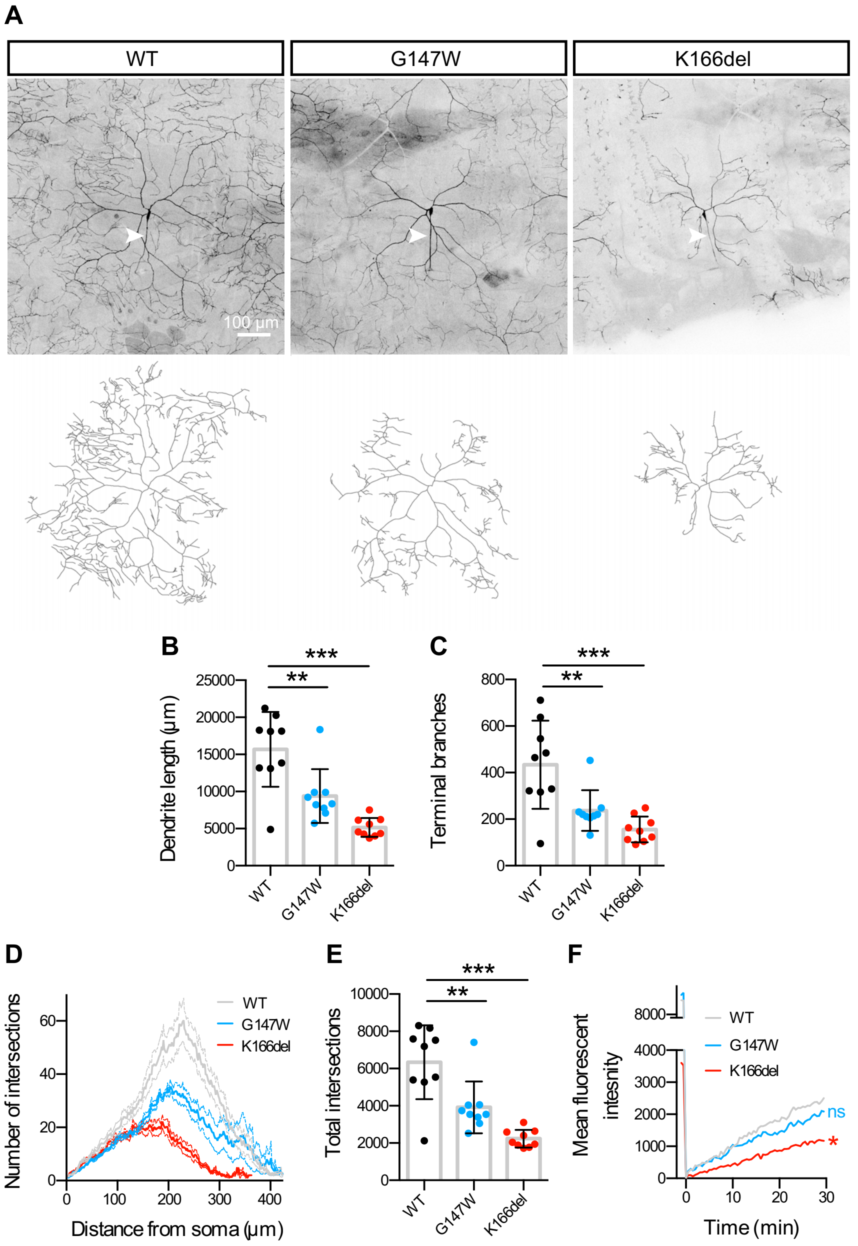

Figure 4. Membrin Mutations Cause Dendritic Growth Deficits

(A) Maximum intensity projections of ddaC abdominal segment 5 neurons genetically labeled with *ppk* > CD4::tdGFP in Mem-WT/-G147W/-K166del. Respective tracings of the dendritic arbors are shown below. Arrowheads indicate axons.

(B) Total dendritic length extracted from tracings as shown in (A). 9 A5 ddaC neurons per genotype were traced and analyzed in (B)–(E).

(C) Number of terminal branches of ddaC A5 neurons.

(D) Number of intersections of dendritic tracings with concentric circles with 2 pixel/circle increasing radii. Mean ± SEM are shown.

(E) Total intersection of Sholl analysis as shown in (D).

(F) CD4::tdGFP in large segments of primary ddaC A5 dendrites adjacent to the soma were photobleached with a 50 μm^2 region of interest and fluorescence recovery quantified 25 µm from the soma proximal bleach margin. Means of n = 9, 8, and 9 ddaC neurons for Mem-WT/-G147W/-K166del are shown. Asterisks and ns indicate endpoint comparison after 29.5-min recovery.

Replicate values, mean, and SD are shown unless otherwise stated. *$p < 0.05$; **$p < 0.01$; ***$p < 0.001$; ns, not significant ($p > 0.05$); one-way ANOVA with Dunnett's multiple comparison test.

skin fibroblasts) derived from a G144W *GOSR2*-PME patient and healthy controls. In this cell-type, we overexpressed human growth hormone (hGH) fused to four FM domains and a Halo tag. The FM domains self-aggregate in the absence of a solubilizing drug and thus do not allow ER exit of hGH (Figure S5A, first column) (Rivera et al., 2000). Addition of D/D solubilizer disaggregates the FM domains and allows the cargo to enter the secretory pathway. By 10 min after solubilization, we detected significant colocalization of tagged hGH with the *cis*-Golgi marker GM130, which decreased by 20 and 30 min as the cargo exited this compartment (Figures S5A and S5B). Remarkably, trafficking kinetics in G144W mutant Membrin fibroblasts were almost indistinguishable from controls (Figures S5A and S5B). This finding supports the concept that cellular effects of the partial loss-of-function G144W Membrin mutation are unmasked only under large secretory pathway requirements, suggesting why only the nervous system with its high trafficking demands is symptomatically affected in *GOSR2*-PME.

Presynaptic Morphological Defects in *GOSR2*-PME Model Neurons

Because we could detect secretory pathway defects in distal axons and synapses of *GOSR2*-PME *Drosophila*, we next investigated whether synaptic integrity might be altered due to the *GOSR2*-PME mutations. We thus examined presynaptic morphology at the L3 larval NMJ by labeling neuronal membranes with an anti-horseradish peroxidase (HRP) antibody. Motor neurons of Mem-WT, Mem-G147W, and Mem-K166del flies successfully formed synapses, and Mem-G147W and Mem-K166del synapses did not exhibit significant reductions in bouton number or size relative to Mem-WT (Figures 5D, 5E, and S6A–S6C). However, we observed two clear morphological abnormalities in Mem-G147W and Mem-K166del synapses. First, terminal synaptic boutons often exhibited axonal protrusions lacking normal rounded boutons, which were less common and shorter in Mem-WT synapses (Figures 5D, 5F, S6C, and S6D). Second, we observed small boutons that were disconnected from the main axonal branch (Figure 5D,

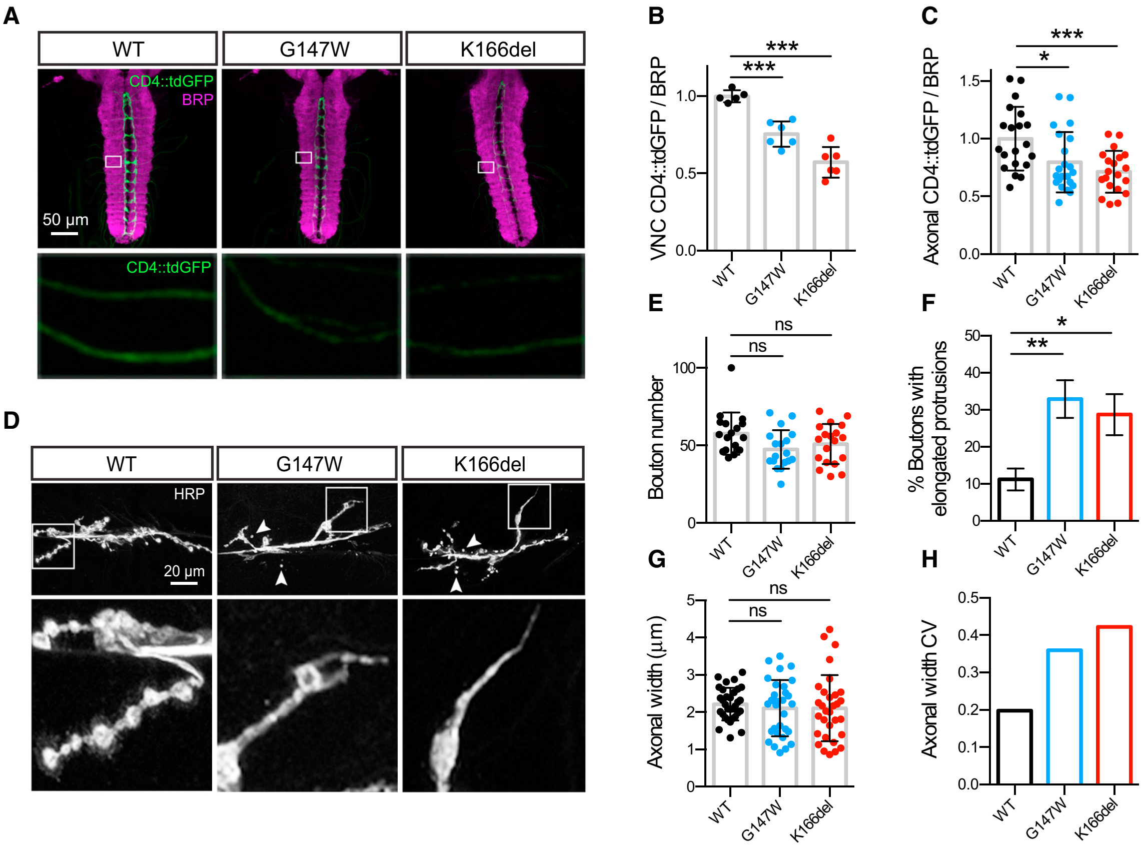

Figure 5. Membrin Mutations Alter Presynaptic Morphology and Axonal Stereotypy

(A) Top: confocal z-stacks showing projections from *ppk*-positive sensory neurons labeled with membrane-tagged CD4::tdGFP innervating the ventral nerve cord (VNC) of L3 larvae. Synaptic neuropil of the VNC is labeled with anti-BRP. Below: magnified regions of segmental nerves.

(B and C) Quantification of CD4::tdGFP fluorescence in the VNC neuropil (B) or in segmental nerves (C) normalized to BRP and expressed relative to Mem-WT. n = 5, 5, and 6 (B) and n = 20, 22, and 20 (C) for Mem-WT/-G147W/-K166del.

(D) Top: example confocal z-stacks of HRP-labeled motor neurons innervating muscle 6/7, segment 3 of L3 larvae. Arrowheads point to small, isolated boutons that appear unattached to the axon. Below: magnified terminal boutons. In contrast to the rounded morphology in Mem-WT larvae, terminal boutons in Mem-G147W and Mem-K166del larvae often exhibit elongated protrusions. Further examples are depicted in Figure S6C.

(E) Average number of boutons (type 1b and 1s) at muscle 6/7, segment 3. n = 18, 18, and 19 for Mem-WT/-G147W/-K166del.

(F) Percentage of terminal boutons with elongated protrusions. Mean and SEM are shown. n = 32, 31, and 31 for Mem-WT/-G147W/-K166del.

(G) Maximal axonal width of motor neurons innervating muscle 6/7, segment 3. n = 32, 30, and 31 for Mem-WT/-G147W/-K166del.

(H) Coefficient of variation (calculated as SD/mean) in axonal width for each genotype.

Replicate values, mean and SD are shown unless otherwise stated. $^{*}p < 0.05$; $^{**}p < 0.01$; $^{***}p < 0.001$; ns, not significant ($p > 0.05$); one-way ANOVA with Dunnett's multiple comparison test (B and C) or Kruskal-Wallis test with Dunn's post hoc test (E–G).

arrowheads). In addition, analysis of axonal diameter revealed a significant increase in the variability of the maximal axonal diameter in Mem-G147W and Mem-K166del NMJs (Figure 5G), as measured by the coefficient of variation (Figure 5H) and F-test (Mem-G147W versus Mem-WT, p = 0.0035; Mem-K166del versus Mem-WT, p = 0.0002). Thus, partial reductions in secretory pathway trafficking result in multifaceted abnormalities in motor neuron synapse development and impact the stereotypy of terminal axon morphology.

Membrin Mutations Result in Synaptic Retraction and Cytoskeletal Fragmentation

To examine the effects of Membrin mutations on synaptic structure in more detail, we co-stained Mem-WT, Mem-G147W, and Mem-K166del synapses with antibodies against the presynaptic active zone marker Bruchpilot (BRP) and postsynaptic GLURIII glutamate receptors (Figure 6A). BRP localized to Mem-G147W and Mem-K166del NMJs in amounts comparable to Mem-WT (Figure 6B). However, dual pre- and postsynaptic labeling revealed pronounced strings of small presynaptic boutons in Mem-G147W and Mem-K166del synapses in which GLURIII was no longer opposed by BRP (Figures 6A and 6C). This disruption of transsynaptic organization is indicative of presynaptic retraction, where synaptic connections initially form but fail to be maintained throughout development (Eaton et al., 2002; Pielage et al., 2008).

Synaptic retraction can be induced by mutations in cytoskeletal proteins (Koch et al., 2008; Pielage et al., 2008; 2011,

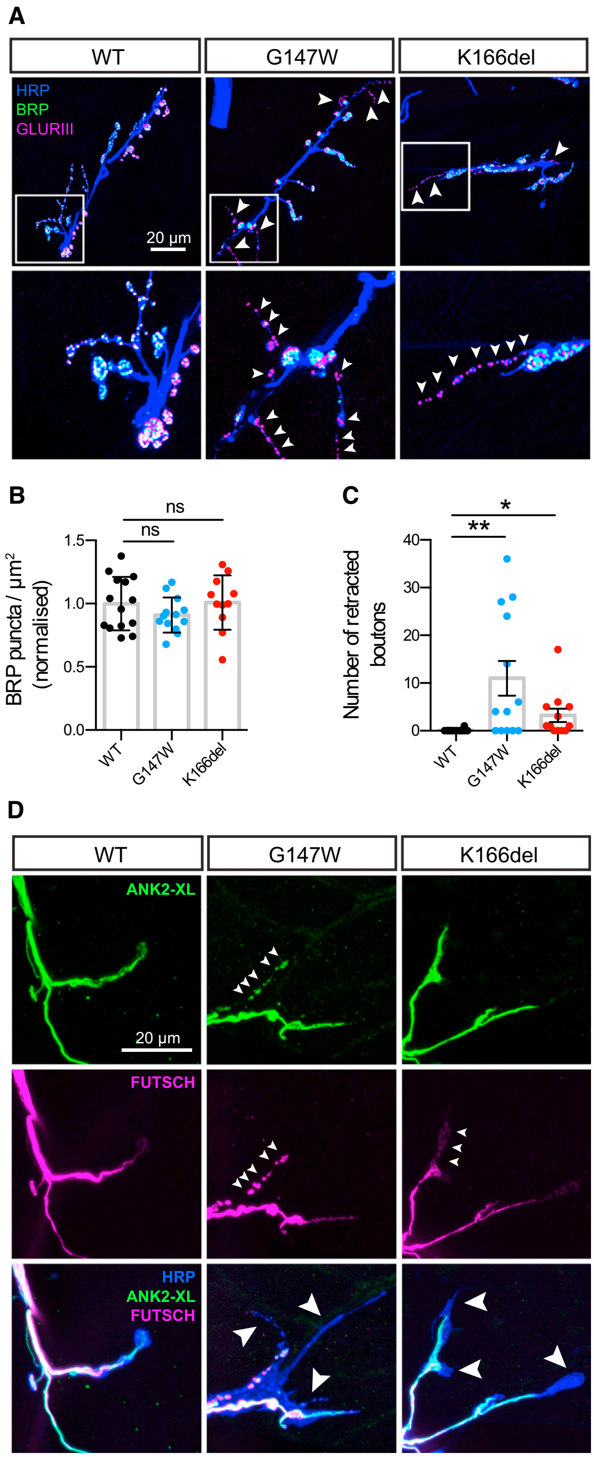

Figure 6. Synaptic Retraction and Presynaptic Cytoskeletal Fragmentation in *membrin* Mutants

(A) Top: Maximum intensity z projection of confocal stacks showing pre- and postsynaptic apposition between BRP-labeled active zones and postsynaptic GLURIII glutamate receptors. Arrowheads denote regions where glutamate receptors lack their presynaptic active zone counterparts. Below: magnified view of regions exhibiting loss of BRP-labeled active zones in Mem-G147W and Mem-K166del synapses.

(B) Normalized density of BRP puncta per NMJ area. n = 14, 13, and 11 for Mem-WT/-G147W/-K166del. Replicate values, mean, and SD are shown.

(C) Average number of synaptic boutons where BRP fails to oppose GLURIII. n = 14, 13, and 12 for Mem-WT/-G147W/-K166del. Replicate values, mean, and SEM are shown.

(D) Confocal z-stack maximum intensity projections illustrating localization of Ankyrin-2-XL (ANK2-XL) and Futsch. Small arrowheads point to synaptic domains containing either fragmented Futsch and ANK2-XL or reduced amounts of Futsch. Large arrowheads point to synaptic boutons and elongated protrusion apparently lacking both Futsch and ANK2-XL.

*p < 0.05; **p < 0.01; ns, not significant (p > 0.05); Kruskal-Wallis test with Dunn's post hoc test.

2005). Long protrusions lacking specialized boutons and alterations in axonal diameter further suggested cytoskeletal defects in Mem-G147W and Mem-K166del synapses (Pielage et al., 2011; Stephan et al., 2015). Hence, we examined the localization of two presynaptic cytoskeletal proteins: Futsch (a microtubule-binding protein) and Ankyrin-2-XL (ANK2-XL) (Koch et al., 2008; Roos et al., 2000). In Mem-WT synapses, both Futsch and ANK2-XL were co-localized in central and distal axons and invaded terminal boutons (Figure 6D) (Stephan et al., 2015). Strikingly, in both terminal boutons and elongated protrusions of Mem-G147W and Mem-K166del synapses, we observed either fragmentation of the normally continuous Futsch- and ANK2-XL-labeled cytoskeleton or an absence of one or both proteins (Figure 6D). Thus, secretory defects due to Membrin mutations reduce the local integrity of the presynaptic cytoskeleton.

Physiological Abnormalities at Membrin Mutant Synapses

We next asked whether Membrin mutations altered spontaneous or evoked neurotransmitter release at the L3 larval NMJ. We detected a clear reduction in the frequency of spontaneous miniature excitatory postsynaptic potentials (mEPSPs) in Mem-G147W and Mem-K166del flies (Figures 7A and 7B), while the amplitude and time course of mEPSPs were comparable between WT and Membrin mutants (Figures S7A and S7B). No effect of Mem-G147W and Mem-K166del mutations on the amplitude of single postsynaptic evoked EPSPs was observed (Figure S7C). However, we often observed grossly deformed trains of EPSPs in both Mem-G147W and Mem-K166del following 5 consecutive stimuli at 10 Hz, where between one and all five EPSPs exhibited broader waveforms with multiple peaks and occasional merging of EPSPs (Figure 7C). Significantly more EPSP trains were abnormal in both mutants compared to Mem-WT: 5% of EPSP trains in Mem-WT were scored abnormal by a blinded observer, compared to ∼25% in Mem-G147W and 22% in Mem-K166del (Figure 7D). In addition, the area under the EPSP train was robustly increased in Mem-G147W and Mem-K166del compared to Mem-WT (Mem-WT: 9013 mVs ± 850.5 (mean ± SEM); Mem-G147W: 15,226 ± 1,706; Mem-K166del: 15,992 ± 2,298) (Figure 7E).

Finally, given that *GOSR2*-PME is an epilepsy syndrome, we tested whether such neuronal hyperactivity and the dendritic/synaptic morphological abnormalities resulted in seizure-like neuronal activity. To do so, we electrically induced seizures in Mem-WT and *GOSR2*-PME model L3 larvae and measured the

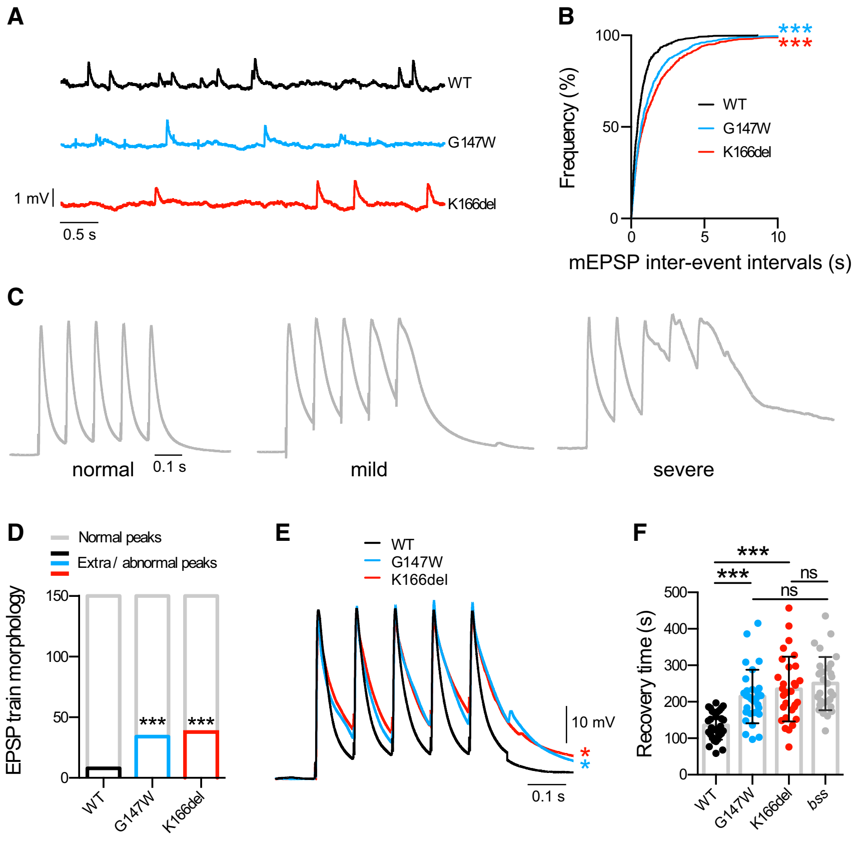

Figure 7. Physiological Abnormalities at *membrin* Mutant NMJs

(A) Representative traces of miniature excitatory postsynaptic potentials (mEPSPs) recorded from Mem-WT/-G147W/-K166del L3 larval muscle 6 abdominal segments 2–4.

(B) Cumulative frequency plot of mEPSP intervals. 800 events per genotype from 8 animals each are shown.

(C) Illustrative traces depicting mild to severe EPSP waveform distortion following 5 stimuli at 10 Hz. Traces are normalized to the peak amplitude.

(D) Analysis of total number of abnormal events as a result of 10 Hz stimulation. 15 events were analyzed from each recording from 10 animals per genotype.

(E) Overlay of averaged 10 Hz EPSP trains illustrating a significantly larger mean area under the curve in Mem-G147W and Mem-K166del compared to Mem-WT larvae (n = 10).

(F) Mem-G147W and Mem-K166del larvae displayed longer recovery times after CNS electroshock compared to Mem-WT, indicative of increased seizure severity of the *GOSR2*-PME models. Mem-G147W and Mem-K166del recovery times were not significantly different compared to the *Drosophila* seizure model *bang-senseless* (*bss*). Replicate values, mean and SD are shown. n = 30 for Mem-WT/-G147W/-K166del/*bss*.

*$p < 0.05$; ***$p < 0.001$; Kolmogorov-Smirnov test and Bonferroni correction (B), Fisher's exact test and Bonferroni correction (D), one-way ANOVA with Dunnett's multiple comparison test (E), and Kruskal-Wallis test with Dunn's post hoc test (F).

recovery time required to resume normal locomotor behavior (Giachello and Baines, 2015). Remarkably, Mem-G147W and Mem-K166del displayed a significant increase in duration of seizure-like activity when compared to Mem-WT (Figure 7F). The effect sizes of our *GOSR2*-PME models were comparable to the widely studied *bang-senseless Drosophila* seizure model (Figure 7F) (Parker et al., 2011). Thus, our findings indicate that pathogenic *membrin* mutations cause not only dendritic and synaptic morphological abnormalities but also altered synaptic function, which collectively impact the nervous system in a way to give rise to locomotor defects and hyperexcitability.

DISCUSSION

To date, how mutations in Membrin, a ubiquitous and essential Golgi SNARE protein, manifest as a disorder restricted to the nervous system has been unclear. Here, we demonstrate that PME-causing Membrin mutations partially reduce SNARE activity yet still result in profound dendritic growth deficits in *Drosophila* models. Furthermore, Membrin mutations cause synaptic disassembly and altered neurotransmission, establishing a close dependence of synaptic stability and physiology upon precisely tuned secretory trafficking.

Our findings reinforce a previous *Drosophila* screen identifying the ER-to-Golgi trafficking proteins Sar1, Sec23, and Rab1 to be required for dendrite growth (Ye et al., 2007) and provide an instance that highlights the potential clinical relevance of this pathway (Jan and Jan, 2010). Interestingly, mutations in Sec23A, Sec23B, Sec24D, and Sar1b present in humans with largely non-neuronal clinical phenotypes of cranio-lenticulo-sutural dysplasia, congenital dyserythropoietic anemia, a syndromic form of osteogenesis imperfecta and lipid absorption disorders (Boyadjiev et al., 2006; Garbes et al., 2015; Jones et al., 2003; Schwarz et al., 2009). This appears to be a consequence of tissue-specific differential utilization of the two available isoforms of Sec23 and Sar1 and special demands upon the COPII coat due to the large size of procollagen and chylomicrons (Garbes et al., 2015; Fromme et al., 2007; Jones et al., 2003).

Our results suggest that subtle defects in the secretory pathway can be highly relevant for neuronal growth while not robustly affecting a non-neuronal cell type such as a fibroblast, supporting the postulate that due to the unique plasma membrane demands of a growing neuron, minor disruption of the secretory pathway can disrupt the nervous system while falling below a critical threshold in other organs (Pfenninger, 2009). While we cannot fully rule out a contribution to *GOSR2*-PME symptoms from non-neuronal cells, we hypothesize that this is the likeliest explanation for why *GOSR2*-PME symptoms selectively involve nervous system dysfunction. Furthermore, the observed dendritic growth deficits may explain one hallmark of this disorder—lack of coordination—since reduced membrane transport may most severely impact neurons with

highly elaborate dendritic arbors such as cerebellar Purkinje cells (Ramón y Cajal, 1906). Such impairment would likely give rise to ataxia, as Purkinje cells are critically important for motor coordination (Kasumu and Bezprozvanny, 2012). Interestingly, cerebellar defects have been suggested to be involved in the pathogenesis of cortical myoclonus (Ganos et al., 2014).

Our study extends previous findings by showing that early secretory pathway changes can also impact presynaptic morphology and physiology as well as dendritogenesis (Ye et al., 2007). We found that larval NMJs of Mem-G147W and Mem-K166del exhibit synaptic retraction, abnormal elongated protrusions lacking synaptic specializations, reduced spontaneous neurotransmitter release, and malformed EPSPs.

Membrin acts as a gatekeeper at the *cis*-Golgi and likely determines the trafficking rates of a plethora of synaptic and axonal proteins. Thus, we speculate that the observed synaptic changes arise from a complex interaction of trafficking delays or steady-state reductions of many cargos. Nevertheless, we identify several molecular correlates of the above structural changes. These include the loss or fragmentation of the cytoskeletal proteins ANK2-XL (an Ankyrin-2 isoform) and Futsch, particularly in presynaptic boutons with elongated protrusions. The axonal and synaptic cytoskeleton contains several interlinked constituents, including a microtubule core, actin filaments, and a submembranous mesh of Ankyrin and Spectrin (Goellner and Aberle, 2012). These components regulate an array of neurodevelopmental and physiological parameters, including synaptic growth, morphology, and stability; axonal caliber; and ion channel localization (Goellner and Aberle, 2012; Stephan et al., 2015). Futsch is the *Drosophila* homolog of the mammalian microtubule-binding protein MAP1B (Roos et al., 2000), and the absence or fragmentation of Futsch in Mem-G147W and Mem-K166del synapses implies similar alterations in microtubule stability. Given the synaptic retraction observed in Mem-G147W and Mem-K166del NMJs, it is interesting to note that destabilization of microtubules is an early event during naturally occurring synapse loss at the mammalian NMJ (Bishop et al., 2004; Brill et al., 2016). Ankyrin-2 isoforms delineate *Drosophila* synaptic termini into rounded boutons separated by thin inter-bouton domains (Koch et al., 2008; Pielage et al., 2008). Since Mem-G147W and Mem-K166del boutons often exhibit elongated protrusions reminiscent of extended inter-bouton domains, we speculate direct links among local destabilization of the synaptic cytoskeleton, synaptic retraction, and the presence of elongated protrusions in Mem-G147W and Mem-K166del synapses. Interestingly, EMG evidence of motoneuron denervation in *GOSR2*-PME patients has been reported, and the typical absence of deep-tendon reflexes in this disorder might be a consequence of analogous changes (van Egmond et al., 2014).

Membrin mutations not only affect synaptic structure but also disrupt evoked and spontaneous neurotransmitter release. Again, the underlying mechanisms are likely to represent a complex, cumulative process caused by insufficient trafficking of diverse ion channels and vesicle release proteins. Increased variability of axonal diameters in Mem-G147W and Mem-K166del may further alter action potential propagation velocities across different axons innervating the same muscle, leading to desynchronized depolarizing currents.

Our work raises the possibility that other PME subtypes might share cellular pathways and/or neural circuits with *GOSR2*-PME. For instance, mutations in *PRICKLE1* cause PME in humans and seizures in flies (Bassuk et al., 2008; Tao et al., 2011), and *PRICKLE1* has been linked to neurite growth and axonal trafficking (Ehaideb et al., 2014; Liu et al., 2013). PME-linked mutations in the potassium channel gene *KCNC1* are thought to mainly impair fast-spiking neurons (Muona et al., 2015). Such a preferential defect in high-frequency firing neurons is also conceivable in *GOSR2*-PME, where EPSP abnormalities are more pronounced under repetitive stimulation.

In summary, by elucidating the pathophysiology of *GOSR2*-PME, we identify a critical role for Membrin in promoting synaptic integrity, highlight stringent requirements of dendritic growth on the secretory pathway, and define how mutations in an essential gene can selectively disrupt nervous system function.

EXPERIMENTAL PROCEDURES

Molecular Biology and Bioinformatics

Human *GOSR2* CDS as well as *Drosophila melanogaster membrin* CDS with and without the G144W/G147W and K164del/K166del mutations preceded by 5′ FLAG-tag coding sequence were custom synthesized by GeneArt (Thermo Fisher Scientific) and subsequently cloned via NotI and KpnI (NEB) into pUASTattB, giving rise to pUASTattB_FLAG::*GOSR2*[WT]/pUASTattB_FLAG::*membrin*[WT], pUASTattB_FLAG::*GOSR2*[G144W]/pUASTattB_FLAG::*membrin*[G147W], and pUASTattB_FLAG::*GOSR2*[K164del]/pUASTattB_FLAG::*membrin*[K166del]. See Supplemental Experimental Procedures for further details.

Liposome Fusion Assays

The recombinant yeast Golgi SNARE proteins were expressed and purified in *E. coli* BL21 (DE3) cells as described previously (Parlati et al., 2000). Purified SNARE proteins were reconstituted into lipid vesicles using the detergent (1% n-octyl-β-D-glucopyranoside) dilution and dialysis method (Weber et al., 1998). See Supplemental Experimental Procedures for further details.

Cell Culture and Transfections

Primary skin derived fibroblasts from the first described *GOSR2*-PME patient were kindly shared by Mark Corbett (Corbett et al., 2011). As controls, we used fibroblasts from healthy individuals of either the same sex and similar age or the opposite sex and divergent age (control 1, 23-year-old female; control 2, 60-year-old male [at time of biopsy]). See Supplemental Experimental Procedures for further details.

Fibroblast Imaging

For immunofluorescence studies, cells were seeded on #1.5 glass coverslips, fixed with 4% paraformaldehyde (PFA), and permeabilized in PBS containing Triton X-100 and NP40. After primary and secondary antibody incubation steps, coverslips were mounted in SlowFade Gold Antifade (Thermo Fisher Scientific). See Supplemental Experimental Procedures for further details.

Western Blot

Cells were lysed in 20 mM HEPES (pH 7.5), 100 mM KCl, 5% glycerol, 10 mM EDTA, and 1% Triton X-100 supplemented with phosphatase and proteinase inhibitors (PhosSTOP/cOmplete, Roche). Total protein content was quantified with the Pierce 660 nm assay (Thermo Fisher Scientific) and equal amounts loaded into each lane. Proteins were separated on a 4%–10% Bis-Tris polyacrylamide gel (Thermo Fisher Scientific) and transferred onto polyvinylidene fluoride (PVDF) membranes (EMD Millipore). See Supplemental Experimental Procedures for further details.

Drosophila Genetics and Phenotyping

membrin[1,524] flies were previously generated in an ethyl methanesulfonate (EMS) screen and kindly shared by Mark Krasnow (Ghabrial et al., 2011). This strain harbors a premature stop codon upstream of the *membrin* SNARE domain encoding sequence and therefore represents a null allele. To control for potential genetic background effects, we outcrossed *membrin*[1,524] for five generations into an isogenic iso31 background by following an AccI (NEB) restriction site that is introduced by the nonsense mutation. *daughterless*-Gal4 (#55850), UAS-*GCaMP6m* (#42748), and *nsyb*-Gal4 (#51635) flies were obtained from the Bloomington Stock Center, and the *membrin* RNAi transgene was obtained from the Vienna *Drosophila* Resource Center (VDRC) (GD 44535) (Dietzl et al., 2007). These transgenes and alleles were backcrossed for 5 generations into an isogenic iso31 background. Backcrossed *elav*-Gal4 flies were a kind gift from Kyunghee Koh. Flies were reared on a standard cornmeal-molasses-yeast medium at 25°C in a 12-hr light/dark cycle. See Supplemental Experimental Procedures for further details.

Dendritic Analysis

The highly elaborate ddaC neurons in abdominal segment 5 of L3 larvae were used throughout (Grueber et al., 2002). For morphological analysis, larvae were heat-killed and mounted under a #1.5 glass coverslip. z stacks of ddaC neurons were obtained with Zeiss confocal LSM710 microscopes with a N-Achroplan 10× 0.25 numerical aperture (NA) objective to capture the entire arbor. See Supplemental Experimental Proceduresfor further details.

Immunohistochemistry of Larval Neuromuscular Junctions and Brains

When examining synaptic development at the larval NMJ, synapses innervating muscle 6/7 of segment 3 were imaged on a Zeiss confocal LSM710 with either a Plan-Apochromat 20× 0.8 NA or a Plan-Apochromat 63× 1.4 NA oil-immersion objective. See Supplemental Experimental Procedures for further details.

NMJ Electrophysiology and Larval Seizure Assay

Wandering L3 larvae were dissected in ice-cold, Ca^{2+}-free HL3-like solution (70 mM NaCl, 5 mM KCl, 10 mM $NaHCO_3$, 115 mM sucrose, 5 mM trehalose, 5 mM HEPES, and 10 mM $MgCl_2$). Motor nerves were severed just below the VNC, and the brain was removed. $CaCl_2$ (1 mM) was added to the bath solution for intracellular recording from muscle 6 of abdominal segments 2–4. Sharp microelectrodes (thick-walled borosilicate glass capillaries, pulled on a Sutter Flaming/Brown P-97 micropipette puller) were filled with 3 M KCl and had resistances of 20–30 MΩ. For recording of stimulus evoked excitatory postsynaptic potentials (EPSPs), severed nerves were drawn into a thin-walled glass-stimulating pipette and stimulated with square-wave voltage pulses (0.1 ms, 10 V, A-M Systems Model 2100 Isolated Pulse Simulator). EPSPs and spontaneously occurring mEPSPs were recorded at a controlled room temperature of 22°C –25°C with a Geneclamp 500 amplifier (Axon Instruments) and were further amplified with an LHBF-48x amplifier (NPI Electronic). Wandering third-instar larvae were electroshocked as previously described (Giachello and Baines, 2015). See Supplemental Experimental Procedures for further details.

Statistical Comparisons

Statistical analyses were performed using GraphPad Prism. Tests used to compare control and experimental populations are detailed in the figure legends.

SUPPLEMENTAL INFORMATION

Supplemental Information includes Supplemental Experimental Procedures and seven figures and can be found with this article online at https://doi.org/10.1016/j.celrep.2017.09.004.

AUTHOR CONTRIBUTIONS

Conceptualization, R.P., S.S.K., J.E.R., and J.E.C.J.; Methodology, R.P., N.T.M., S.S.K., and J.E.C.J.; Formal Analysis, R.P., S.A.L., N.T.M., C.N.G.G., J.E.C.J.; Investigation, R.P., S.A.L., N.T.M., C.N.G.G., N.P., J.E.C.J.; Resources, H.H., D.M.K., R.A.B., M.M.U., J.J.L.H., J.E.R., and J.E.C.J.; Writing – Original Draft, R.P. and J.E.C.J.; Writing – Review & Editing, R.P., S.A.L., N.T.M., C.N.G.G., D.M.K., R.A.B., M.M.U., S.S.K., J.J.L.H., J.E.C.J.; Visualization, R.P. and J.E.C.J.; Supervision, H.H., D.M.K., R.A.B., M.M.U., S.S.K., J.J.L.H., J.E.R., and J.E.C.J.; Project Administration, R.P. and J.E.C.J.; Funding Acquisition, R.P., H.H., D.M.K., R.A.B., M.M.U., J.J.L.H., J.E.R., and J.E.C.J.

ACKNOWLEDGMENTS

We would like to thank Andreas Ernst for experimental suggestions and discussion of data, Mark Corbett for kindly sharing G144W-Membrin mutant fibroblasts; Mark Krasnow for providing *membrin*[1,524] flies; Lily Yeh Jan and Yuh-Nung Jan for sharing *ppk* > CD4::tdGFP flies; Herman Aberle, David Deitcher, and Aaron DiAntonio for sharing antibodies; and Giampietro Schiavo, Helene Plun-Favreau, and Stephanie Schorge for helpful comments on the manuscript. Antibodies from the Developmental Studies Hybridoma Bank (created by the NICHD of the NIH and maintained at The University of Iowa) and flies from Bloomington Stock Center (NIH P40OD018537) and the VDRC were used in this study. This work was funded by the Wellcome Trust (Synaptopathies strategic award 104033 to J.E.C.J., J.E.R., H.H., and D.M.K.), the MRC (MR/P012256/1 to J.E.C.J), the BBSRC (BB/J017221/1 to J.J.L.H. and BB/N/014561/1 to R.A.B.), and the NIHR (funding for UCL/UCLH Biomedical Research Centre). R.P. is supported by a Brain Research Trust PhD studentship. S.A.L. is supported by a BBSRC DTP studentship.

Received: June 5, 2017
Revised: August 17, 2017
Accepted: September 1, 2017
Published: October 3, 2017

REFERENCES

Aridor, M., and Fish, K.N. (2009). Selective targeting of ER exit sites supports axon development. Traffic *10*, 1669–1684.

Bassuk, A.G., Wallace, R.H., Buhr, A., Buller, A.R., Afawi, Z., Shimojo, M., Miyata, S., Chen, S., Gonzalez-Alegre, P., Griesbach, H.L., et al. (2008). A homozygous mutation in human *PRICKLE1* causes an autosomal-recessive progressive myoclonus epilepsy-ataxia syndrome. Am. J. Hum. Genet. *83*, 572–581.

Bischof, J., Maeda, R.K., Hediger, M., Karch, F., and Basler, K. (2007). An optimized transgenesis system for *Drosophila* using germ-line-specific phiC31 integrases. Proc. Natl. Acad. Sci. USA *104*, 3312–3317.

Bishop, D.L., Misgeld, T., Walsh, M.K., Gan, W.-B., and Lichtman, J.W. (2004). Axon branch removal at developing synapses by axosome shedding. Neuron *44*, 651–661.

Boissé Lomax, L., Bayly, M.A., Hjalgrim, H., Møller, R.S., Vlaar, A.M., Aaberg, K.M., Marquardt, I., Gandolfo, L.C., Willemsen, M., Kamsteeg, E.-J., et al. (2013). 'North Sea' progressive myoclonus epilepsy: phenotype of subjects with *GOSR2* mutation. Brain *136*, 1146–1154.

Boyadjiev, S.A., Fromme, J.C., Ben, J., Chong, S.S., Nauta, C., Hur, D.J., Zhang, G., Hamamoto, S., Schekman, R., Ravazzola, M., et al. (2006). Cranio-lenticulo-sutural dysplasia is caused by a *SEC23A* mutation leading to abnormal endoplasmic-reticulum-to-Golgi trafficking. Nat. Genet. *38*, 1192–1197.

Brill, M.S., Kleele, T., Ruschkies, L., Wang, M., Marahori, N.A., Reuter, M.S., Hausrat, T.J., Weigand, E., Fisher, M., Ahles, A., et al. (2016). Branch-Specific Microtubule Destabilization Mediates Axon Branch Loss during Neuromuscular Synapse Elimination. Neuron *92*, 845–856.

Corbett, M.A., Schwake, M., Bahlo, M., Dibbens, L.M., Lin, M., Gandolfo, L.C., Vears, D.F., O'Sullivan, J.D., Robertson, T., Bayly, M.A., et al. (2011). A mutation in the Golgi Qb-SNARE gene *GOSR2* causes progressive myoclonus epilepsy with early ataxia. Am. J. Hum. Genet. *88*, 657–663.

Dascher, C., and Balch, W.E. (1994). Dominant inhibitory mutants of ARF1 block endoplasmic reticulum to Golgi transport and trigger disassembly of the Golgi apparatus. J. Biol. Chem. *269*, 1437–1448.

Dietzl, G., Chen, D., Schnorrer, F., Su, K.-C., Barinova, Y., Fellner, M., Gasser, B., Kinsey, K., Oppel, S., Scheiblauer, S., et al. (2007). A genome-wide transgenic RNAi library for conditional gene inactivation in *Drosophila*. Nature *448*, 151–156.

Eaton, B.A., Fetter, R.D., and Davis, G.W. (2002). Dynactin is necessary for synapse stabilization. Neuron *34*, 729–741.

Ehaideb, S.N., Iyengar, A., Ueda, A., Iacobucci, G.J., Cranston, C., Bassuk, A.G., Gubb, D., Axelrod, J.D., Gunawardena, S., Wu, C.-F., and Manak, J.R. (2014). *prickle* modulates microtubule polarity and axonal transport to ameliorate seizures in flies. Proc. Natl. Acad. Sci. USA *111*, 11187–11192.

Fischer von Mollard, G., and Stevens, T.H. (1998). A human homolog can functionally replace the yeast vesicle-associated SNARE Vti1p in two vesicle transport pathways. J. Biol. Chem. *273*, 2624–2630.

Fromme, J.C., Ravazzola, M., Hamamoto, S., Al-Balwi, M., Eyaid, W., Boyadjiev, S.A., Cosson, P., Schekman, R., and Orci, L. (2007). The genetic basis of a craniofacial disease provides insight into COPII coat assembly. Dev. Cell *13*, 623–634.

Ganos, C., Kassavetis, P., Erro, R., Edwards, M.J., Rothwell, J., and Bhatia, K.P. (2014). The role of the cerebellum in the pathogenesis of cortical myoclonus. Mov. Disord. *29*, 437–443.

Gao, Y., Zorman, S., Gundersen, G., Xi, Z., Ma, L., Sirinakis, G., Rothman, J.E., and Zhang, Y. (2012). Single reconstituted neuronal SNARE complexes zipper in three distinct stages. Science *337*, 1340–1343.

Garbes, L., Kim, K., Rieß, A., Hoyer-Kuhn, H., Beleggia, F., Bevot, A., Kim, M.J., Huh, Y.H., Kweon, H.-S., Savarirayan, R., et al. (2015). Mutations in *SEC24D*, encoding a component of the COPII machinery, cause a syndromic form of osteogenesis imperfecta. Am. J. Hum. Genet. *96*, 432–439.

Ghabrial, A.S., Levi, B.P., and Krasnow, M.A. (2011). A systematic screen for tube morphogenesis and branching genes in the *Drosophila* tracheal system. PLoS Genet. *7*, e1002087.

Giachello, C.N.G., and Baines, R.A. (2015). Inappropriate Neural Activity during a Sensitive Period in Embryogenesis Results in Persistent Seizure-like Behavior. Curr. Biol. *25*, 2964–2968.

Goellner, B., and Aberle, H. (2012). The synaptic cytoskeleton in development and disease. Dev. Neurobiol. *72*, 111–125.

Grueber, W.B., Jan, L.Y., and Jan, Y.N. (2002). Tiling of the *Drosophila* epidermis by multidendritic sensory neurons. Development *129*, 2867–2878.

Han, C., Jan, L.Y., and Jan, Y.N. (2011). Enhancer-driven membrane markers for analysis of nonautonomous mechanisms reveal neuron-glia interactions in *Drosophila*. Proc. Natl. Acad. Sci. USA *108*, 9673–9678.

Hay, J.C., Chao, D.S., Kuo, C.S., and Scheller, R.H. (1997). Protein interactions regulating vesicle transport between the endoplasmic reticulum and Golgi apparatus in mammalian cells. Cell *89*, 149–158.

Hay, J.C., Klumperman, J., Oorschot, V., Steegmaier, M., Kuo, C.S., and Scheller, R.H. (1998). Localization, dynamics, and protein interactions reveal distinct roles for ER and Golgi SNAREs. J. Cell Biol. *141*, 1489–1502.

Horton, A.C., Rácz, B., Monson, E.E., Lin, A.L., Weinberg, R.J., and Ehlers, M.D. (2005). Polarized secretory trafficking directs cargo for asymmetric dendrite growth and morphogenesis. Neuron *48*, 757–771.

Jan, Y.N., and Jan, L.Y. (2010). Branching out: mechanisms of dendritic arborization. Nat. Rev. Neurosci. *11*, 316–328.

Jones, B., Jones, E.L., Bonney, S.A., Patel, H.N., Mensenkamp, A.R., Eichenbaum-Voline, S., Rudling, M., Myrdal, U., Annesi, G., Naik, S., et al. (2003). Mutations in a Sar1 GTPase of COPII vesicles are associated with lipid absorption disorders. Nat. Genet. *34*, 29–31.

Kasumu, A., and Bezprozvanny, I. (2012). Deranged calcium signaling in Purkinje cells and pathogenesis in spinocerebellar ataxia 2 (SCA2) and other ataxias. Cerebellum *11*, 630–639.

Kloepper, T.H., Kienle, C.N., and Fasshauer, D. (2007). An elaborate classification of SNARE proteins sheds light on the conservation of the eukaryotic endomembrane system. Mol. Biol. Cell *18*, 3463–3471.

Koch, I., Schwarz, H., Beuchle, D., Goellner, B., Langegger, M., and Aberle, H. (2008). *Drosophila* ankyrin 2 is required for synaptic stability. Neuron *58*, 210–222.

Liu, C., Lin, C., Whitaker, D.T., Bakeri, H., Bulgakov, O.V., Liu, P., Lei, J., Dong, L., Li, T., and Swaroop, A. (2013). *Prickle1* is expressed in distinct cell populations of the central nervous system and contributes to neuronal morphogenesis. Hum. Mol. Genet. *22*, 2234–2246.

Lowe, S.L., Peter, F., Subramaniam, V.N., Wong, S.H., and Hong, W. (1997). A SNARE involved in protein transport through the Golgi apparatus. Nature *389*, 881–884.

McNew, J.A., Sogaard, M., Lampen, N.M., Machida, S., Ye, R.R., Lacomis, L., Tempst, P., Rothman, J.E., and Söllner, T.H. (1997). Ykt6p, a prenylated SNARE essential for endoplasmic reticulum-Golgi transport. J. Biol. Chem. *272*, 17776–17783.

McNew, J.A., Parlati, F., Fukuda, R., Johnston, R.J., Paz, K., Paumet, F., Söllner, T.H., and Rothman, J.E. (2000). Compartmental specificity of cellular membrane fusion encoded in SNARE proteins. Nature *407*, 153–159.

Melia, T.J., Weber, T., McNew, J.A., Fisher, L.E., Johnston, R.J., Parlati, F., Mahal, L.K., Söllner, T.H., and Rothman, J.E. (2002). Regulation of membrane fusion by the membrane-proximal coil of the t-SNARE during zippering of SNAREpins. J. Cell Biol. *158*, 929–940.

Muona, M., Berkovic, S.F., Dibbens, L.M., Oliver, K.L., Maljevic, S., Bayly, M.A., Joensuu, T., Canafoglia, L., Franceschetti, S., Michelucci, R., et al. (2015). A recurrent de novo mutation in *KCNC1* causes progressive myoclonus epilepsy. Nat. Genet. *47*, 39–46.

Palade, G. (1975). Intracellular aspects of the process of protein synthesis. Science *189*, 347–358.

Parker, L., Padilla, M., Du, Y., Dong, K., and Tanouye, M.A. (2011). *Drosophila* as a model for epilepsy: *bss* is a gain-of-function mutation in the *para* sodium channel gene that leads to seizures. Genetics *187*, 523–534.

Parlati, F., McNew, J.A., Fukuda, R., Miller, R., Söllner, T.H., and Rothman, J.E. (2000). Topological restriction of SNARE-dependent membrane fusion. Nature *407*, 194–198.

Pfenninger, K.H. (2009). Plasma membrane expansion: a neuron's Herculean task. Nat. Rev. Neurosci. *10*, 251–261.

Pielage, J., Fetter, R.D., and Davis, G.W. (2005). Presynaptic spectrin is essential for synapse stabilization. Curr. Biol. *15*, 918–928.

Pielage, J., Cheng, L., Fetter, R.D., Carlton, P.M., Sedat, J.W., and Davis, G.W. (2008). A presynaptic giant ankyrin stabilizes the NMJ through regulation of presynaptic microtubules and transsynaptic cell adhesion. Neuron *58*, 195–209.

Pielage, J., Bulat, V., Zuchero, J.B., Fetter, R.D., and Davis, G.W. (2011). Hts/Adducin controls synaptic elaboration and elimination. Neuron *69*, 1114–1131.

Praschberger, R., Balint, B., Mencacci, N.E., Hersheson, J., Rubio-Agusti, I., Kullmann, D.M., Bettencourt, C., Bhatia, K., and Houlden, H. (2015). Expanding the phenotype and genetic defects associated with the *GOSR2* gene. Mov. Disord. Clin. Pract. *2*, 271–273.

Ramón y Cajal, S. (1906). The structure and connexions of neurons [Nobel lecture]. Nobel Media AB 2014. 25 May 2017. https://www.nobelprize.org/nobel_prizes/medicine/laureates/1906/cajal-lecture.html.

Rivera, V.M., Wang, X., Wardwell, S., Courage, N.L., Volchuk, A., Keenan, T., Holt, D.A., Gilman, M., Orci, L., Cerasoli, F., Jr., et al. (2000). Regulation of protein secretion through controlled aggregation in the endoplasmic reticulum. Science *287*, 826–830.

Roos, J., Hummel, T., Ng, N., Klämbt, C., and Davis, G.W. (2000). *Drosophila* Futsch regulates synaptic microtubule organization and is necessary for synaptic growth. Neuron *26*, 371–382.

Schwarz, K., Iolascon, A., Verissimo, F., Trede, N.S., Horsley, W., Chen, W., Paw, B.H., Hopfner, K.-P., Holzmann, K., Russo, R., et al. (2009). Mutations

affecting the secretory COPII coat component *SEC23B* cause congenital dyserythropoietic anemia type II. Nat. Genet. *41*, 936–940.

Shim, J., Newman, A.P., and Ferro-Novick, S. (1991). The *BOS1* gene encodes an essential 27-kD putative membrane protein that is required for vesicular transport from the ER to the Golgi complex in yeast. J. Cell Biol. *113*, 55–64.

Stephan, R., Goellner, B., Moreno, E., Frank, C.A., Hugenschmidt, T., Genoud, C., Aberle, H., and Pielage, J. (2015). Hierarchical microtubule organization controls axon caliber and transport and determines synaptic structure and stability. Dev. Cell *33*, 5–21.

Sutton, R.B., Fasshauer, D., Jahn, R., and Brunger, A.T. (1998). Crystal structure of a SNARE complex involved in synaptic exocytosis at 2.4 A resolution. Nature *395*, 347–353.

Tao, H., Manak, J.R., Sowers, L., Mei, X., Kiyonari, H., Abe, T., Dahdaleh, N.S., Yang, T., Wu, S., Chen, S., et al. (2011). Mutations in prickle orthologs cause seizures in flies, mice, and humans. Am. J. Hum. Genet. *88*, 138–149.

van Egmond, M.E., Verschuuren-Bemelmans, C.C., Nibbeling, E.A., Elting, J.W.J., Sival, D.A., Brouwer, O.F., de Vries, J.J., Kremer, H.P., Sinke, R.J., Tijssen, M.A., and de Koning, T.J. (2014). Ramsay Hunt syndrome: clinical characterization of progressive myoclonus ataxia caused by *GOSR2* mutation. Mov. Disord. *29*, 139–143.

van Egmond, M.E., Kuiper, A., Elting, J.W.J., Brouwer, O.F., de Koning, T.J., and Tijssen, M.A.J. (2015). Cortical Myoclonus in a Young Boy with *GOSR2* Mutation Mimics Chorea. Mov. Disord. Clin. Pract. *2*, 61–63.

Varlamov, O., Volchuk, A., Rahimian, V., Doege, C.A., Paumet, F., Eng, W.S., Arango, N., Parlati, F., Ravazzola, M., Orci, L., et al. (2004). i-SNAREs: inhibitory SNAREs that fine-tune the specificity of membrane fusion. J. Cell Biol. *164*, 79–88.

Volchuk, A., Ravazzola, M., Perrelet, A., Eng, W.S., Di Liberto, M., Varlamov, O., Fukasawa, M., Engel, T., Söllner, T.H., Rothman, J.E., and Orci, L. (2004). Countercurrent distribution of two distinct SNARE complexes mediating transport within the Golgi stack. Mol. Biol. Cell *15*, 1506–1518.

Weber, T., Zemelman, B.V., McNew, J.A., Westermann, B., Gmachl, M., Parlati, F., Söllner, T.H., and Rothman, J.E. (1998). SNAREpins: minimal machinery for membrane fusion. Cell *92*, 759–772.

Xu, D., Joglekar, A.P., Williams, A.L., and Hay, J.C. (2000). Subunit structure of a mammalian ER/Golgi SNARE complex. J. Biol. Chem. *275*, 39631–39639.

Ye, B., Zhang, Y., Song, W., Younger, S.H., Jan, L.Y., and Jan, Y.N. (2007). Growing dendrites and axons differ in their reliance on the secretory pathway. Cell *130*, 717–729.

Cell Reports, Volume *21*

Supplemental Information

Mutations in Membrin/*GOSR2* Reveal Stringent Secretory Pathway Demands of Dendritic Growth and Synaptic Integrity

Roman Praschberger, Simon A. Lowe, Nancy T. Malintan, Carlo N.G. Giachello, Nian Patel, Henry Houlden, Dimitri M. Kullmann, Richard A. Baines, Maria M. Usowicz, Shyam S. Krishnakumar, James J.L. Hodge, James E. Rothman, and James E.C. Jepson

A

t-SNARE	Sed5	Sec22	Bos1
WT	1.0x	1.0±0.52x	1.2±0.39x
G176W	1.0x	1.0±0.42x	1.3±0.36x
D196del	1.0x	0.9±0.42x	1.0±0.51x

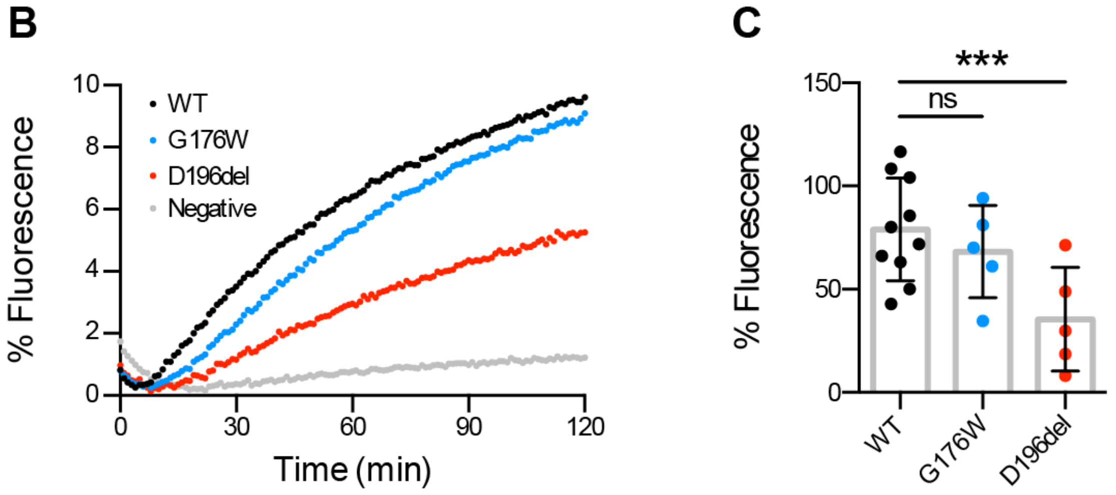

Figure S1. Liposome t-SNARE stoichiometry and liposome fusion assay after 4°C preincubation, Related to Figure 1

(A) Stoichiometry of the pre-assembled t-SNARE complexes containing either wild type (WT) or G176W/D196del mutant Bos1 determined by densitometry.

(B) Example traces of experiment as in Figure 1C with the modification that prior to measuring the fusion kinetics at 37°C the reactions were pre-incubated overnight at 4°C.

(C) Endpoint (120 min) quantification of experiment as described in (B), normalized to WT. n = 10, 5, 5 for WT, G176W, D196del. Replicate values, mean and SD are shown. *** represents $p < 0.001$, ns = not significant ($p > 0.05$); ANOVA with Dunnett's multiple comparison test.

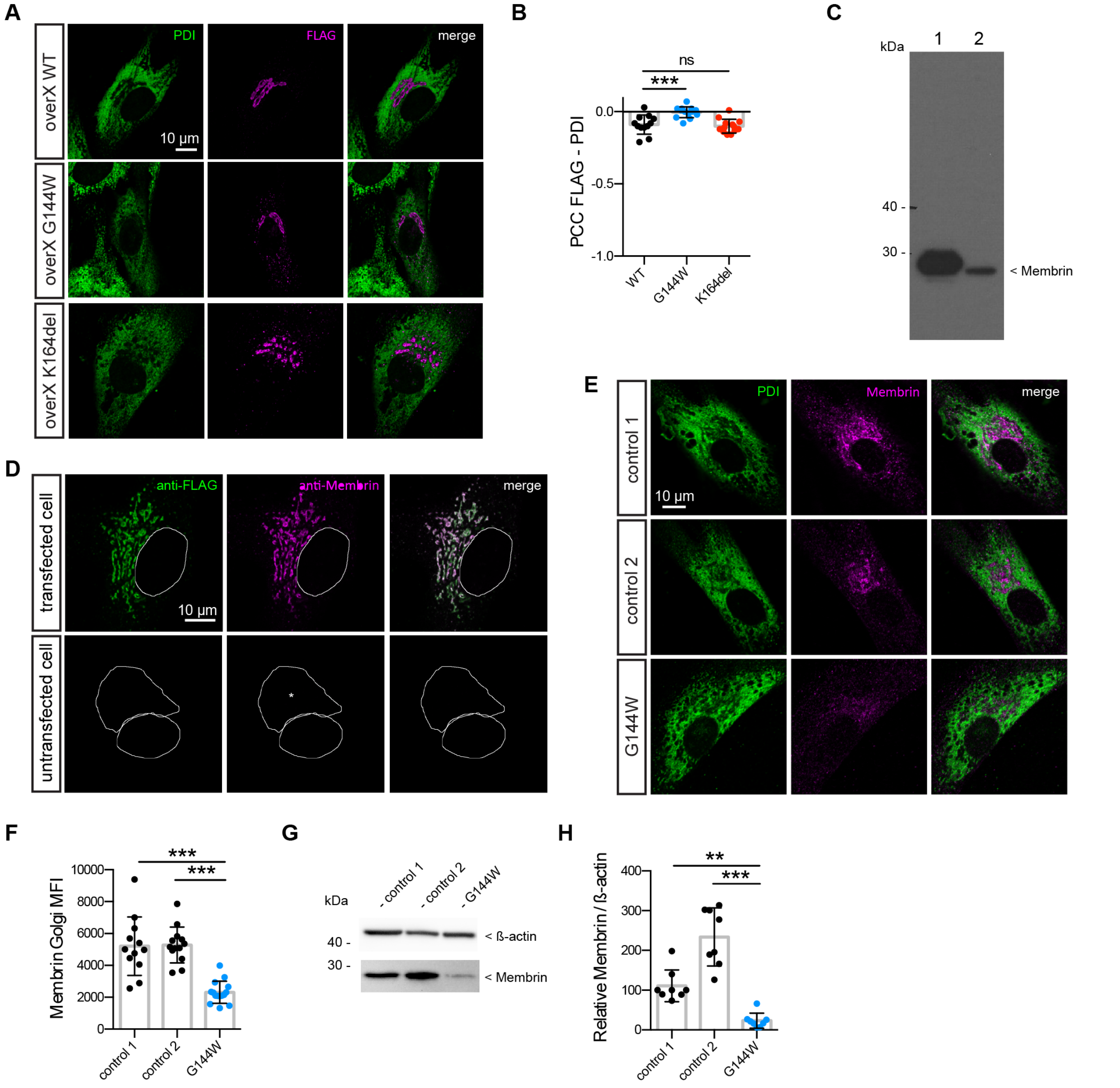

Figure S2. Membrin subcellular localization, antibody validation and quantification, Related to Figure 2
(A) FLAG-tagged WT and G144W/K164del mutant Membrin were overexpressed in control fibroblasts and co-stained for FLAG and the ER resident protein PDI. Example confocal slices are shown for each overexpressed construct.
(B) Pearson's correlation coefficients (PCC) between FLAG and PDI signal of the experiment described in (A) are shown. n = 12, 13, 13 for WT, G144W, K164del.
(C) The Membrin antibody used in Figure 2 exhibits high specificity using western blotting. Only one band at the expected molecular weight of approximately 27 kDa is apparent. Equal total protein amounts of HEK293T cell lysate (lane 2) or HEK293T cells overexpressing WT FLAG::Membrin (lane 1) were probed.
(D) The Membrin antibody used in Figure 2 with the same immuno-fluorescence protocol recognizes WT FLAG::Membrin overexpressed in control fibroblasts. Top row shows an overexpressing cell, where Membrin and FLAG signal co-localize. At the identical settings endogenous Membrin cannot be observed in an untransfected cell. Boundaries of nuclei and Golgi region (*), as seen by DIC imaging and Membrin signal at higher contrast settings respectively, are outlined.
(E) Example confocal slices of control and Membrin G144W mutant fibroblasts co-stained for endogenous Membrin and PDI.
(F) Membrin mean fluorescent intensity (MFI) in the Golgi region as demarcated by GPP130. Quantification of experiment as shown in Figure 2D. n = 12, 13, 15 for control 1, control 2, G144W.
(G) Western blot of lysates from control and G144W Membrin mutant fibroblasts.
(H) Quantification of Membrin protein from western blots as described in (G), normalized to control 1. n = 8.

Replicate values, mean and SD are shown. **, *** represent $p < 0.01$, 0.001, ns = not significant ($p > 0.05$); ANOVA with Dunnett's multiple comparison test.

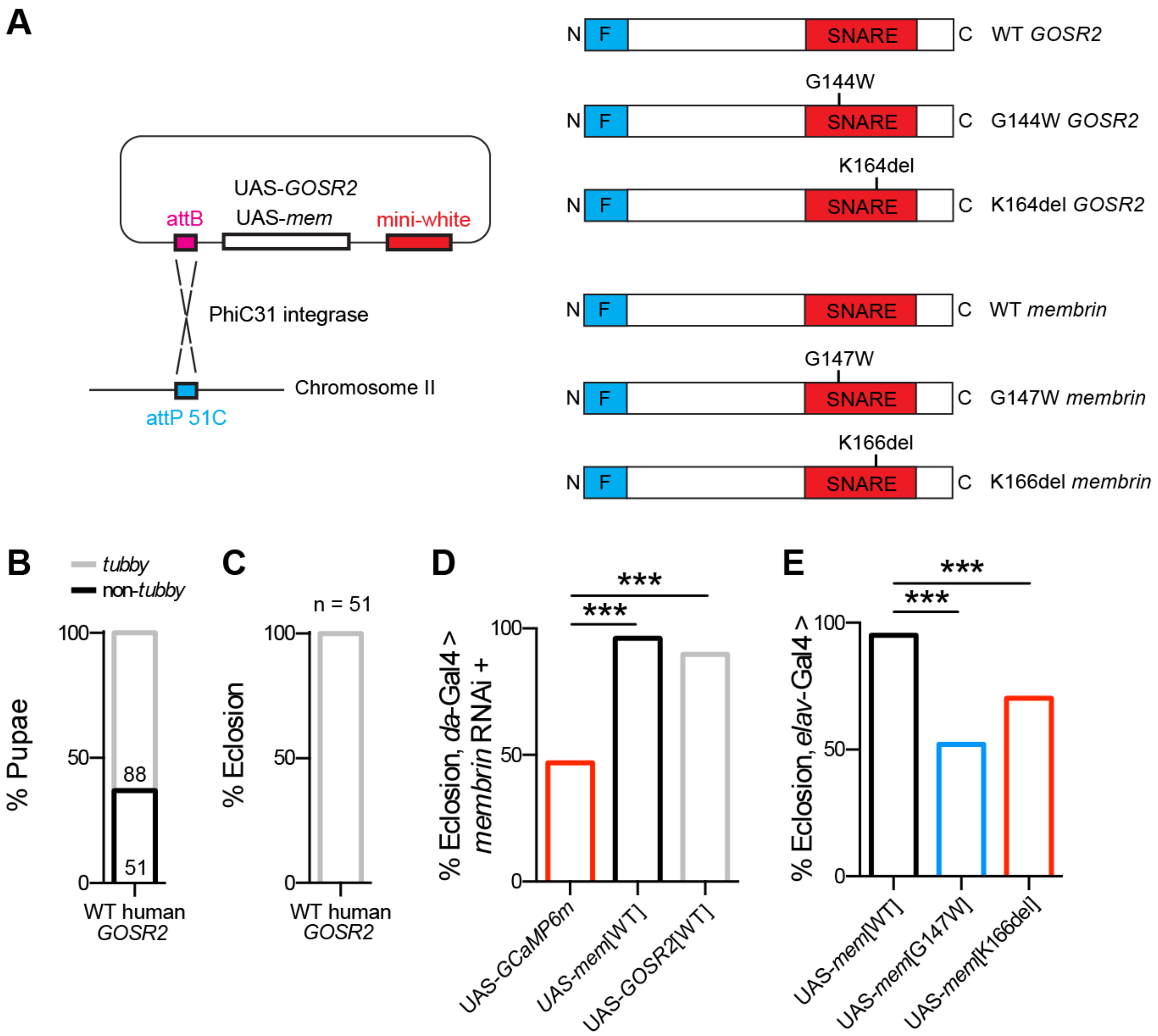

Figure S3. Human Membrin can functionally replace *Drosophila* Membrin, Related to Figure 3
(A) Schematic diagram illustrating human *GOSR2* and *Drosophila membrin* N-terminally FLAG (F) tagged transgenes and their site-specific genomic integration.
(B) FLAG-tagged human Membrin was globally expressed with the *daughterless*-Gal4 driver in a *membrin* null background according to the same strategy as in Figure 3. Rescue to the pupal stage is shown. Non-*tubby* pupae indicate the desired genotypes resulting from the genetic cross described in the Methods section.
(C) All scored non-*tubby* animals managed to eclose from their pupal cases.
(D) Membrin RNAi induced eclosion deficits can be rescued by co-overexpression of WT *Drosophila* UAS-*membrin* and WT human UAS-*GOSR2*. These effects are compared to *membrin* RNAi co-overexpression with an unrelated UAS-*GCaMP6m*, which serves to control for the presence of an additional UAS-element in these experiments. n = 164, 301, 215 for *GCaMP6m*, WT *membrin* and *GOSR2* co-overexpression.
(E) Neuronal overexpression of mutant UAS-*membrin* in wild type Membrin animals with *elav*-Gal4 resulted in reduced eclosion. n = 183, 75, 94 for UAS-*membrin*[WT]/[G147W]/[K166del].
Fisher's exact test with Bonferroni correction (D, E).

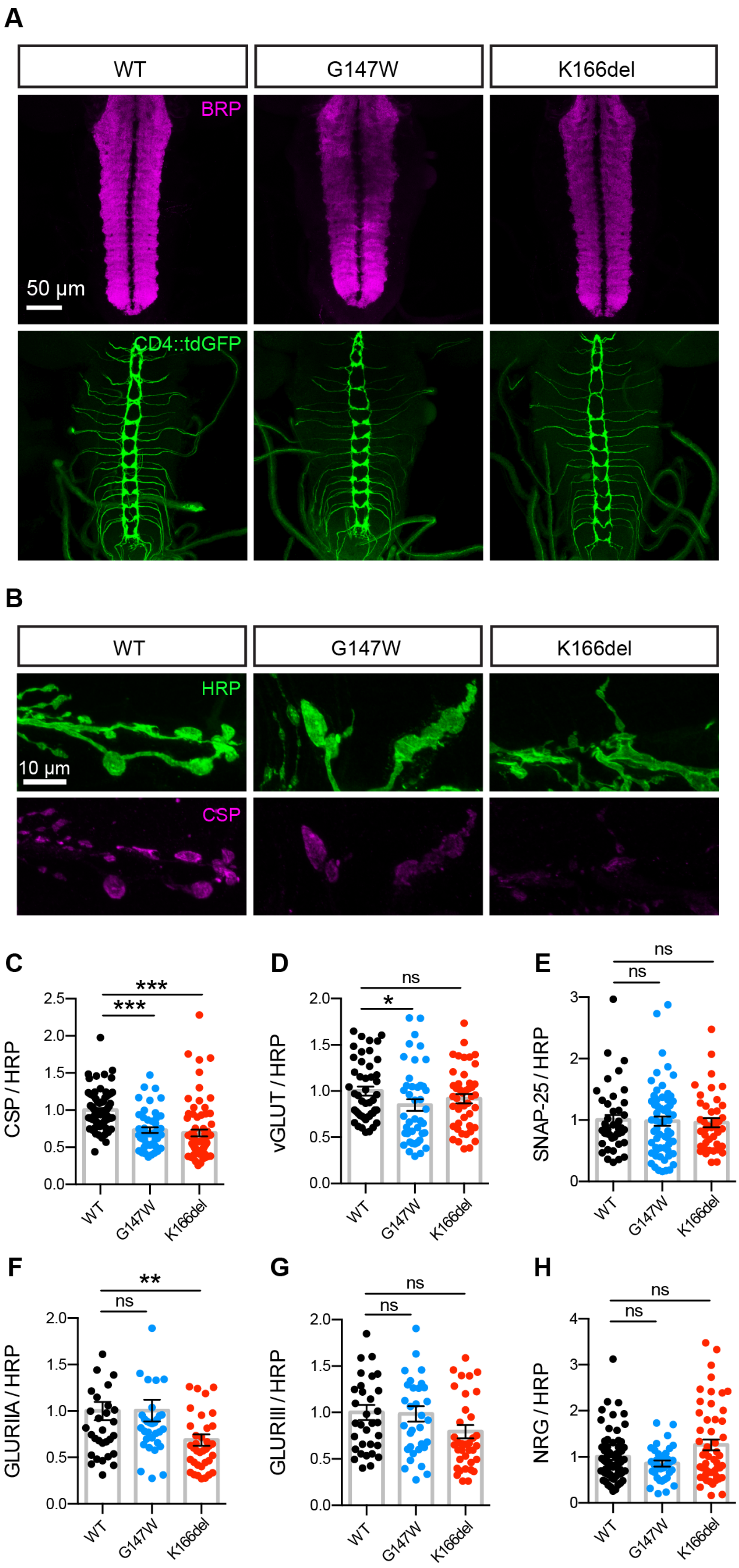
A
WT
G147W
K166del
BRP
50 µm
CD4::tdGFP
B
WT
G147W
K166del
HRP
10 µm
CSP
C
CSP / HRP
***
***
D
vGLUT / HRP
ns
*
E
SNAP-25 / HRP
ns
ns
F
GLURIIA / HRP
**
ns
G
GLURIII / HRP
ns
ns
H
NRG / HRP
ns
ns
WT
G147W
K166del

Figure S4. Sensory axonal projections and endogenous synaptic cargos, Related to Figure 4

(A) Saturated immuno-fluorescent confocal z-stacks showing projection of sensory *ppk*-neuron axons into the L3 larval ventral nerve cord (VNC). Neuropil regions in the VNC are labeled with Bruchpilot (BRP). *ppk*-neuron axons express membrane-tagged CD4::tdGFP. In Mem-WT, Mem-G147W and Mem-K166del backgrounds, no apparent defect in the growth and targeting of *ppk*-neuron axons was observed.

(B) Example confocal z-stack maximum projections of synaptic boutons immuno-labeled with antibodies against HRP and the synaptic vesicle protein Cysteine String Protein (CSP).

(C-H) Fluorescent intensity of immuno-labeled presynaptic CSP (C), vGLUT (D) and SNAP-25 (E); post-synaptic GLURIIA (F) and GLURIII (G); and trans-synaptic NRG (H) in Mem-WT, Mem-G147W and Mem-K166del synapses. Data points represent mean fluorescent intensity in a single synaptic bouton as a fraction of the corresponding HRP signal and normalized to the mean of Mem-WT. Data were acquired from ≥ 7 larvae. n = 77, 47, 72 (C); 47, 42, 45 (D); 42, 60, 41 (E); 33, 33, 37 (F); 33, 33, 37 (G); 69, 34, 52 (H) for Mem-WT/-G147W/-K166del.

Mean and SEM are shown. *, **, *** represent $p < 0.05, 0.01, 0.001$, ns = not significant ($p > 0.05$); Kruskal-Wallis test with Dunn's post-hoc test.

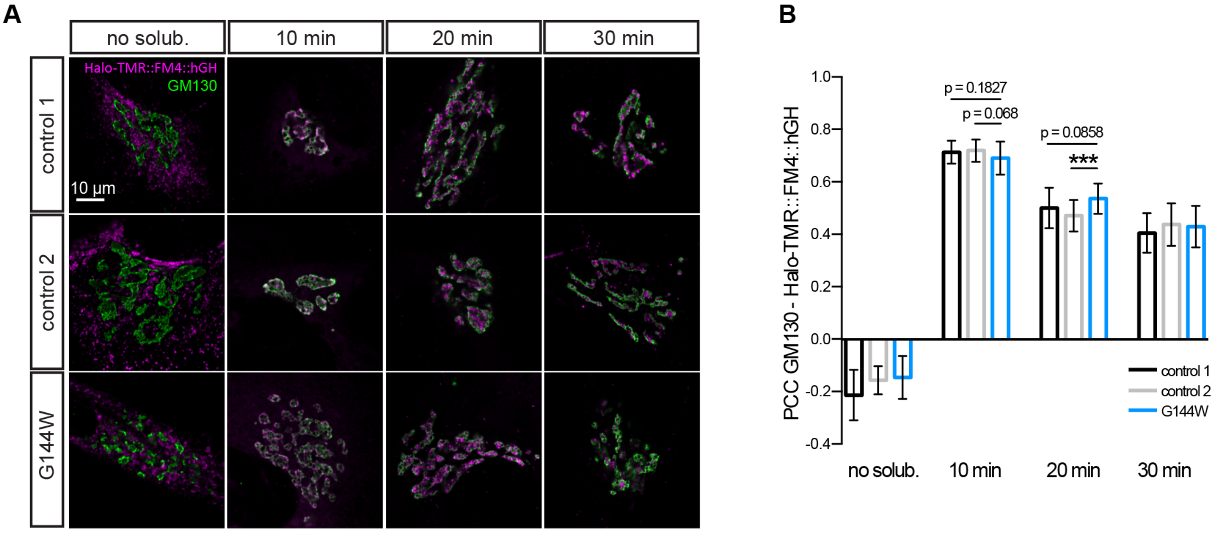

Figure S5. Golgi trafficking in patient fibroblasts, Related to Figure 5

(A) Confocal slices of control 1 & 2 and G144W mutant Membrin fibroblasts overexpressing the artificial Halo::FM4::hGH cargo loaded with TMR. When no D/D solubilizer was present the cargo remained in the ER due to aggregation of the FM4 domains. 10 min after solubilization significant colocalization with the cis-Golgi marker GM130 was apparent, which was decreased after 20 and 30 min.

(B) Quantification of experiment as described in (A). Pearson's correlation coefficients of Halo-TMR::FM4::hGH and GM130 were calculated for each time point. Number of cells quantified for control 1 & 2 and G144W are as follows: no solubilizer – 23, 21, 23; 10 min – 27, 28, 27; 20 min – 26, 29, 28; 30 min – 27, 26, 28. Mean and SD are shown.

*** represents $p < 0.001$; ns = not significant ($p > 0.05$); one-way ANOVA with Dunnett's multiple comparison test.

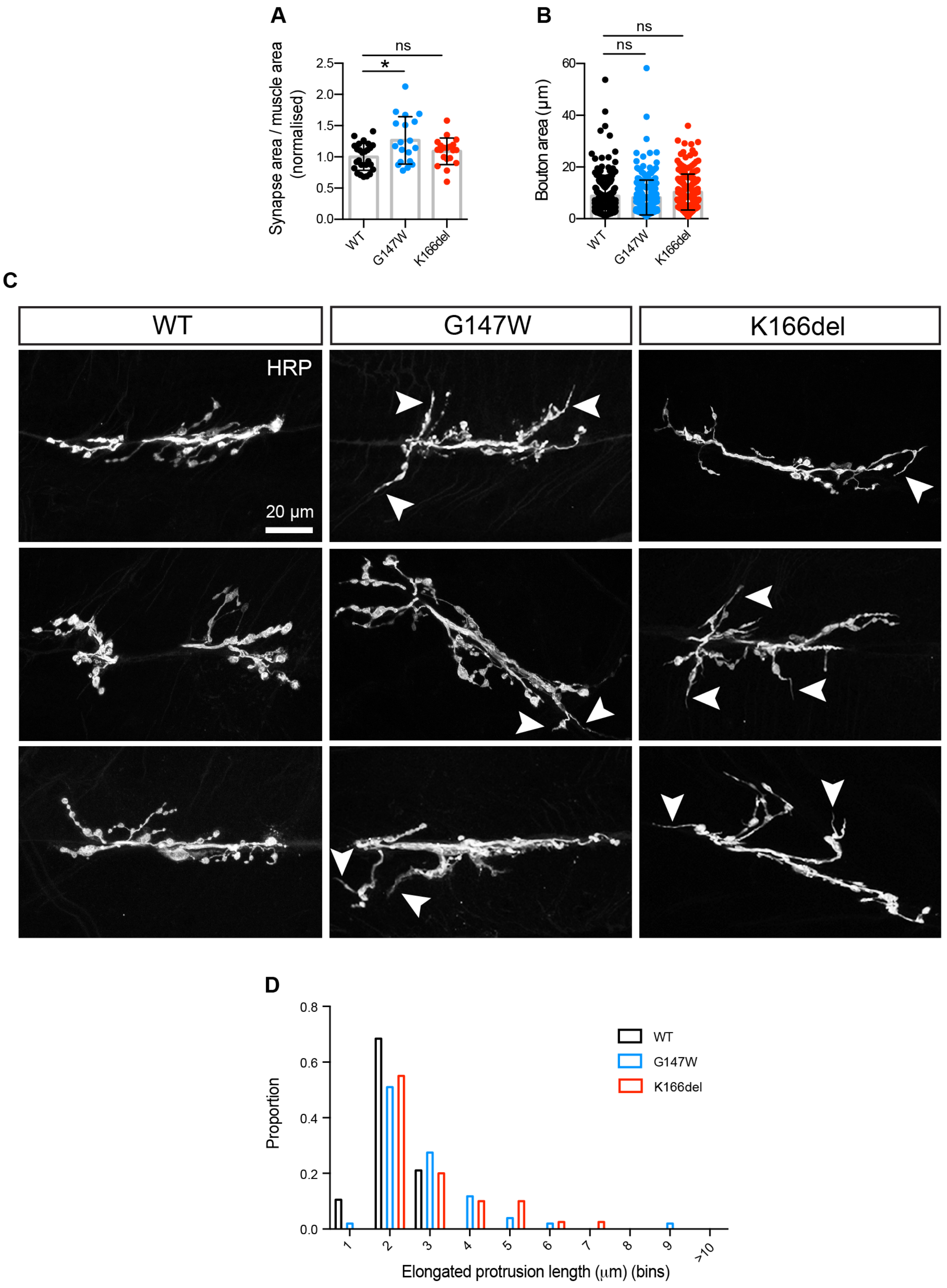

Figure S6. NMJ size and elongated protrusions, Related to Figure 5
(A) Synapse area normalized to muscle area at muscle 6/7, segment 3 of Mem-WT, Mem-G147W and Mem-K166del L3 larvae. Data are expressed relative to Mem-WT. n = 26, 19, 20 for Mem-WT/G147W/K166del.
(B) Areas of synaptic boutons of motor neurons innervating muscle 6/7, segment 3. n = 247, 237, 182 for Mem-WT/G147W/K166del.

(C) Further example confocal z-stacks of HRP-labeled motor neurons innervating muscle 6/7, segment 3 of L3 larvae, as shown in Figure 5D. Arrowheads indicate elongated protrusions frequently seen in Mem-G147W and Mem-K166del.
(D) Frequency distribution of elongated protrusion lengths at terminal boutons. Elongated protrusions in Mem-G147W and Mem-K166del exhibit a shift towards longer lengths relative to Mem-WT. n = 19, 51, 40 for Mem-WT/G147W/K166del.
Replicate values, mean and SD are shown unless otherwise stated. * represents $p < 0.05$, ns = not significant ($p > 0.05$); Kruskal-Wallis test with Dunn's post-hoc test.

Figure S7

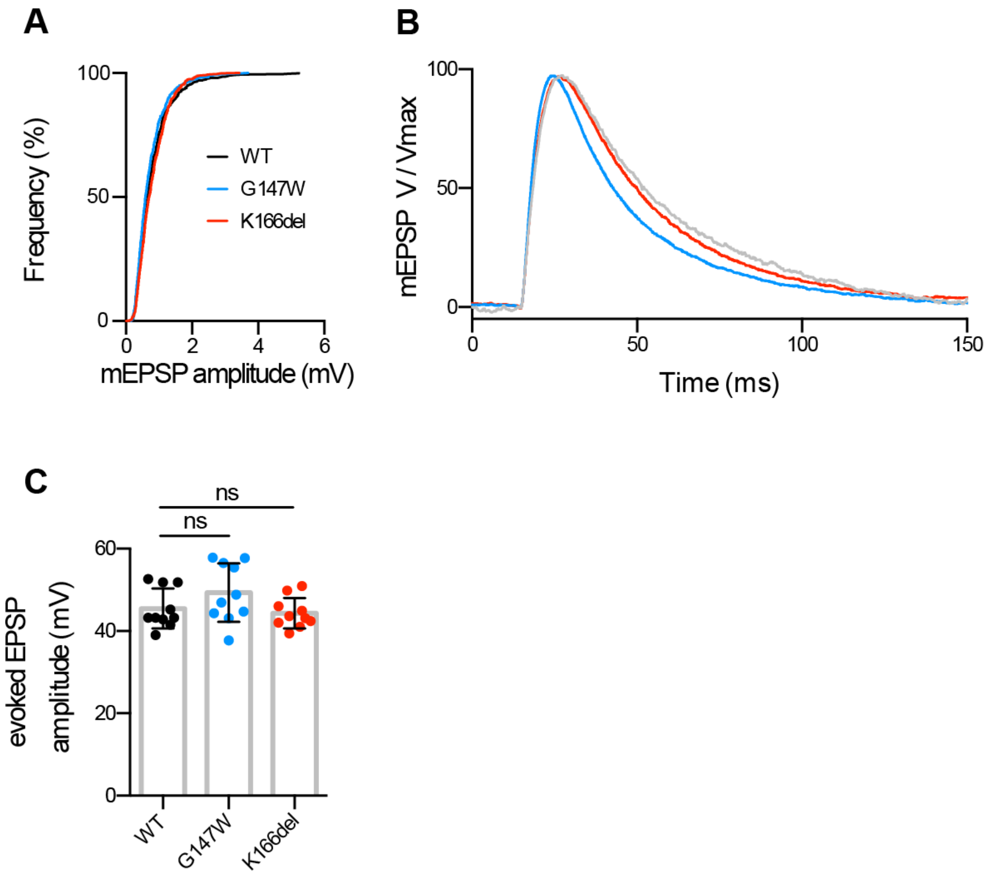

Figure S7. mEPSP amplitude, time course and evoked EPSP amplitude, Related to Figure 7
(A) Cumulative frequency plot of mEPSP amplitudes of recordings as described in Figure 7. 800 events per genotype, from 8 animals each, are shown.
(B) Average mEPSP time courses normalized to the peak are shown. There was no significant difference in the τ of a single exponential fit to the decay between Mem-G147W/Mem-K166del and Mem-WT. Each average is derived from the mean mEPSP calculated from recordings in 8 animals.
(C) Evoked EPSP amplitudes are not significantly different between Mem-WT, Mem-G147W and Mem-K166del. n = 10.
Replicate values, mean and SD are shown. ns = not significant ($p > 0.05$); one-way ANOVA with Dunnett's multiple comparison test (B, C).

SUPPLEMENTAL EXPERIMENTAL PROCEDURES

Molecular biology and Bioinformatics

GOSR2 constructs were also cloned into pcDNA3.1(-) by replacing the CDS of pcDNA3.1(-) mouse C/EBP beta (LAP) (Addgene plasmid #12557) for these inserts via NotI and KpnI (NEB), giving rise to pcDNA3.1(-)_*GOSR2*[WT], pcDNA3.1(-)_*GOSR2*[G144W] and pcDNA3.1(-)_*GOSR2*[K164del]. pGEX-2T-GST::*Sed5*, pET28a-His::*Sec22*, pET28a-His::*Bet1* and pET28a-His::*Bos1*[WT] were previously described (Parlati et al., 2000; 2002). To generate pET28a-His::*Bos1*[G176W] and pET28a-His::*Bos1*[D196del], the respective mutations were introduced with the QuickChange Site-Directed Mutagenesis Kit (Agilent Technologies). The Halo::FM4::hGH containing plasmid was previously cloned in the Rothman lab (Lavieu et al., 2013). SNARE motifs in human and *Drosophila* Membrin, as well as yeast Bos1, were identified as described previously (Kloepper et al., 2007) and aligned with Clustal Omega (McWilliam et al., 2013). Conserved residues were highlighted using BoxShade.

Liposome fusion assays

In this assay, v-SNARE-containing liposomes are loaded with the fluorophores Nitro-2-1,3 benzoxadiazol-4yl-phosphatidylethanolamine (NBD-PE) and rhodamine-PE. Both fluorophores are lipid-bound and exhibit robust Förster resonance energy transfer. Thus, NBD fluorescence is quenched in the presence of rhodamine. When v-SNARE liposomes fuse with unlabeled t-SNARE-containing liposomes, NBD and rhodamine molecules become spatially separated, NBD is de-quenched and NBD fluorescence increases, providing a measure of liposome fusion (Struck et al., 1981). Wild-type (WT) and mutant Bos1 along with the SNARE partners Bet1, Sec22 and Sed5 (orthologous to mammalian Syntaxin-5) were purified in *Escherichia coli*. GST-Sed5 was purified using glutathione agarose affinity beads (Thermo Fisher Scientific) and the GST-tag was removed using 100U of human thrombin (Sigma) with overnight incubation at 4°C in Buffer A (25 mM HEPES pH 7.4, 400 mM KCl, 10% glycerol, 1 mM DTT, 1% n-Octyl-β-D-glucopyranoside) containing 2 mM $CaCl_2$. The other proteins – Sec22, Bet1, WT and G176W/D196del mutant Bos1 – were purified with a His^6-tag using HisPur Ni-NTA affinity beads (Thermo Fisher Scientific) in Buffer A containing 300 mM imidazole, pH 7.5. For the t-SNARE acceptor liposomes, 27 µM of Sed5, Sec22 and WT or G176W/D196del mutant Bos1 in 500 µl were incubated overnight at 4°C and then incorporated into palmitoyl-2-oleoyl phosphatidylcholine (POPC): 1,2 dioleoyl phosphatidylserine (DOPS) at 85:15 mol% liposomes. The donor liposomes containing Bet1 (1:100 protein:lipid ratio) were prepared similarly with the lipid mix of POPC, DOPS and the fluorescent probes NBD-PE and rhodamine-PE (1.5 mol% each). All lipids were purchased from Avanti Polar lipids (Alabaster, AL, USA). Liposome fusion assay was performed by mixing 5 µl of the donor liposome (Bet1) with 45 µl of the acceptor liposome (t-SNAREs). Fusion of liposomes was monitored by the change in NBD fluorescence at 538 nm using a Flexstation 3 microplate reader (Molecular Devices). After 120 min, 10 µl of 5% w/v *n*-dodecyl-β-maltoside (Thermo Fisher Scientific) was added to lyse all vesicles to estimate the maximum NBD fluorescence (Weber et al., 1998). For experiments with the Bet1 peptide, the t-SNARE liposomes were pre-incubated at 37°C for 45 min with 50 µM peptide corresponding to the C-terminal half of Bet1 (RGSNQTIDQLGDTFHNTSVKLKRTFGNMMEMA, Vc peptide) prior to addition of Bet1 liposomes to initiate fusion.

Cell culture and transfections

Fibroblast and HEK293T cells were grown in DMEM + 10% FBS at 37°C and 5% CO_2. Fibroblast transfections were carried out with lipofectamine 2000 (Thermo Fisher Scientific), HEK293T transfections with Effectene (Qiagen).

Fibroblast imaging

The following antibodies were used for confocal imaging of fibroblasts: mouse anti-Membrin (clone 25, BD Biosciences; This antibody was raised against Membrin residues 5-124 and therefore should not be affected by the G144W mutation.), mouse anti-FLAG (M2 clone, Sigma), rat anti-FLAG (Agilent Technologies), rabbit anti-GPP130 (Cambridge Bioscience), mouse anti-GM130 (clone 35, BD Biosciences) rabbit anti-PDI (Sigma), and goat anti-mouse/rabbit/rat Alexa Fluor 488/555/647 conjugated secondaries (Thermo Fisher Scientific). For Golgi trafficking studies cells were loaded with HaloTag TMR Ligand (Promega) 24 h post transfection with Halo::FM4::hGH. Subsequently ER retained cargo was released by addition of D/D solubilizer 1.5 µM (Clontech). Fixed samples were imaged with a Plan-Apochromat 63x 1.4 NA oil immersion objective on Zeiss confocal LSM710 or LSM880 microscopes.

Western blot

The following antibodies were used for western blotting: mouse anti-Membrin (clone 25, BD Biosciences), mouse anti-β-actin (clone AC-74, Sigma) and HRP-conjugated anti-mouse (Jackson Immuno). For semi-

quantitative western blots, detection was carried out with SuperSignal West Pico Chemiluminescent Substrate (Thermo Fisher Scientific) and a ChemiDocTM Imaging system (Bio-Rad). Band intensities were extracted with Image Studio Lite (Li-cor).

***Drosophila* genetics and phenotyping**
To evaluate the lethal phase of *membrin*^{1524} at the L1 and L2 larval stages, we placed this allele over the fluorescent TM3 *Kr* > GFP balancer chromosome (Casso et al., 2000), which was also backcrossed to iso31 for five generations. *GOSR2* and *membrin* transgenic flies were generated by microinjection of pUASTattB_FLAG::*GOSR2*[WT/G144W/K164del]/pUASTattB_FLAG::*membrin*[WT/G147W/K166del] into *y*[1] M{vas-int.Dm}ZH-2A w*; M{3xP3-RFP.attP'}ZH-51C embryos (Cambridge fly facility). This approach enabled us to retrieve WT and mutant UAS-*GOSR2*/UAS-*membrin* transgenic fly lines with insertions into precisely the same genomic locus (ZH-51C), which is important to provide comparable transgene expression levels (Bischof et al., 2007). In order to express WT or mutant *GOSR2* or *membrin* in a *membrin* null background the following stocks were created and crossed to each other: I. *w*[1118]; UAS-FLAG::*GOSR2*[WT/G144W/K164del]; *membrin*^{1524}/TM6B, *tb* or *w*[1118]; UAS-FLAG::*membrin*[WT/G147W/K166del]; *membrin*^{1524}/TM6B, *tb* and II. *w*[1118]; +; *membrin*^{1524}, *daughterless*-GAL4/TM6B, *tb*. Each component of these flies was outcrossed for five generations into iso31 prior to assembly by standard mating schemes. For dendritic analysis, *ppk*-CD4::tdGFP (Han et al., 2011) was incorporated into stock II. (*w*[1118]; *ppk*-CD4::tdGFP; *membrin*^{1524}, *daughterless*-Gal4/TM6B, *tb*) and crossed to stock I. To assess viability of *membrin* mutant animals, the above I x II crosses were allowed to egg-lay onto apple-juice agar plates overnight. Subsequently, eggs/embryos were counted and transferred to standard food tubes. After completion of pupation, non-*tubby* pupae were counted. Given that theoretically only one quarter of the collected egg/embryos are of the correct genotype, we used the following formula to calculate egg/embryo to pupa viability: non-*tubby* pupae/(total eggs/4). The resulting fraction was normalized to WT because we found a considerable reduction with consecutive egg-lays, presumably reflecting decreasing fertilization. Eclosion rates were determined 11 and 12 days after onset of egg laying, a time point where under our conditions non-eclosion was equal to death or imminent death in the pupal case. To assess locomotion, L3 larvae were placed in the center of a 90 mm sucrose-agar plate positioned on top of a 4 mm grid, allowed to settle for 30 s, and filmed for the next 60 s. Grid-breaks in this time period were then assessed offline.

Dendritic analysis
To extract total dendrite length and to serve as a template for the ImageJ Sholl Analysis plugin, dendrites were semi-manually traced with the ImageJ NeuronJ plugin (Ferreira et al., 2014; Meijering et al., 2004). Terminal branches were manually counted on dendrite tracings with the ImageJ multi-point tool. For FRAP experiments, L3 larvae were fillet-prepped in HL3 saline without Ca^{2+} (70 mM NaCl, 5 mM KCl, 20 mM $MgCl_2$, 10 mM $NaHCO_3$, 5 mM trehalose, 115 mM sucrose, 5 mM HEPES, pH 7.2). Fillets were transferred to a #1.5 glass bottom dished and submerged in fresh HL3 saline with a custom-made platinum wire anchor before being imaged on an inverted Zeiss confocal LSM510 with a Plan-Apochromat 20x 0.8 NA objective. Bleaching of a 50 μm^2 area encompassing major primary dendrites directly adjacent to the soma was carried out by scanning for 200 iterations with 100% 488 nm transmission. Mean fluorescence intensity of a small dendritic region contained in a 5 µm diameter circle 25 µm from the bleach border adjacent to the soma served as a read-out. This is the most distant dendritic region from either bleach margin and thus the contribution to fluorescence recovery from lateral diffusion of dendrite surface localizing CD4::tdGFP is minimized. Bleach depth in this region was consistently greater than 87%.

Immuno-histochemistry of larval neuromuscular junctions and brains
Late-stage L3 larvae were dissected in low Ca^{2+} (0.2 mM) HL3 (see above). Dissected NMJ preparations were fixed in either 4% PFA or Bouin's solution for 10-20 min at room temperature. Larval brains were dissected and immunostained as described previously (Wu and Luo, 2006). The following antibodies were used: mouse anti-BRP (clone nc82, DSHB), anti-CSP (clone 6D6, DSHB), anti-GLURIIA (clone 8B4D2, DSHB), and anti-Futsch (clone 22C10, DSHB); rabbit anti-ANK2-XL (kind gift from Herman Aberle), rabbit anti-SNAP-25 (kind gift from David Deitcher), rabbit anti-GLURIII and rabbit anti-vGLUT (kind gifts from Aaron DiAntonio), mouse anti-GFP (clone 3E6, Thermo Fisher Scientific), goat anti-HRP Alexa Fluor 488 conjugated (Jackson ImmunoResearch) and secondaries as above.
For quantification of synaptic development, all experiments were performed and analysed blind to experimental genotype. Boutons were identified using anti-HRP and anti-CSP (Zinsmaier et al., 1990). Both type 1b and type 1s boutons were included in the bouton count. The number of BRP puncta and CSP-positive boutons was quantified using the ImageJ 3D Object Counter Plugin (Bolte and Cordelières, 2006). Since CSP levels were reduced in *GOSR2*-PME model backgrounds, CSP fluorescent signals were enhanced to saturating levels in all genotypes prior to bouton counting.

NMJ electrophysiology and larval seizure assay

The membrane potential was set to -70 mV with current injection at the start of each recording. Voltage signals were low-pass filtered at 1.67 kHz (10 kHz 4 pole Bessel on Geneclamp 500, 1.7 kHz 8-pole Bessel on LHBF-48x) and digitised at 25 kHz by a CED-1401 plus A/D interface (Cambridge Electronic Design, UK) using Spike2 software (v. 5.13) (CED, Cambridge, UK). Synaptic potentials were analysed offline using Strathclyde Electrophysiology Software WinEDR (v3.5.2) and GraphPad Prism (v.6). All synaptic events were verified manually. Recordings were discarded if the initial resting membrane potential was more positive than -60 mV or varied by more than 10% throughout the recording. mEPSPs were recorded for a minimum of 5 min. Single EPSPs were evoked 10 times at a standard frequency of 0.033 Hz or trains of 5 EPSPs at 10 Hz were evoked 3 times at 0.033 Hz. Intervals and amplitudes of mEPSPs were compared by creating a cumulative distribution for each genotype of 800 measurements across 8 animals, with each animal contributing 100 values. To analyse the mEPSP waveform, a mean mEPSP was constructed for each recording from events that showed only a single clear peak and a smooth decay so as to prevent distortion of the waveform by closely-occurring mEPSPs. To induce larval seizures, a 30 V DC pulse was applied towards the larval CNS for 3 s by using a Grass S88 stimulator (Grass instruments, RI, USA). This induced seizures in wild-type and mutant animals and the time until normal crawling behavior was resumed (recovery time) served as a read-out for seizure severity.

SUPPLEMENTAL REFERENCES

Bolte, S., and Cordelières, F.P. (2006). A guided tour into subcellular colocalization analysis in light microscopy. J Microsc *224*, 213–232.

Casso, D., Ramírez-Weber, F., and Kornberg, T.B. (2000). GFP-tagged balancer chromosomes for *Drosophila melanogaster*. Mech Dev. 91, 451–454.

Ferreira T, Blackman A, Oyrer J, Jayabal A, Chung A, Watt A, Sjöström J, and van Meyel D. (2014), Neuronal morphometry directly from bitmap images. Nat Methods *11*, 982–984.

McWilliam, H., Li, W., Uludag, M., Squizzato, S., Park, Y.M., Buso, N., Cowley, A.P., and Lopez, R. (2013). Analysis Tool Web Services from the EMBL-EBI. Nucleic Acids Res. *41*, W597–W600.

Meijering, E., Jacob, M., Sarria, J.-C.F., Steiner, P., Hirling, H., and Unser, M. (2004). Design and validation of a tool for neurite tracing and analysis in fluorescence microscopy images. Cytom Part A *58*, 167–176.

Struck, D.K., Hoekstra, D., and Pagano, R.E. (1981). Use of resonance energy transfer to monitor membrane fusion. Biochemistry *20*, 4093–4099.

Wu, J.S., and Luo, L. (2006). A protocol for dissecting *Drosophila melanogaster* brains for live imaging or immunostaining. Nat Protoc *1*, 2110–2115.

Zinsmaier, K.E., Hofbauer, A., Heimbeck, G., Pflugfelder, G.O., Buchner, S., and Buchner, E. (1990). A cysteine-string protein is expressed in retina and brain of *Drosophila*. J Neurogenet *7*, 15–29.
